# Benzylic Fluorination Induced by a Charge-Transfer Complex with a Solvent-Dependent Selectivity Switch

**DOI:** 10.1021/acs.orglett.2c02050

**Published:** 2022-07-18

**Authors:** Amiera Madani, Lucia Anghileri, Matthias Heydenreich, Heiko M. Möller, Bartholomäus Pieber

**Affiliations:** † Department of Biomolecular Systems, Max-Planck-Institute of Colloids and Interfaces, Am Mühlenberg 1, 14476 Potsdam, Germany; ‡ Department of Chemistry and Biochemistry, Freie Universität Berlin, Arnimallee 22, 14195 Berlin, Germany; § Institute of Chemistry/Analytical Chemistry, University of Potsdam, Karl-Liebknecht-Strasse 24−25, 14476 Potsdam, Germany

## Abstract

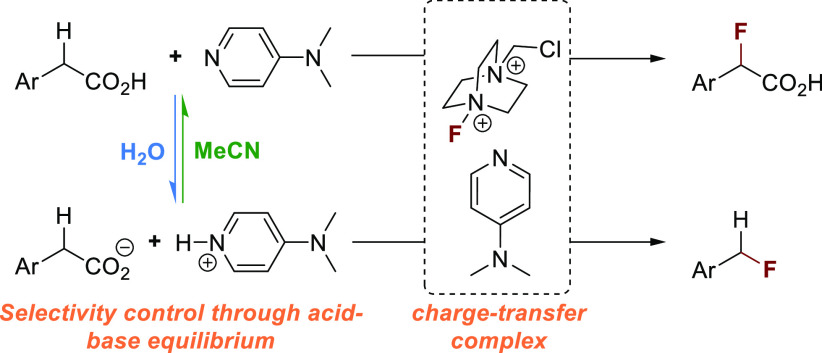

We present a divergent strategy for the fluorination of phenylacetic acid derivatives that is induced by a charge-transfer complex between Selectfluor and 4-(dimethylamino)pyridine. A comprehensive investigation of the conditions revealed a critical role of the solvent on the reaction outcome. In the presence of water, decarboxylative fluorination through a single-electron oxidation is dominant. Non-aqueous conditions result in the clean formation of α-fluoro-α-arylcarboxylic acids.

Fluorination increases the lipophilicity and metabolic stability of organic molecules, resulting in improved active pharmaceutical ingredients and agrochemicals.^1−4^ Position emission tomography (PET) of ^18^F-labeled radiopharmaceuticals is important for studying biochemical pathways and physiological processes.^5^ Consequently, the development of efficient and selective protocols for constructing C–F bonds is desirable.^6^ Nucleophilic fluorination and electrophilic fluorination dominate the field, but radical fluorinations recently gained significant momentum for two reasons.^6^ First, the restriction to hazardous radical fluorine sources, such as XeF_2_ and F_2_, was overcome by the discovery that electrophilic N–F reagents transfer fluorine atoms to carbon-centered radicals.^7^ Second, the increasing interest in synthetic radical chemistry resulted in attractive methods for generating C-centered radicals using dedicated catalysts and reagents.^8^

The formation of benzylic C(sp^3^)–F bonds using 1-(chloromethyl)-4-fluoro-1,4-diazabicyclo[2.2.2]octane-1,4-diium ditetrafluoroborate (Selectfluor)^9^ is among the most studied C–F bond formations that proceed via a radical mechanism.^6,7,10−13^ These transformations are promising tools for modifying drug candidates for preventing undesired benzylic oxidation by cytochrome P450 oxidases.^14^ Common strategies are decarboxylative fluorinations that use photocatalysts^15−18^ or silver catalysts to induce single-electron-transfer (SET) oxidation^19−21^ and the direct fluorination of benzylic C(sp^3^)–H bonds using catalysts or reagents that enable hydrogen atom transfer (HAT) (Scheme 1A).^22−26^ It must be noted that the α-fluorination of phenylacetic acids can also be carried out using Selectfluor via a silyl ketene acetal that is formed using a strong base^21,27,28^ or with the aid of catalytic amounts of a strong Lewis acid and an organic base to generate an enediolate intermediate.^29^

**Scheme 1:**
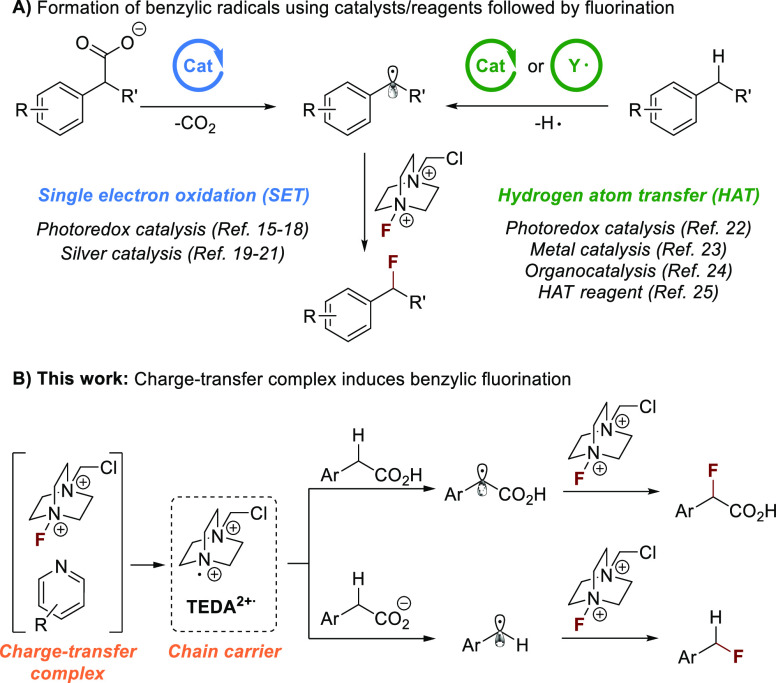
Benzylic C(sp^3^)–F Bond Formation Using Selectfluor through Radical Intermediates

A dedicated catalyst or reagent is regularly used to generate the key benzylic radical, which ultimately undergoes F atom transfer with Selectfluor to yield the desired product. Such benzylic fluorinations may also proceed through electron transfer or proton-coupled electron transfer rather than HAT.^30^ Catalyst-free benzylic fluorination of aza-heterocycles was reported to proceed through the formation of a charge-transfer (CT) complex between N-heterocyclic substrates and Selectfluor. This induces a stepwise electron/proton transfer or a concerted proton-coupled electron-transfer process.^31^ Nitrogen–fluorine halogen bonding between Selectfluor and pyridine additives was proposed to facilitate silver-catalyzed radical fluorinations, but no product was observed in the absence of the metal catalyst.^32^

We envisioned that a CT complex between Selectfluor and an aromatic N-heterocyclic compound could generate the *N*-(chloromethyl)triethylenediamine radical dication (TEDA2^+•^), a potent single-electron oxidant and hydrogen atom-transfer reagent.^33^ This would access an operationally simple and divergent strategy for the generation of benzylic carbon-centered radicals that could ultimately engage with Selectfluor to form C–F bonds. Here we present that this mechanistic blueprint can indeed be applied to achieve the direct fluorination of benzylic C(sp^3^)–H bonds that likely proceeds via a HAT mechanism and the decarboxylative formation of benzylic C(sp^3^)–F bonds through a SET process (Scheme 1B).

We started our investigations by studying whether a combination of Selectfluor and 4-(dimethylamino)pyridine (DMAP) triggers decarboxylative C–F bond formation or direct fluorination of benzylic C(sp^3^)–H bonds. We chose 2-(4-fluorophenyl)acetic acid as model substrate that serves as an ideal probe for both scenarios (Scheme 2A). To our delight, we indeed observed a mixture of both fluorination products at room temperature with good selectivity toward the decarboxylative product in an acetonitrile/water mixture. Under non-aqueous conditions, α-fluorination occurred selectively.

**Scheme 2:**
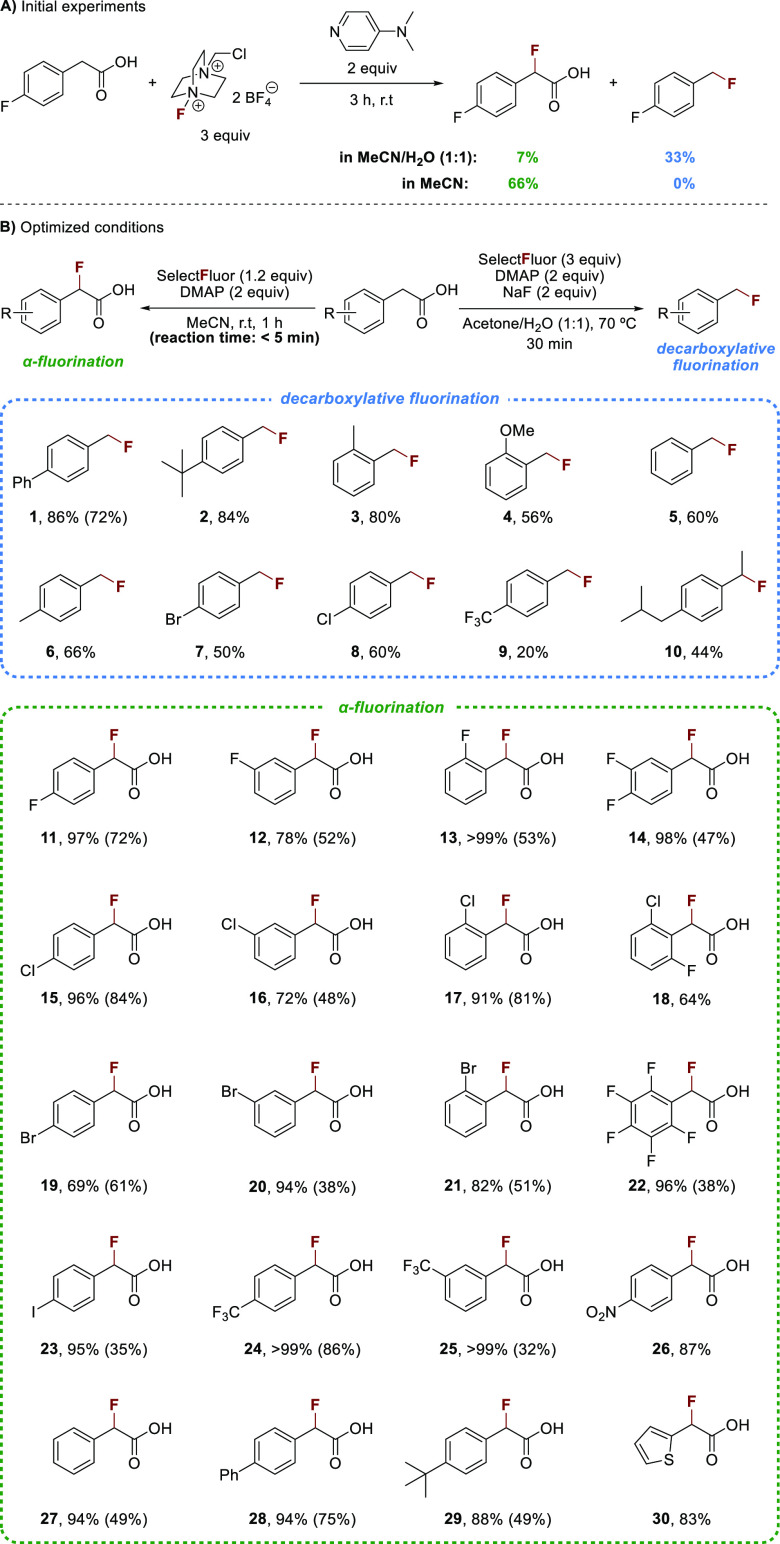
Initial Results and Scope of the Benzylic C(sp^3^)–F Bond Formation Using Selectfluor and DMAP Yields were determined by ^1^H NMR using dimethyl maleate as the internal standard. Isolated yields are in parentheses.

After considerable experimentation (see the Supporting Information), we found that the decarboxylative fluorination works best in an acetone/water (1:1) mixture using 2 equiv of DMAP and an excess of Selectfluor (3 equiv) (Scheme 2B). Also, the addition of sodium fluoride (2 equiv) and an increased temperature (70 °C) were beneficial for converting a series of phenylacetic acid derivatives to the corresponding, volatile SET products (1–10) within 30 min in moderate to good NMR yields. Unreacted starting material was observed in many cases, and no major side product could be identified (see the Supporting Information).

A combination of 2 equiv of DMAP and 1.2 equiv of Selectfluor in acetonitrile produced the α-fluorination products (11–30) at room temperature in good to excellent NMR yields (Scheme 2B). Phenylacetic acid derivatives with electron-rich and electron-deficient substituents were cleanly converted to the respective α-fluoro-α-arylacetic acids. Small amounts of unreacted starting material were detected in all cases, which were difficult to separate by column chromatography and resulted in modest isolated yields in certain cases. A reaction time of 1 h was used for practical reasons, but detailed investigations revealed that the fluorination occurs in <5 min (Tables S14 and S15). Interestingly, the carboxylic acid functionality is crucial for α-fluorination. No reaction was observed using other functional groups, such as ketones, esters, amides, boronic acid esters, or boronates (see the Supporting Information).

We propose that a mixture of DMAP and Selectfluor spontaneously produces TEDA2^+•^, which acts as a chain carrier in a SET or HAT process (Scheme 3). The radical chain is efficient for the HAT route (1.2 equiv of Selectfluor under optimized conditions), whereas the SET pathway seems to suffer from a significant amount of undesired termination events (3 equiv of Selectfluor under optimized conditions). The switch between the SET and HAT pathway is a consequence of different p*K*_a_ values of phenylacetic acids and DMAP under the applied conditions. The organic base deprotonates the carboxylic acid in an aqueous environment, enabling single-electron oxidation of the carboxylate by TEDA^2+•^. SET oxidation triggers decarboxylation to produce a C-centered radical that ultimately reacts with Selectfluor to yield the desired product and TEDA^2+•^. Phenylacetic acid derivatives have low acidity in aprotic polar solvents (p*K*_a_ of phenylacetic acid in MeNO_2_ > 19).^34^ The p*K*_a_ of the conjugated acid of pyridine derivatives in MeCN is lower.^35^ As a result, the amount of carboxylate under these conditions is negligible. This reduces the likelihood of decarboxylative SET and HAT becoming the dominant pathway.

**Scheme 3:**
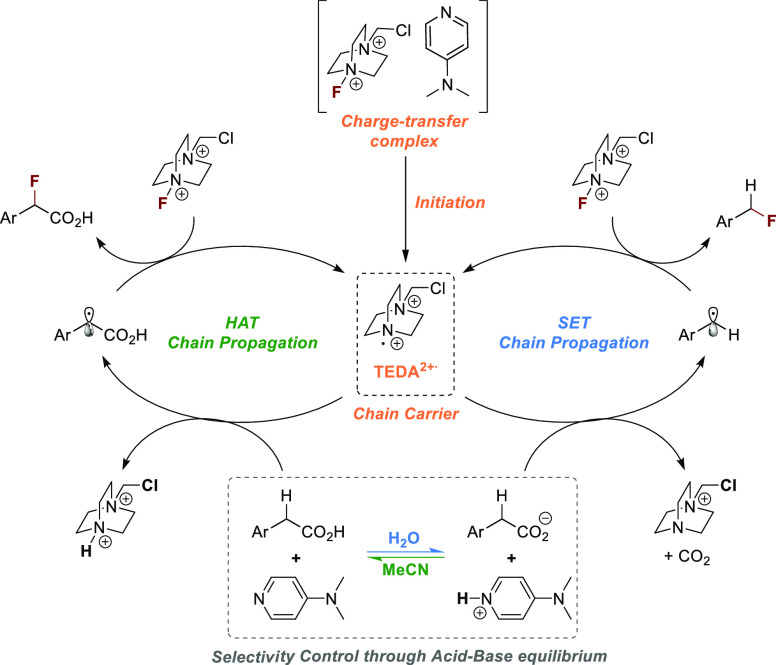
Proposed Mechanism for the Formation of Benzylic C(sp^3^)–F Bonds via the Activation of Selectfluor Using DMAP

While there are no plausible alternatives to the SET mechanism, α-fluoro-α-arylacetic acid formation may occur through a more traditional “electrophilic” fluorination mechanism rather than HAT. Surprisingly, we also observed product formation using NFSI (*N*-fluorobenzenesulfonimide) instead of Selectfluor (Table S10), which was, to the best of our knowledge, never reported to produce a radical chain carrier. We therefore carried out a series of experiments to shed some light on the mechanism. An enediolate species was not observed upon treatment of phenylacetic acid with DMAP in MeCN-*d*_3_ (Figure S3). This is in agreement with a study that shows that the formation of such species using organic bases requires strong Lewis acids as activators.^29^ However, a radical clock experiment did not prove the formation of the proposed benzylic radical intermediate (Scheme S2). The addition of the radical scavenger TEMPO resulted in the consumption of the substrate, but no trace of the fluorination product was formed, indicative of a radical mechanism (Scheme S3). Competition experiments involving deuterium-labeled substrates indicate that C–H bond cleavage is the rate-determining step (Scheme S4). In addition, competitive experiments between phenylacetic acids with different substituents on the aromatic ring showed that electron-rich aromatic systems react slower (Scheme S5). These experiments suggest that the reaction does not proceed through a SET oxidation followed by deprotonation.

The simple preparation of α-fluoro-α-arylcarboxylic acids through our method is attractive compared to the most common approach for synthesizing such scaffolds that requires formation of a silyl ketene acetal using a strong base, followed by treatment with Selectfluor.^21,27,28^ Our α-fluorination is not sensitive to air or moisture, works with bench-stable reagents, and produces the desired products in up to quantitative yields as determined by ^19^F NMR (isolated yields range from 32% to 86%). This operational simplicity is promising for the synthesis of ^18^F-labeled radiopharmaceuticals using [^18^F]Selectfluor. In particular, the short reaction times are ideally suited for such applications, due to the short half-lives of ^18^F-labeled radionuclides (110 min).^36^ Monitoring the fluorination of 4-*tert*-butylphenylacetic acid using in situ FTIR spectroscopy showed that the reaction forms the desired product instantaneously once Selectfluor is added to a solution of DMAP and the substrate in MeCN (Figure 1A). Fast consumption of the fluorination reagent was also monitored in the absence of the substrate (Figure S6). This supports our mechanistic proposal that the fluorine source and the organic base form a reactive, labile species that leads to productive fluorination or, if no substrate is present in the reaction mixture, to degradation products. ^1^H NMR experiments using Teflon inserts showed the formation of a DMAP·HF adduct upon mixing DMAP and Selectfluor along with several unidentified compounds that are likely N-fluorinated DMAP derivatives.^37^ We carried out a series of “delayed addition” experiments to clarify if the order of reagent addition is crucial for successful fluorination (Figure 1B). When Selectfluor and DMAP were mixed in MeCN and the substrate was added after 30 min, no reaction was observed. Premixing the substrate with Selectfluor or DMAP is possible.

**Figure 1:**
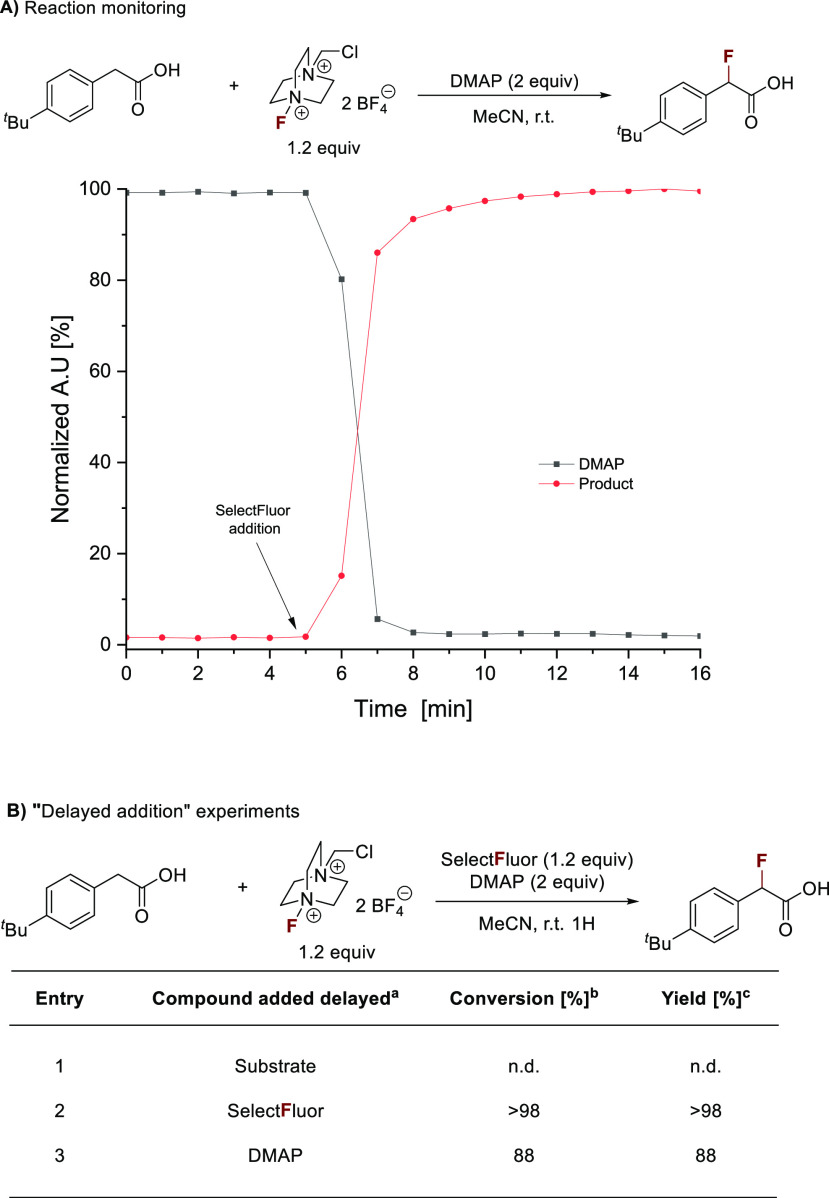
Practical aspects of the HAT protocol. (A) Reaction monitoring using in situ FTIR spectroscopy. (B) Delayed addition experiments.

Finally, we sought to study if a strong electron-donating substituent on the pyridine base is crucial for reactivity. Exchanging DMAP with 4-aminopyridine or 4-methoxypyridine reduced the efficacy of this reaction (Table 1). Modest conversions were obtained with pyridine, emphasizing that a strong Lewis basicity is key for the generation of TEDA^2+•^.

**Table 1:**
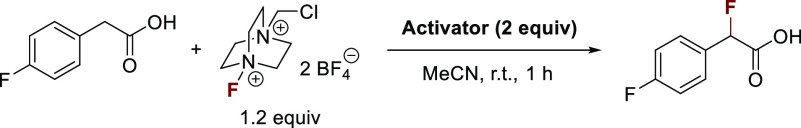

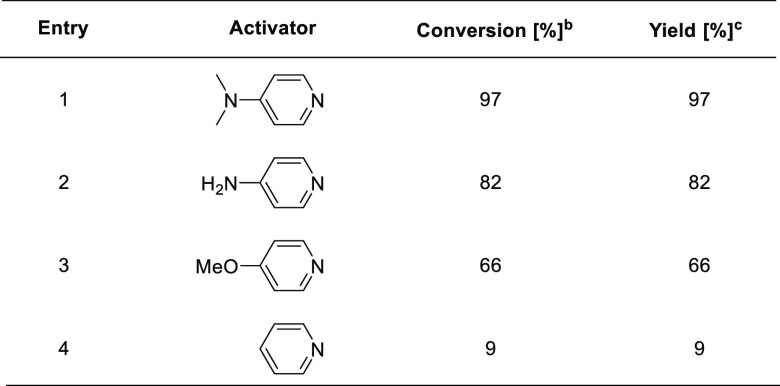
Influence of Lewis Basicity on N–F Bond Activation of Selectfluor^a^

a Reaction conditions: 4-fluorophenylacetlc acid (0.3 mmol), Selectfluor (0.36 mmol), DMAP (0.6 mmol), MeCN (1.5 mL), rt, 4 h.

b Converaion of 4-fluorophenylacetic acid determined by ^1^H NMR using dimethyl maleate as an internal standard.

c NMR yield determined by ^1^H NMR using dimethyl maleate as an internal standard.

In summary, we developed a new strategy for the formation of benzylic C(sp^3^)–F bonds that is proposed to proceed via the formation of TEDA^2+•^ from Selectfluor and 4-(dimethylamino)pyridine. Controlling the p*K*_a_ of phenylacetic acid derivatives via the reaction media enables switching between reaction mechanisms that enables the selective formation of different products. Under aqueous conditions, a decarboxylative fluorination was observed, whereas non-aqueous conditions allow for direct fluorination of benzylic C(sp^3^)–H bonds. This enables a facile and clean formation of α-fluoro-α-arylacetic acids within a few minutes at room temperature.

## Supporting Information Available

Experimental procedures and characterization data (PDF)
