# Supplementary material for: Benzylic Fluorination Induced by a Charge-Transfer Complex with a Solvent-Dependent Selectivity Switch
Source: Org Lett. 2022 Jul 18;24(29):5376–80. doi: 10.1021/acs.orglett.2c02050 (PMC9344467; doi:10.1021/acs.orglett.2c02050)
Supplement: Supplementary file 1 — ol2c02050_si_001.pdf [file ol2c02050_si_001.pdf]

# Benzylic fluorination induced by a charge-transfer complex with a solvent-dependent selectivity switch

Amiera Madani, [†,‡] Lucia Anghileri, [†,‡] Matthias Heydenreich, [§] Heiko M. Möller, [§] and Bartholomäus Pieber[*†]

[†] Department of Biomolecular Systems, Max-Planck-Institute of Colloids and Interfaces, Am Mühlenberg 1, 14476 Potsdam, Germany

[‡] Department of Chemistry and Biochemistry, Freie Universität Berlin, Arnimallee 22, 14195 Berlin, Germany

[§] Institute of Chemistry/Analytical Chemistry, University of Potsdam, Karl-Liebknecht-Strasse 24–25, 14476 Potsdam, Germany

**Correspondence to: bartholomaeus.pieber@mpikg.mpg.de**

1. General remarks ... 2
2. Initial experiments ... 2
3. Decarboxylative fluorination ... 3
3.1 Solvent screening ... 4
3.2 Temperature & Additive screening ... 5
3.3 Reaction time and concentration ... 6
3.4 Control studies ... 6
3.5 Scope ... 7
4. α-fluorination ... 13
4.1 N–F Reagents ... 14
4.2 Solvent screening ... 14
4.3 Optimization of the equivalents of Selectfluor and DMAP ... 15
4.4 Screening of different activators ... 15
4.5 Concentration and reaction time studies ... 16
4.6 Mechanistic investigations ... 17
4.7 Delayed addition experiments ... 19
4.8 Reaction monitoring using a ReactIR ... 19
4.9 NMR experiments ... 21
5 Scope ... 25
6 References ... 32
Copies of NMR spectra of isolated compounds ... 33

# 1. General remarks

Substrates, reagents, and solvents were purchased from commercial suppliers and used without further purification. NMR spectra were recorded using a Varian 400 MHz (400, 100 and 376 MHz respectively for $^{1}$H-, $^{13}$C- and $^{19}$F-NMR) or a Bruker NEO 500 MHz spectrometer equipped with a H/F/C/N-TCI-Prodigy probe and are reported in ppm relative to the residual solvent peaks. Peaks are reported as: s = singlet, d = doublet, t = triplet, q = quartet, m = multiplet or unresolved, with coupling constants in Hz. Reaction monitoring via FTIR analysis was performed with a ReactIR™ 15 (Mettler-Toledo) console, with a DST 9.5mm SiComp™ probe attached. Analytical thin layer chromatography (TLC) was carried out using pre-coated TLC-sheets, ALUGRAM Xtra SIL G/UV254 sheets (Macherey-Nagel) and visualized with 254 nm light. Purification of synthesized compounds was carried out by flash chromatography on a Reveleris X2 Flash Chromatography System from GRACE. A prepacked column with 12 g, 40 μm silica gel was used at a 30 mL/min elution flow rate. Silica 60 M (0.04-0.063 mm) silica gel (Macherey-Nagel) was used for dry loading of the crude compounds. High resolution mass spectra (HRMS, *m/z*) were recorded on a Bruker MicroTOF spectrometer using positive electrospray ionization (ESI).

# 2. Initial experiments

**Table S1.** Initial experiments[a]

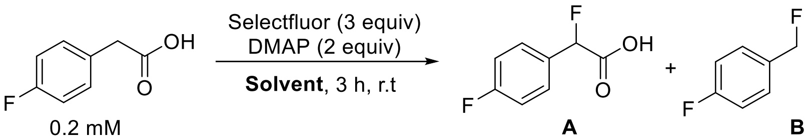


| Entry | Solvent | Conversion [%][b] | A [%][c] | B [%][c] |
|---|---|---|---|---|
| 1 | MeCN | 66 | 66 | n.d. |
| 2 | MeCN:$H_2O$ (1:1) | 73 | 7 | 33 |

[a]Reaction conditions: 4-fluorophenylacetic acid (0.3 mmol), Selectfluor (0.9 mmol), DMAP (0.6 mmol), solvent (1.5 mL, degassed) under nitrogen atmosphere at r.t for 3 h. [b]Conversion of 4-biphenylacetic acid determined by $^{1}$H-NMR using dimethyl maleate as an internal standard. [c]NMR yield determined by $^{1}$H-NMR using dimethyl maleate as an internal standard. n.d. = not detected.

**Table S2.** Control studies in MeCN[a]

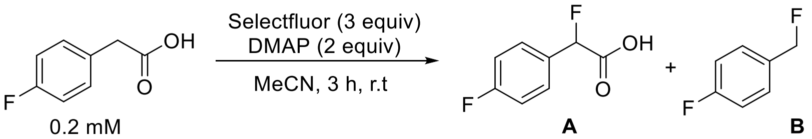


| Entry | Variation | Conversion [%][b] | A [%][c] | B [%][c] |
|---|---|---|---|---|
| 1 | No degassing | 80 | 80 | n.d |
| 2 | No Selectfluor | n.d | n.d | n.d |
| 3 | No DMAP | n.d | n.d | n.d |

[a]Reaction conditions: 4-fluorophenylacetic acid (0.3 mmol), Selectfluor (0.9 mmol), DMAP (0.6 mmol) in acetonitrile (anhydrous, 1.5 mL) at r.t for 3 h. [b]Conversion of 4-biphenylacetic acid determined by $^{1}$H-NMR using dimethyl maleate as an internal standard. [c]NMR yield determined by n.d = not detected.

**Table S3.** Control studies in MeCN:$H_2O$[a]

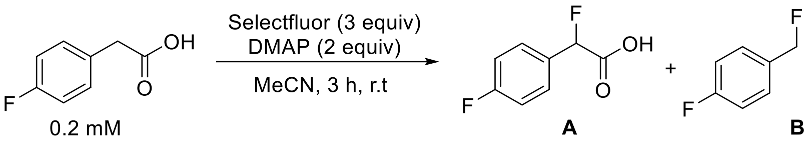


| Entry | Variation | Conversion [%][b] | A [%][c] | B [%][c] |
|---|---|---|---|---|
| 1 | No degassing | 17 | 6 | 11 |
| 2 | No Selectfluor | n.d | n.d | n.d |
| 3 | No DMAP | n.d | n.d | n.d |

[a]Reaction conditions: 4-fluorophenylacetic acid (0.3 mmol), Selectfluor (0.9 mmol), DMAP (0.6 mmol) in solvent (anhydrous, 1.5 mL) at r.t for 3 h. [b]Conversion of 4-biphenylacetic acid determined by $^1$H-NMR using dimethyl maleate as an internal standard. [c]NMR yield determined by n.d = not detected.

## 3. Decarboxylative fluorination

**General experimental procedure for optimization experiments.** An oven dried crimp top vial (19 x 100 mm) equipped with a stir bar was charged with with 4-biphenylacetic acid and Selectfluor®. The solvent mixture was added and the mixture was stirred and sonicated for 5 min. Subsequently, 4-dimethylaminopyridine (and, if indicated, the additive) was added. The vial was sealed and the reaction mixture was stirred and sonicated for 5 min. The mixture was degassed by bubbling Argon for 10 min. The reaction mixture was stirred in an oil bath at 70 °C for the respective time. Afterwards, dimethyl maleate (1 equiv.) was added. The reaction mixture was quenched with HCl (1 M, 1 mL) and extracted with $CDCl_3$ (1 mL). An aliquote (~200 µL) of the organic phase was subsequently subjected to $^1$H NMR analysis.

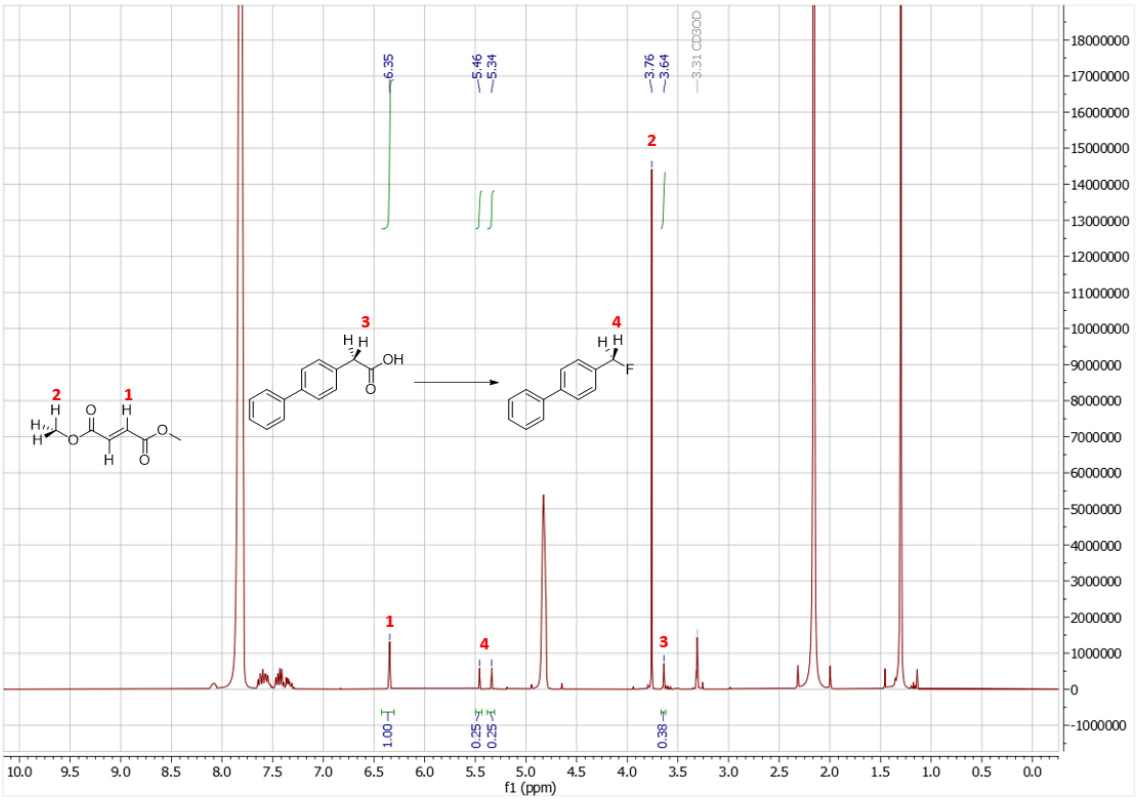

**Figure S1**. Example of a $^{1}$H-NMR spectrum for determining NMR yields.

## 3.1 Solvent screening

**Table S4.** Initial solvent screening[a]

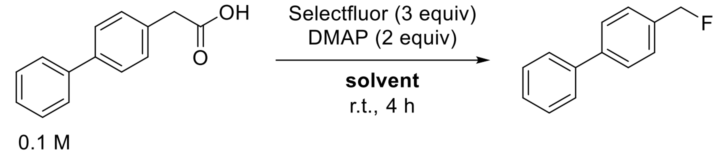


| Entry | Solvent | Conversion [%][b] | Yield [%][c] |
|---|---|---|---|
| 1 | MeCN/$H_2O$ 1:1 | 33 | 28 |
| **2** | **Acetone/ $H_2O$ 1:1** | **41** | **40** |
| 3 | EtOH/ $H_2O$ 1:1 | n.d.o[d] | 18 |
| 4 | THF/ $H_2O$ 1:1 | 14 | 8 |
| 5 | DMF/ $H_2O$ 1:1 | 44 | 36 |
| 6 | $H_2O$ | 39 | 26 |

[a]Reaction conditions: 4-biphenylacetic acid (0.1 mmol), Selectfluor (0.3 mmol), DMAP (0.2 mmol), solvent (1 mL), r.t, 4 h. [b]Conversion of 4-biphenylacetic acid determined by $^{1}$H-NMR using dimethyl maleate as an internal standard. [c]NMR yield determined by $^{1}$H-NMR using dimethyl maleate as an internal standard [d]Not determined due to overlapping signals.

**Table S5.** Initial solvent screening[a]

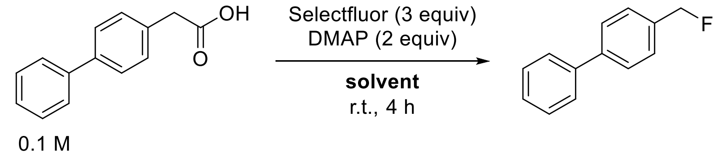


| Entry | Acetone:$H_2O$ | Conversion [%][b] | Yield [%][c] |
|---|---|---|---|
| 1 | 1:2 | 34 | 33 |
| **2** | **1:1** | **41** | **40** |
| 3 | 2:1 | 20 | 20 |
| 4 | 5:1 | 12 | 10 |

[a]Reaction conditions: 4-biphenylacetic acid (0.1 mmol), Selectfluor (0.3 mmol), DMAP (0.2 mmol), solvent (1 mL), r.t, 4 h. [b]Conversion of 4-biphenylacetic acid determined by $^{1}$H-NMR using dimethyl maleate as an internal standard. [a]NMR yield determined by $^{1}$H-NMR using dimethyl maleate as an internal standard

### 3.2 Temperature & Additive screening

**Table S6.** Temperature screening[a]

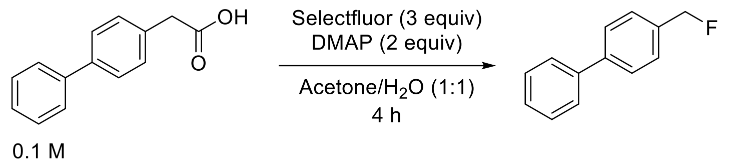


| Entry | Temperature [°C] | Conversion [%][b] | Yield [%][c] |
|---|---|---|---|
| 1 | r.t. | 41 | 40 |
| 2 | 50 | 47 | 44 |
| **3** | **70** | **62** | **50** |
| 4 | 90 | 43 | 16 |

[a]Reaction conditions: 4-biphenylacetic acid (0.1 mmol), Selectfluor (0.3 mmol), DMAP (0.2 mmol), acetone/$H_2O$ (1:1, 1 mL), 4 h. [b]Conversion of 4-biphenylacetic acid determined by $^1$H-NMR using dimethyl maleate as an internal standard. [a]NMR yield determined by $^1$H-NMR using dimethyl maleate as an internal standard

**Table S7.** Additive screening[a]

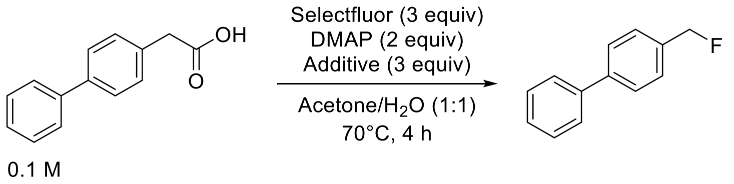


| Entry | Additive | Conversion [%][b] | Yield [%][c] |
|---|---|---|---|
| 1 | -- | 62 | 50 |
| 2 | $NaHCO_3$ | 33 | 22 |
| 3 | $Cs_2CO_3$ | 13 | traces |
| 4 | $Li_2CO_3$ | 23 | traces |
| 5 | $K_2CO_3$ | 17 | traces |
| 6 | KF | 84 | 56 |
| 7 | CsF | 84 | 62 |
| 8 | $K_3PO_4$ | 21 | traces |
| 9 | $K_2HPO_4$ | 42 | 34 |
| 10 | LiF | 70 | 56 |
| 11 | $MgF_2$ | 64 | 54 |
| 12 | $CaF_2$ | 61 | 52 |
| 13 | $(t\text{Bu})_4NF$ | 31 | 30 |
| **14** | **NaF** | **75** | **66** |
| 15 | $NH_4F$ | 76 | 60 |

[a]Reaction conditions: 4-biphenylacetic acid (0.1 mmol), Selectfluor (0.3 mmol), DMAP (0.2 mmol), acetone/$H_2O$ (1:1, 1 mL), 70°C, 4 h. [b]Conversion of 4-biphenylacetic acid determined by $^1$H-NMR using dimethyl maleate as an internal standard. [a]NMR yield determined by $^1$H-NMR using dimethyl maleate as an internal standard

### 3.3 Reaction time and concentration

**Table S8.** Optimization of reaction time and concentration[a]

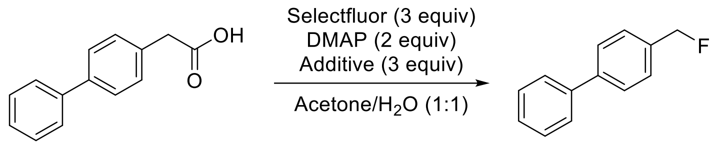


| Entry | Additive | Concentration [M] | Time | T [ºC] | Conversion [%][b] | Yield [%][c] |
|---|---|---|---|---|---|---|
| 1 | - | 0.1 | 4 h | r.t. | 41 | 40 |
| 2 | - | 0.2 | 4 h | r.t. | 48 | 48 |
| 3 | - | 0.2 | 4 h | 70 ºC | 80 | 70 |
| 4 | NaF | 0.2 | 4 h | 70 ºC | 88 | 86 |
| 5 | NaF | 0.2 | 1 h | 70 ºC | 90 | 86 |
| 6 | NaF | 0.2 | 30 min | 70 ºC | 86 | 85 |
| 7 | NaF | 0.2 | 10 min | 70 ºC | 85 | 82 |

[a]Reaction conditions: 4-biphenylacetic acid (0.1 mmol), Selectfluor (0.3 mmol), DMAP (0.2 mmol), acetone/$H_2O$ (1:1). [b]Conversion of 4-biphenylacetic acid determined by $^1$H-NMR using dimethyl maleate as an internal standard. [a]NMR yield determined by $^1$H-NMR using dimethyl maleate as an internal standard

### 3.4 Control studies

**Table S9.** Control studies[a]

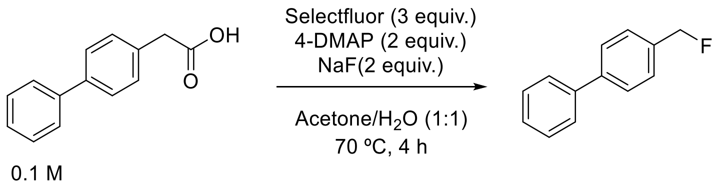


| Entry | Variation | Conversion [%][b] | Yield [%][c] |
|---|---|---|---|
| 1 | none | 88 | 86 |
| 2 | Selectfluor (2 equiv) | 64 | 42 |
| 3 | DMAP (1 equiv) | 95 | 86[c] |
| 4 | DMAP (10 mol%) | 48 | 30 |
| 5 | NaF (1 equiv) | 67 | 56 |
| 6 | Temperature (r.t.) | 40 | 32 |

[a]Reaction conditions: 4-biphenylacetic acid (0.1 mmol), Selectfluor, DMAP and NaF in acetone/water under $N_2$ atmosphere for 4 h. [b]Determined by $^1$H-NMR using dimethyl maleate as an internal standard. [c]The use of 1 equiv. of DMAP generated an unidentified side product.

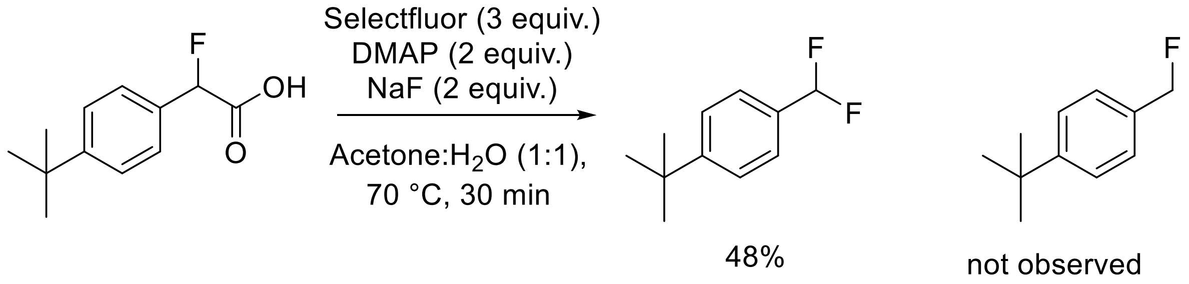


**Scheme S1.** Decarboxylative fluorination reaction of 2-(4-(tert-butyl)phenyl)-2-fluoroacetic acid

## 3.5 Scope

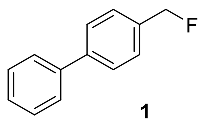

1

**General experimental procedure for the synthesis of 4-(fluoromethyl)-1,1'-biphenyl (1).** An oven dried vessel equipped with a stir bar was charged with substrate (424.5 mg, 2 mmol, 0.2 mM) and Selectfluor® (2.13 g, 6 mmol, 3 equiv.). Aceton:$H_2O$ (10 mL, 1:1) was added and the mixture was stirred and sonicated for 5 min. Subsequently, 4-dimethylaminopyridine (488.7 mg, 4 mmol, 2 equiv.) and NaF (167.9 mg, 4 mmol, 2 equiv.) were added. The vial was sealed and the reaction mixture was stirred and sonicated for 5 min. The mixture was degassed by bubbling Argon for 10 min. The reaction mixture was stirred in an oil bath at 70 °C for the respective time. The reaction mixture was quenched with HCl (1 M, 10 mL) and extracted with $CHCl_3$ (3 x 10 mL). The combined organic phases were washed with brine (10 mL), dried over $MgSO_4$ under concentrated under reduced pressure. Purification was performed by column chromatography using hexane. The title compound was isolated as a colorless solid (267.9 mg, 1.44 mmol, 72%). $^1$H NMR (400 MHz, CDCl3) δ. 7.71 – 7.66 (m, 4H), 7.54 – 7.50 (m, 4H), 7.47 – 7.41 (m, 1H). 7.47 – 7.41 (m, 1H), 5.48 (d, $J$ = 47.8 Hz, 1H). $^{13}$C NMR (400 MHz, $CDCl_3$) δ ppm 141.9, 140.7, 135.3 (d, $J$ = 17.2 Hz), 129.0, 128.3 (d, $J$ = 5.9 Hz), 127.7, 127.5, 127.3, 84.6 (d, $J$ = 166.2 Hz). $^{19}$F NMR (400 MHz, $CDCl_3$) δ ppm -206.2 (t, $J$ = 47.9 Hz). These data are in full agreement with those previously published in the literature.[1]

**General experimental procedure products that were not isolated due to their low boiling point.** An oven dried crimp top vial (19 x 100 mm) equipped with a stir bar was charged with substrate (0.1 mmol, 0.2 mM) and Selectfluor® (0.3 mmol, 3 equiv.). Aceton:$H_2O$ (2 mL, 1:1) was added and the mixture was stirred and sonicated for 5 min. Subsequently, 4-dimethylaminopyridine (0.2 mmol, 2 equiv.) and NaF (0.2 mmol, 2 equiv.) were added. The vial was sealed and the reaction mixture was stirred and sonicated for 5 min. The mixture was degassed by bubbling Argon for 10 min. The reaction mixture was stirred in an oil bath at 70 °C for the respective time. Upon completion, dimethyl maleate (12.4 µL, 0.1 mmol, 1 equiv.) was added. The reaction mixture was quenched with HCl (1 M, 1 mL) and extracted with $CDCl_3$ (1 mL). An aliquote (~200 µL) of the organic phase was subsequently subjected to $^1$H NMR analysis.

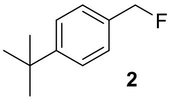

**2**

**1-(tert-butyl)-4-(fluoromethyl)benzene (2).** The compound was synthesized following the general procedure. An NMR yield of 84% was determined using dimethyl maleate as an internal standard. The product was not isolated due to its low boiling point.

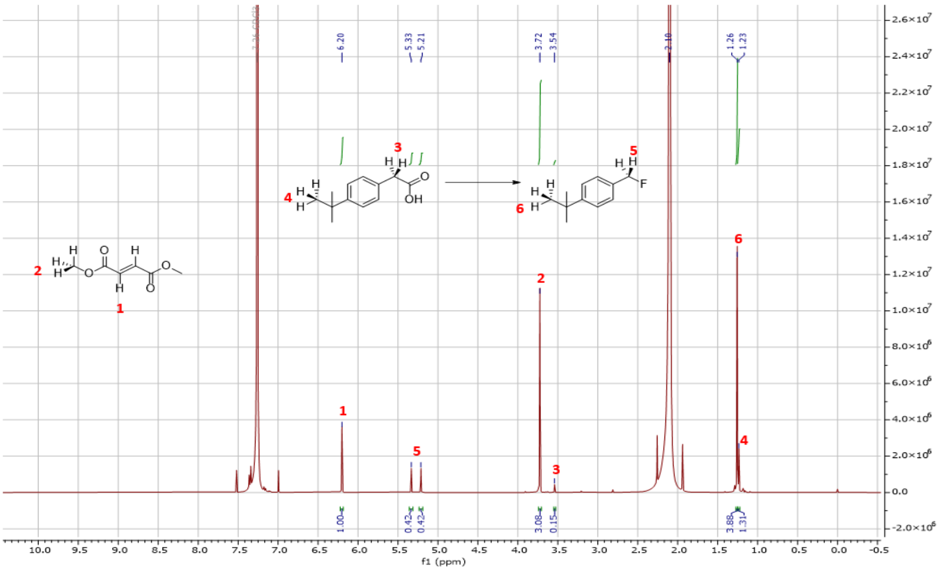


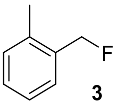

**3**

**1-(fluoromethyl)-2-methylbenzene (3).** The compound was synthesized following the general procedure. An NMR yield of 80% was determined using dimethyl maleate as an internal standard. The product was not isolated due to its low boiling point.

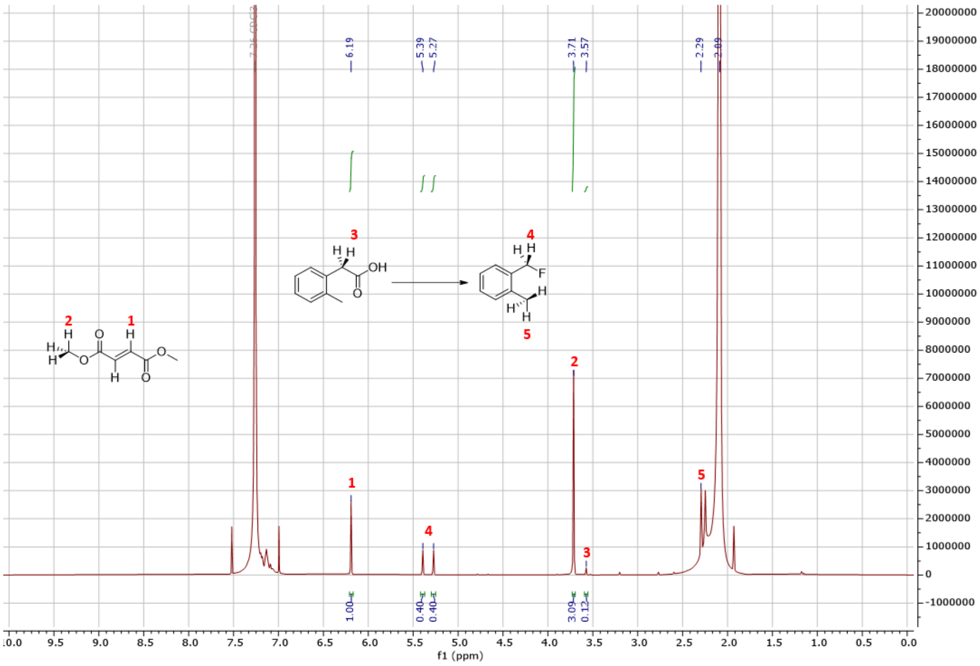

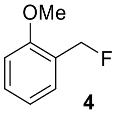


**4**

**2-(2-methoxyphenyl)acetic acid (4).** The compound was synthesized following the general procedure. An NMR yield of 56% was determined using dimethyl maleate as an internal standard. The product was not isolated due to its low boiling point.

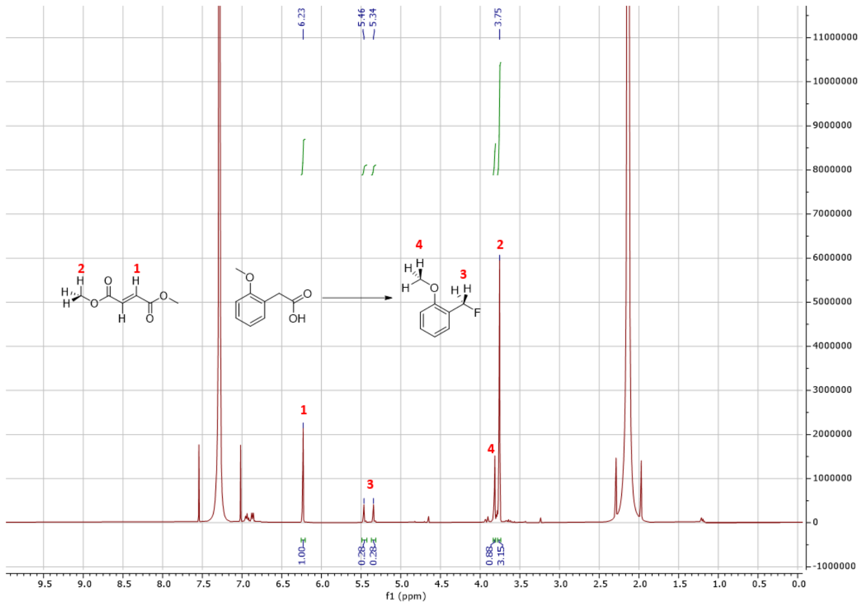


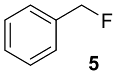


**5**

**(Fluoromethyl)benzene (5).** The compound was synthesized following the general procedure. An NMR yield of 60% was determined using dimethyl maleate as an internal standard. The product was not isolated due to its low boiling point.

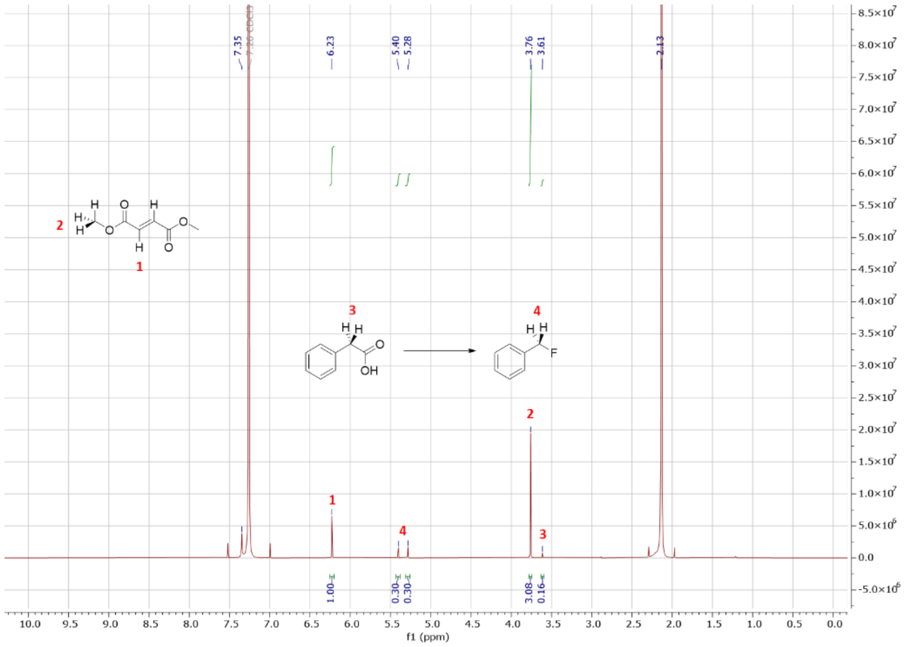

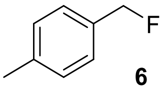

**6**

**1-(fluoromethyl)-4-methylbenzene (6).** The compound was synthesized following the general procedure. An NMR yield of 66% was determined using dimethyl maleate as an internal standard. The product was not isolated due to its low boiling point.

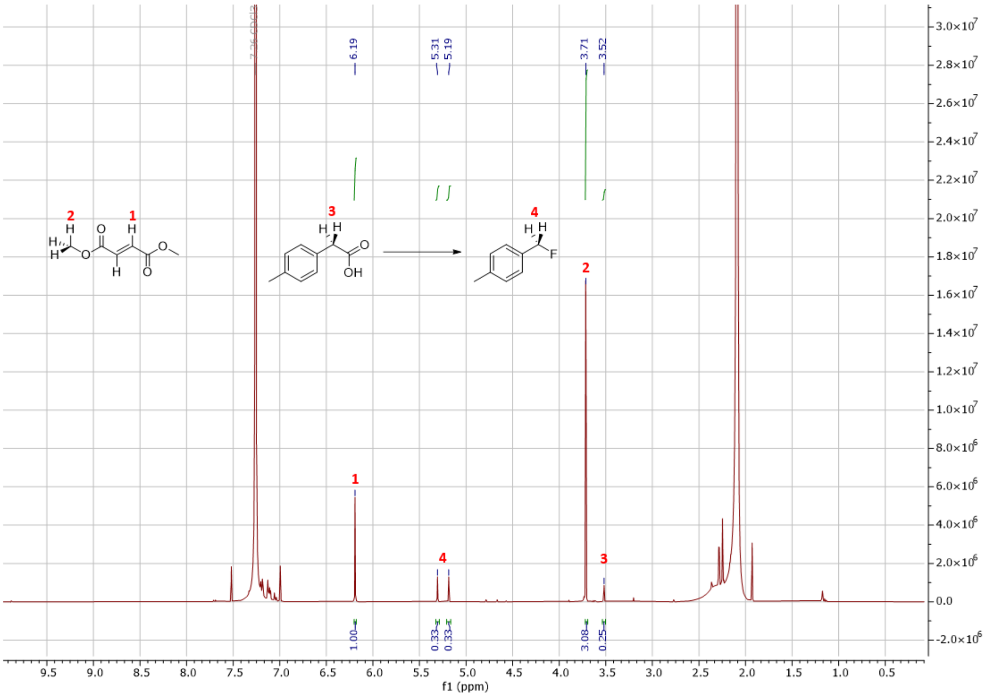


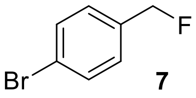

**7**

**1-bromo-4-(fluoromethyl)benzene (7).** The compound was synthesized following the general procedure. An NMR yield of 50% was determined using dimethyl maleate as an internal standard. The product was not isolated due to its low boiling point.

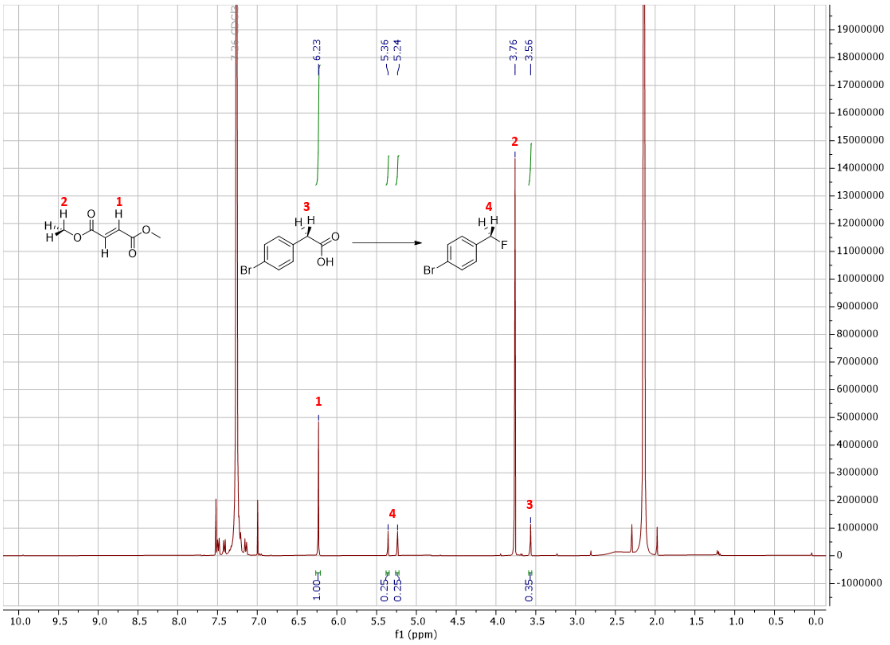

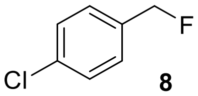

**1-chloro-4-(fluoromethyl)benzene (8).** The compound was synthesized following the general procedure. An NMR yield of 60% was determined using dimethyl maleate as an internal standard. The product was not isolated due to its low boiling point.

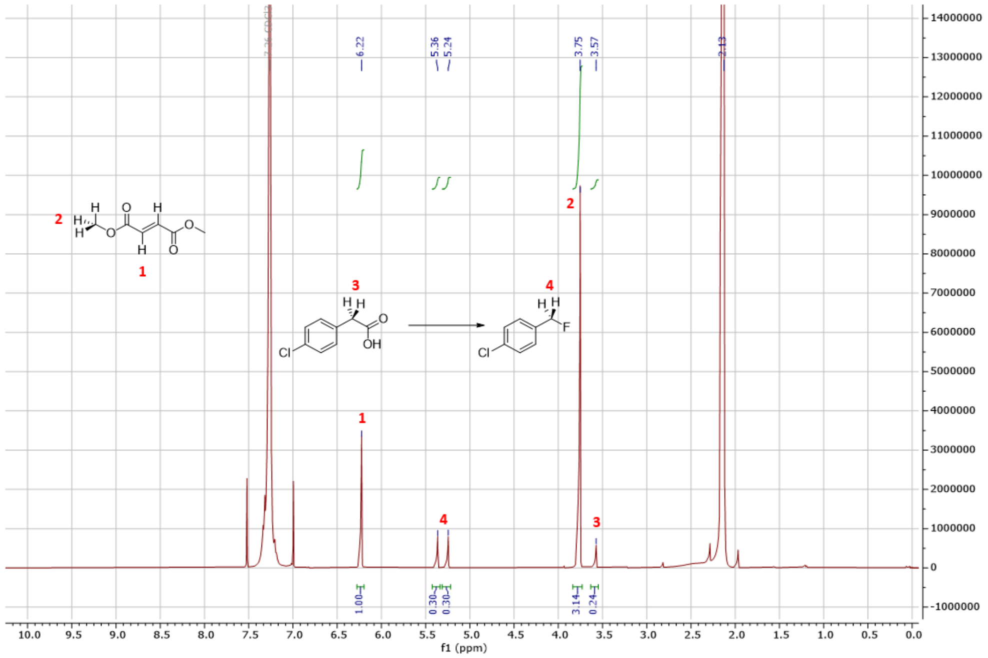


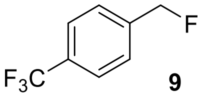

**1-(fluoromethyl)-4-(trifluoromethyl)benzene (9).** The compound was synthesized following the general procedure. An NMR yield of 20% was determined using dimethyl maleate as an internal standard. The product was not isolated due to its low boiling point.

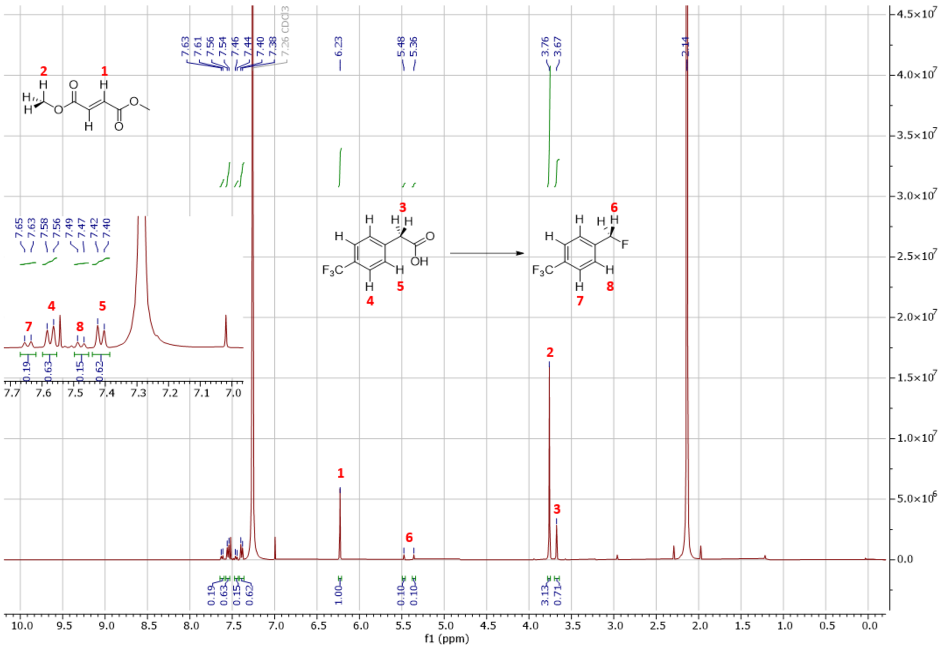

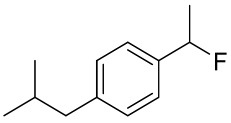

**40**

**1-(1-fluoroethyl)-4-isobutylbenzene (10).** The compound was synthesized following the general procedure. An NMR yield of 44% was determined using dimethyl maleate as an internal standard. The product was not isolated due to its low boiling point.

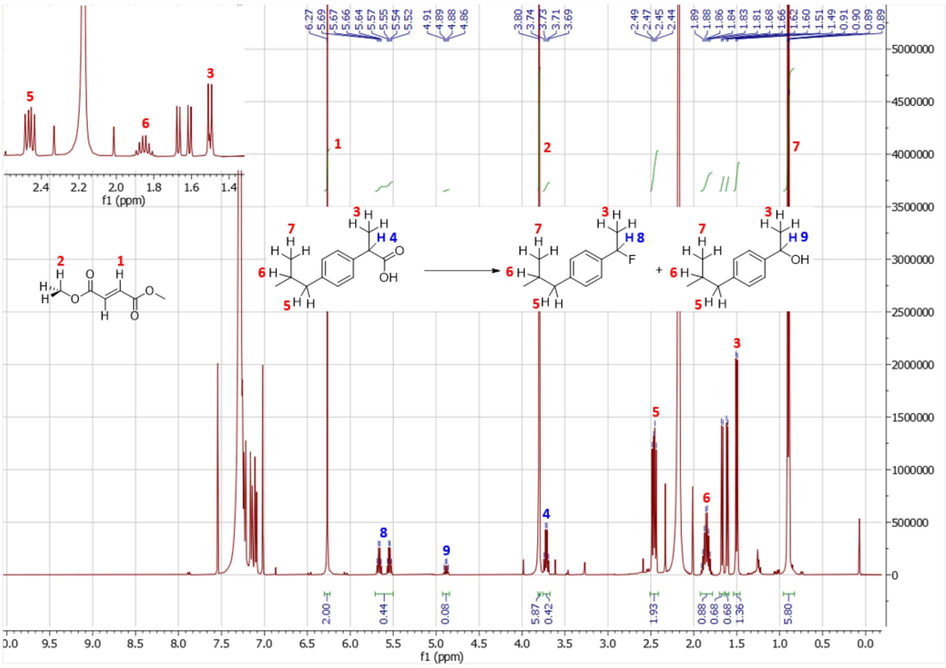

## 4. α-fluorination

**General experimental procedure for optimization experiments.** An oven-dried vessel (19 x 100 mm) equipped with a stir bar was charged with 4-fluorophenylacetic acid (or, if indicated another phenylacetic acid derivative), 4-dimethylaminopyridine and Selectfluor®. Solvent was added, the vessel was sealed with a septum and sonicated for 5 minutes. The mixture was stirred at room temperature for the respective reaction time. Upon completion, dimethyl maleate (1 equiv.) was added The reaction mixture was quenched with HCl (1 M, 1 mL) and extracted with $CDCl_3$ (1 mL). An aliquote (~200 µL) of the organic phase was subsequently subjected to $^1$H NMR analysis. NMR spectra were taken in $CDCl_3$ or MeOD-$d_3$. A representative $^1$H NMR spectrum for determining the yield is shown in Figure S2.

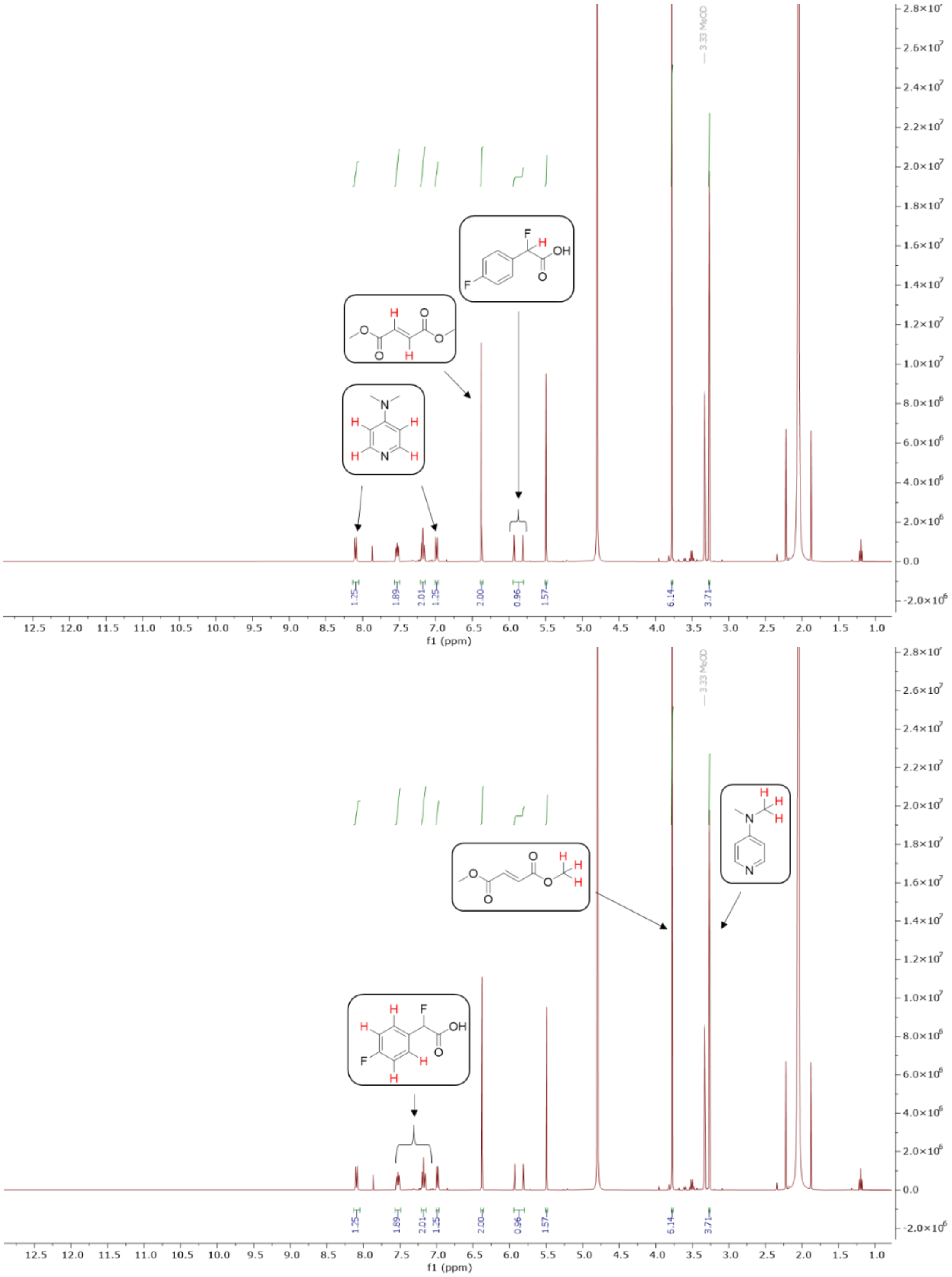


**Figure S2.** Example of a $^1$H-NMR spectrum (400 MHz) for determining NMR yields.

## 4.1 N–F Reagents

**Table S10.** Tests using different fluorinating agents[a]

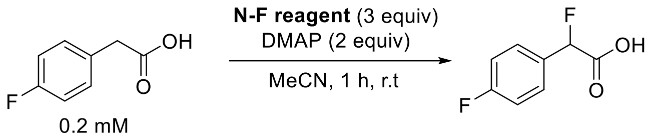


| Entry | Additive | Conversion [%][b] | Yield [%][c] |
|---|---|---|---|
| **1** | **Selectfluor** | **80** | **80** |
| 2 | NFSI | 40 | 40 |
| 3 | NFpy | - | - |

[a]Reaction conditions: 4-fluorophenylacetic acid (0.3 mmol), N-F reagent (0.9 mmol), DMAP (0.6 mmol) in MeCN (anhydrous, 1.5 mL), r.t for 1 h. [b]Conversion of 4-biphenylacetic acid determined by $^1$H-NMR using dimethyl maleate as an internal standard. [c]NMR yield determined by $^1$H-NMR using dimethyl maleate as an internal standard

## 4.2 Solvent screening

**Table S11.** Solvent screening[a]

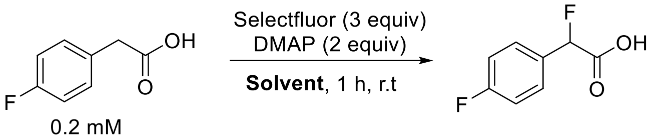


| Entry | Additive | Conversion [%][b] | Yield [%][c] |
|---|---|---|---|
| **1** | **MeCN** | **80** | **80** |
| 2 | Acetone | 56 | 56 |
| 3 | DMF | 73 | 73 |
| 4 | MeOH | 75 | n.d |
| 5 | THF | 0 | n.d |
| 6 | $CHCl_3$ | 0 | n.d |
| 7 | DCM | 0 | n.d |

[a]Reaction conditions: 4-fluorophenylacetic acid (0.3 mmol), Selectfluor (0.9 mmol), DMAP (0.6 mmol) in solvent (anhydrous, 1.5 mL), r.t for 1 h. [b]Conversion of 4-biphenylacetic acid determined by $^1$H-NMR using dimethyl maleate as an internal standard. [c]NMR yield determined by $^1$H-NMR using dimethyl maleate as an internal standard

## 4.3 Optimization of the equivalents of Selectfluor and DMAP

**Table S12.** Optimization experiments of the equivalents of Selectfluor and DMAP[a]

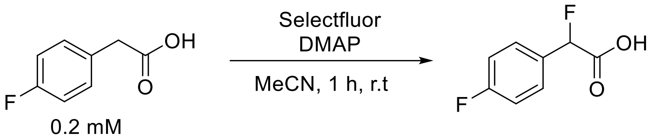


| Entry | Selectfluor (equiv) | DMAP (equiv) | Conversion [%][b] | Yield [%][c] |
|---|---|---|---|---|
| 1 | 3 | 2 | 80 | 80 |
| 2 | 3 | 1 | 38 | 38 |
| 3 | 3 | 0.5 | 3 | 3 |
| 4 | 3 | 0.1 | 2 | 2 |
| 5 | 3 | 2 | 80 | 80 |
| 6 | 2 | 2 | 89 | 89 |
| **7** | **1.2** | **2** | **97** | **97** |

[a]Reaction conditions: 4-fluorophenylacetic acid (0.3 mmol), Selectfluor and DMAP in MeCN (anhydrous, 1.5 mL), r.t for 1 h. [b]Conversion of 4-biphenylacetic acid determined by $^{1}$H-NMR using dimethyl maleate as an internal standard. [c]NMR yield determined by $^{1}$H-NMR using dimethyl maleate as an internal standard

## 4.4 Screening of different activators

**Table S13**. Activator screening[a]

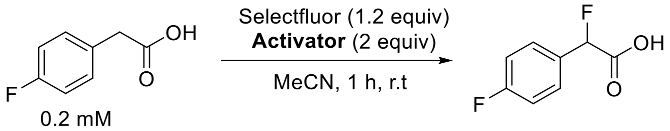


| Entry | Activator | Conversion [%][b] | Yield [%][c] |
|---|---|---|---|
| **1** | **DMAP** | **97** | **97** |
| 2 | *N*,*N*-Dimethylanilin | 0 | n.d |
| 3 | Pyridine | 9 | 9 |
| 4 | 4-Methoxypyridine | 66 | 66 |
| 5 | 4-Aminopyridine | 82 | 82 |
| 6 | 2-Phenylpyridine | 0 | n.d |
| 7 | Quinoline | 0 | n.d |
| 8 | 3-Hydroxypyridine | 0 | n.d |
| 9 | 6-Cyanopyridine | 0 | n.d |
| 10 | 2-Chloropyridine | 0 | n.d |
| 11 | 2,6-Lutidine | 52 | n.d |
| 12 | DABCO | 0 | n.d |

[a]Reaction conditions: 4-fluorophenylacetic acid (0.3 mmol), Activator (0.36 mmol), DMAP (0.6 mmol) in solvent (anhydrous, 1.5 mL), r.t for 1 h. [b]Conversion of 4-biphenylacetic acid determined by $^{1}$H-NMR using dimethyl maleate as an internal standard. [c]NMR yield determined by $^{1}$H-NMR using dimethyl maleate as an internal standard

## 4.5 Concentration and reaction time studies

**Table S14.** Concentration/time studies[a]

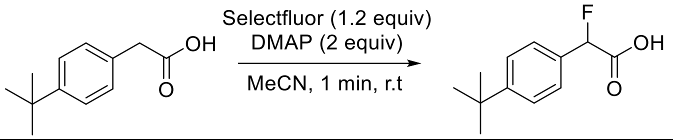


| Entry | Concentration [mM] | Conversion [%][b] | Yield [%][c] | |
|---|---|---|---|---|
| | | | 1 min | 5 min |
| 1 | 0.2 | 92 | 92 | 92 |
| 2 | 0.1 | 85 | 85 | 91 |
| 3 | 0.05 | 58 | 58 | 72 |
| 4 | 0.2[d] | 79 | 79 | - |

[a]Reaction conditions: 4-*tert*-Butylphenylacetic acid (0.3 mmol), Selectfluor (0.36 mmol), DMAP (0.6 mmol) in MeCN (anhydrous), r.t for 1 min [b]Conversion of 4-biphenylacetic acid determined by $^1$H-NMR using dimethyl maleate as an internal standard. [c]NMR yield determined by $^1$H-NMR using dimethyl maleate as an internal standard. [d]0°C

**Table S15.** One minute experiments using different substrates[a]

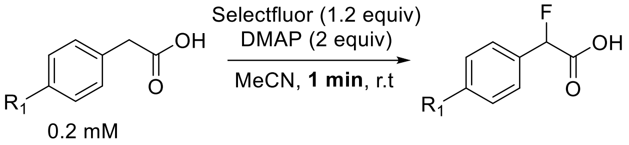


| Entry | Substrate | Conversion [%][b] | Yield [%][c] |
|---|---|---|---|
| 1 | 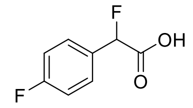 | 96 | 96 |
| 2 | 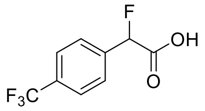 | 89 | 89 |
| 3 | 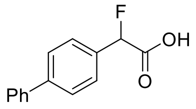 | 93 | 93 |
| 4 | 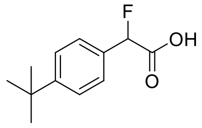 | 92 | 92 |
| 5 | 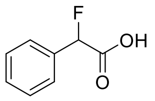 | 99 | 99 |

[a]Reaction conditions: Phenylacetic acid derivative (0.3 mmol), Selectfluor (0.36 mmol), DMAP (0.6 mmol) in MeCN (anhydrous, 1.5 mL), r.t for 1 min. min [b]Conversion of 4-biphenylacetic acid determined by $^1$H-NMR using dimethyl maleate as an internal standard. [c]NMR yield determined by $^1$H-NMR using dimethyl maleate as an internal standard.

## 4.6 Mechanistic investigations

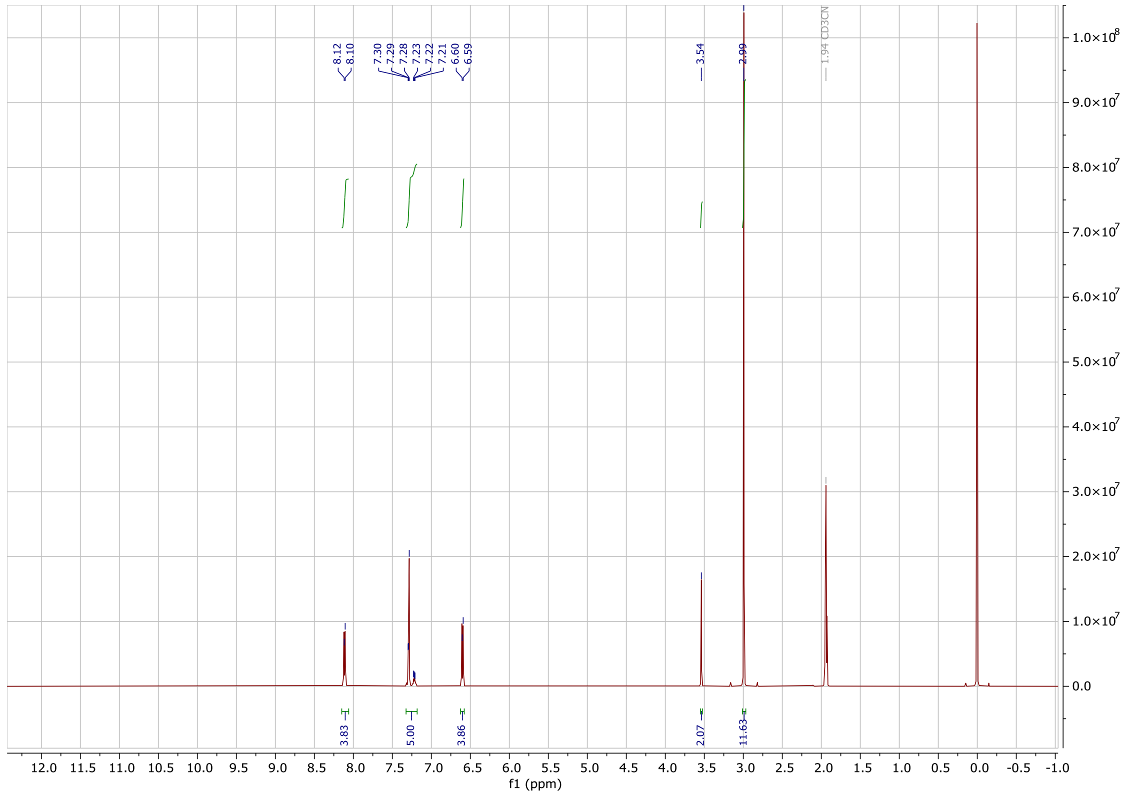


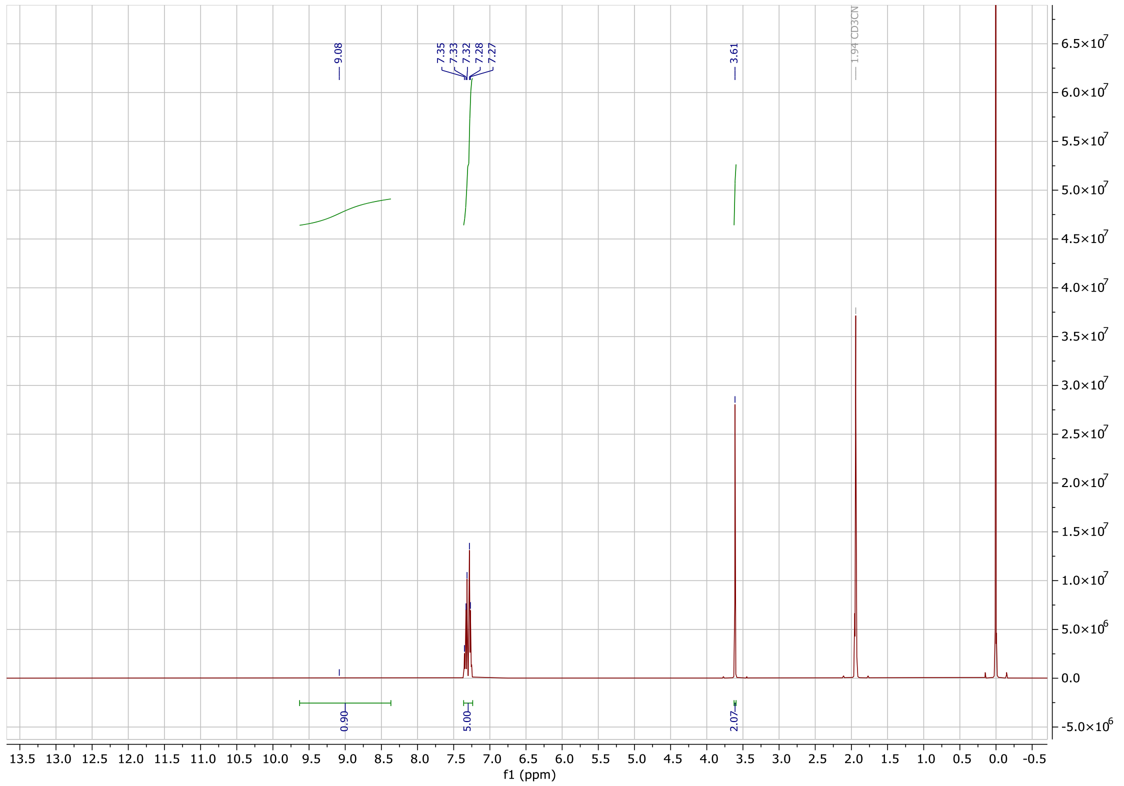


**Figure S3.** $^{1}H$ spectrum (400 MHz) of phenylacetic acid (bottom), and a mixture of phenylacetic acid and DMAP (2 equiv.) in $CD_3CN$.

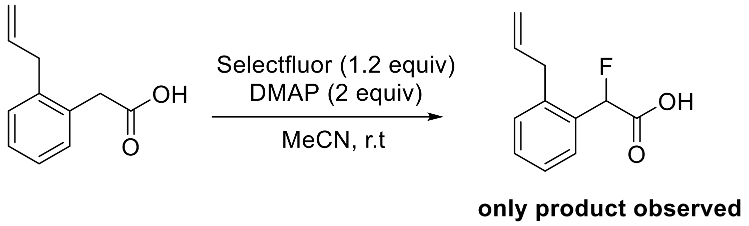


**Scheme S2.** Radical clock experiment. The substrate was synthesized according to literature.[2]

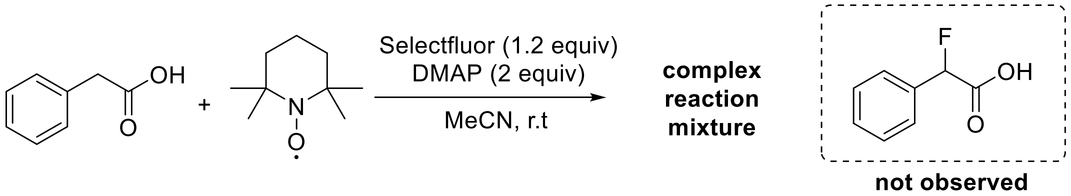


**Scheme S3.** Experiment in presence of TEMPO.

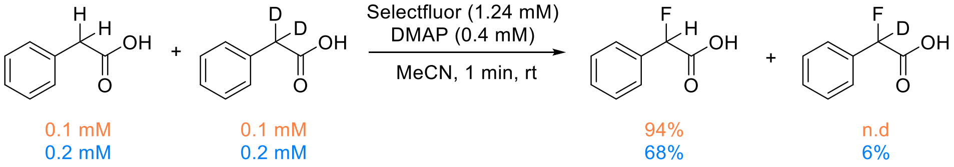


**Scheme S4.** Competition experiment between phenylacetic acid and deuterium-labelled phenylacetic acid. The labelled compound was synthesized according to a literature procedure.[3]

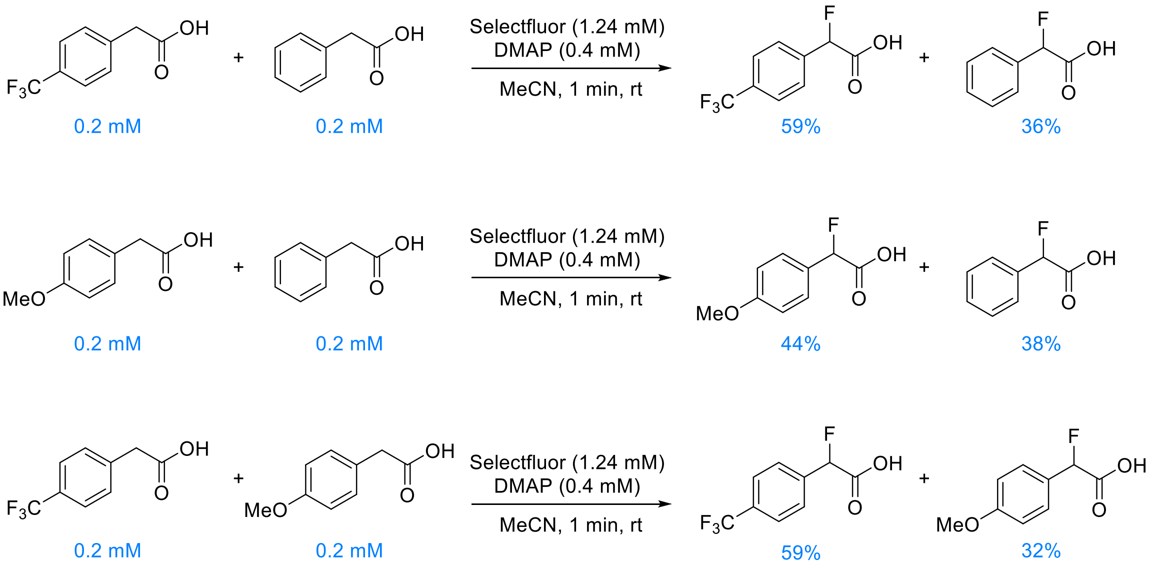


**Scheme S5.** Competitive experiments between phenylacetic acids derivatives.

## 4.7 Delayed addition experiments

**Table S16.** Delayed addition experiments[a]

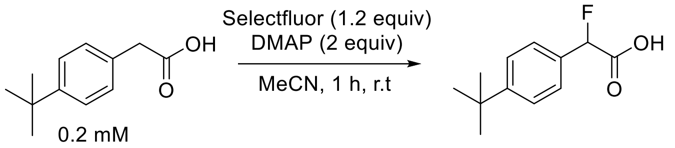


| Entry | Compound added after 30 min | Conversion [%][b] | Yield [%][c] |
|---|---|---|---|
| **1** | **Substrate** | **n.d** | **n.d** |
| 2 | Selectfluor | 100 | 100 |
| 3 | DMAP | 88 | 88 |

[a]Reaction conditions: 4-*tert*-Butylphenylacetic acid (0.3 mmol), Selectfluor (0.36 mmol), DMAP (0.6 mmol) in MeCN (anhydrous, 1.5 mL), r.t. for 1 h. [b]Conversion of 4-biphenylacetic acid determined by $^{1}$H-NMR using dimethyl maleate as an internal standard. [c]NMR yield determined by $^{1}$H-NMR using dimethyl maleate as an internal standard.

## 4.8 Reaction monitoring using a ReactIR

To maintain a constant operating temperature, the ReactIR console was filled with liquid nitrogen every 12 hours. Prior to each experiment, a background spectrum was recorded before attaching the reaction vessel. The raw ReactIR data was treated with a negative second derivative function, to aid in separation of peaks. The reference spectrum of the solvent was subtracted. The product peak appeared at ~1648 $cm^{-1}$ (C-H bending, aromatic), Selectfluor was detected at 1005 $cm^{-1}$ (*N*-F bond) and the consumption of DMAP was observed at 1608 $cm^{-1}$ (C=C semicircle stretch).[4-5] Data obtained from the iCiR was processed using OriginPro2021.

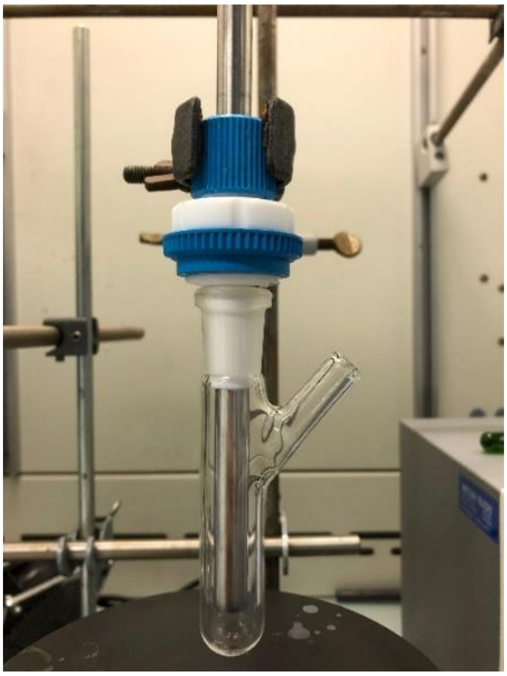

**Figure S4.** ReactIR setup shown with reaction vessel attached to the probe. The custom-made sidearm allows the delayed addition of reagents, degassing or sampling.

**Experimental procedure for reaction monitoring.** A custom-made reaction vessel (19 x 100 mm) with a sidearm (Figure S4) was equipped with a stir bar and charged with 4-tert-butylphenylacetic acid (115. 35 mg, 0.2 mM, 0.6 mmol) and 4-dimethylaminopyridine (146.6 mg, 1.2 mmol, 2 equiv.). MeCN (3 mL) was added, the vessel was sealed and sonicated for 5 minutes. The reaction vessel was attached to a PTFE adapter affixed to the ReactIRs probe (Figure S3). The reaction mixture was stirred for 30 min while collecting data (Figure S4). After this period, Selectfluor (255.06 mg, 0.72 mmol, 1.2 equiv.) was added and the mixture was stirred for 1 h at r.t.

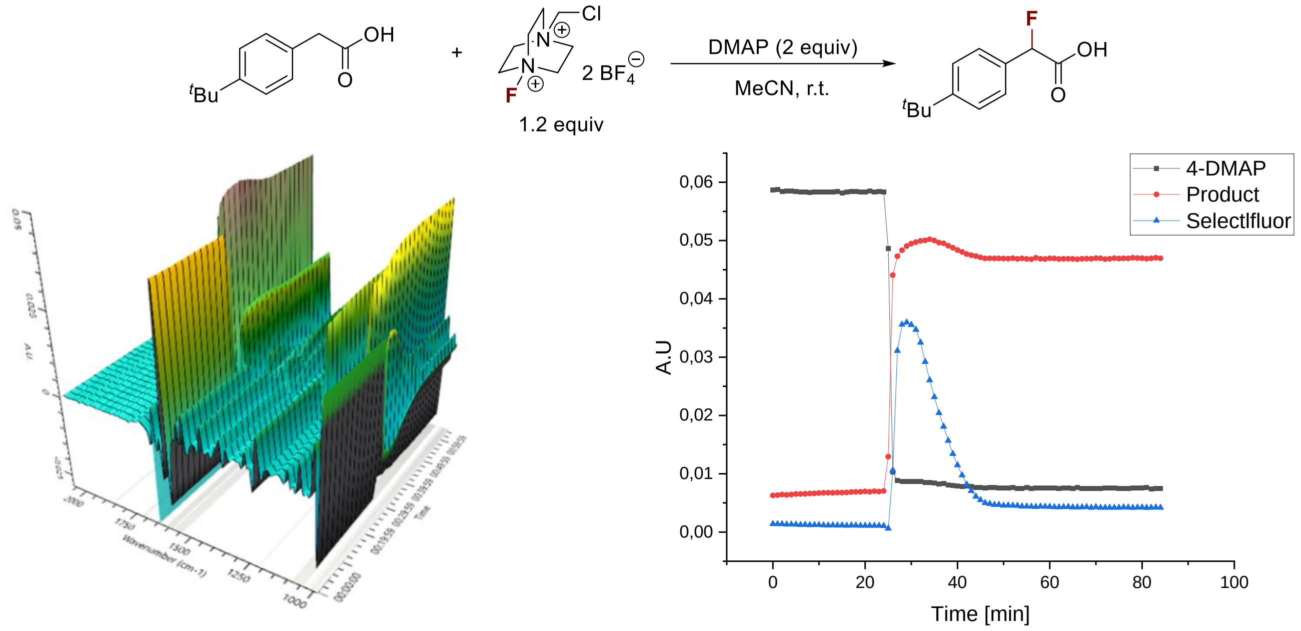


**Figure S5.** Reaction monitoring of the fluorination of 2-(4-(*tert*-butyl)phenyl)acetic acid using ReactIR. Raw data output (left) and processed reaction profile ((right).

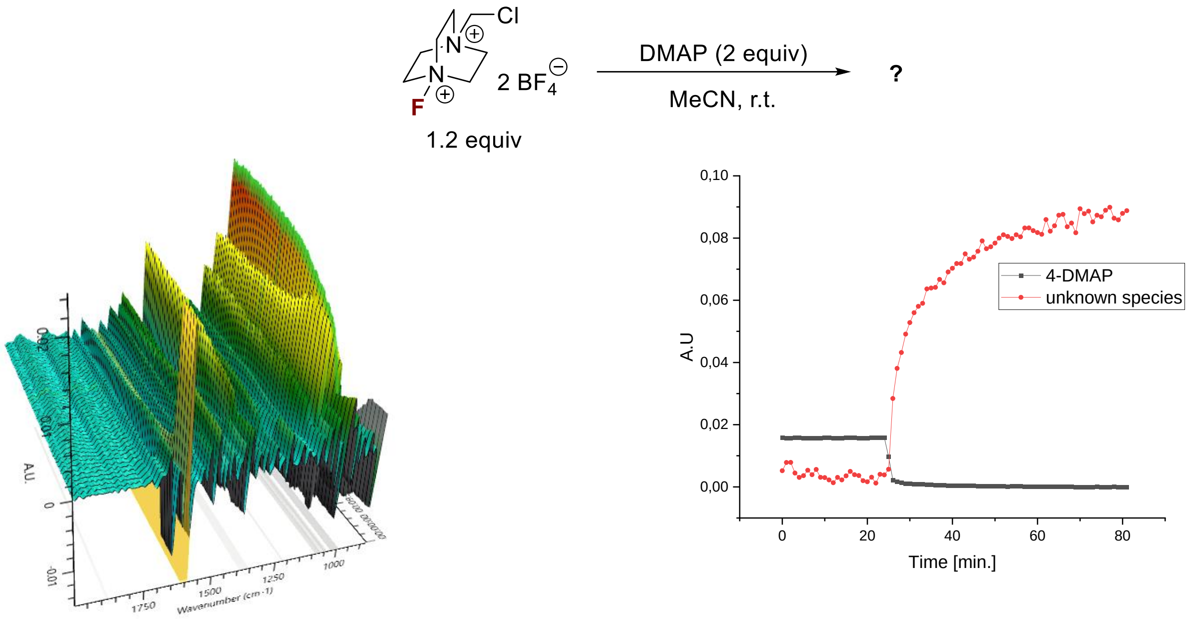


**Figure S6.** Monitoring of the reaction of DMAP and Selectfluor in absence of starting material using ReactIR. Raw data output (left) and processed reaction profile ((right).

## 4.9 NMR experiments

Initial NMR experiments with SelectFluor and DMAP in absence of starting material indicated that HF (signs of glass etching of the NMR tubes). To prevent glass etching and consumption of HF by this process, we used poly propylene micro reaction caps and Teflon NMR inserts for further measurements.
DMAP forms an adduct with HF (Figure S7 and S8). The triplet signal at 11.42 ppm shows a long-range coupling to the pyridinium nitrogen in the $^{1}H$-$^{15}N$-HMBC spectrum (Figure S8). Similar pyridinium polyhydrogen fluorides are known, such as Olahs reagent.[6]

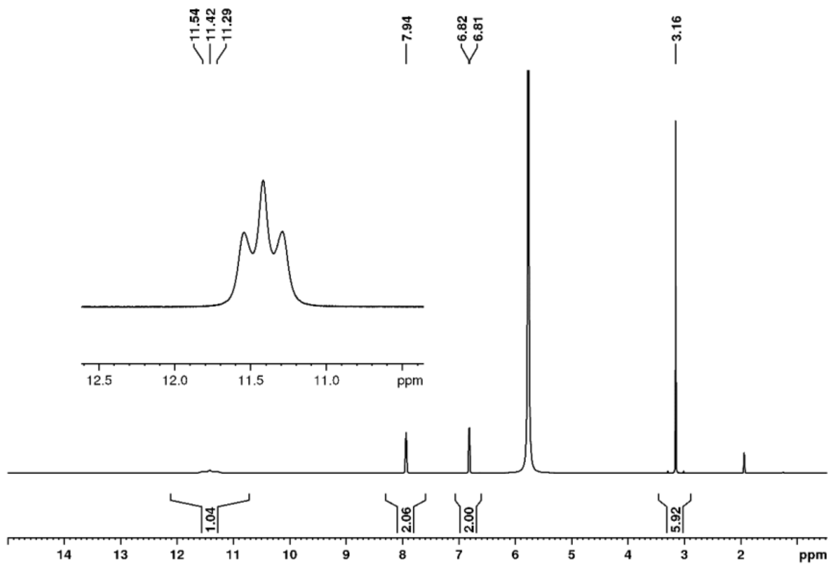


**Figure S7.** $^{1}H$ NMR spectrum (500 MHz) of DMAP in presence of HF in $CD_3CN$. HF (48%, 0.038 mL, 1.06 mmol) was added to DMAP (6.1 mg, 0.05 mmol) in $CD_3CN$ (0.35 mL). DMAP forms an adduct with HF. The triplet at 11.4 ppm originates from $(F\text{-}H\text{-}F)^-$ coordinated to the pyridine nitrogen of DMAP. The two $^{19}F$ coupling partners lead to a triplet splitting of the $^{1}H$ signal.

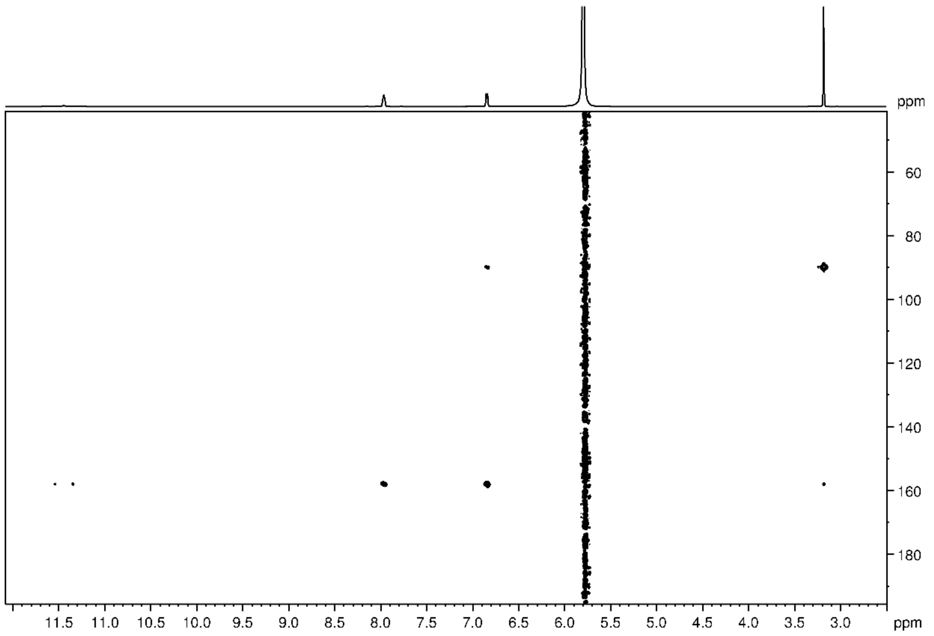


**Figure S8.** $^{15}N$-$^{1}H$-HMBC spectrum of DMAP in presence of HF in $CDCN_3$. HF (48%, 0.038 mL, 1.06 mmol) was added to DMAP (6.1 mg, 0.05 mmol) in $CD_3CN$ (0.35 mL).

In the $^{1}H$ NMR spectrum of a mixture of SelectFluor with DMAP, we observed the same triplet signal indicative of a pyridinium HF adduct (Figure S9 and S10).

The superposition of the $^{1}H$-$^{15}N$-HMBC spectra of the DMAP/HF mixture with the reaction mixture of SelectFluor/DMAP shows that, also when using SelectFluor, the pyridinium nitrogen resonance shifts to ca. 160 ppm indicative of the formation of the (F-H-F)-adduct. In this case, however, the (F-H-F)-Triplett is too broad such that the long-range correlation signal could not be detected.

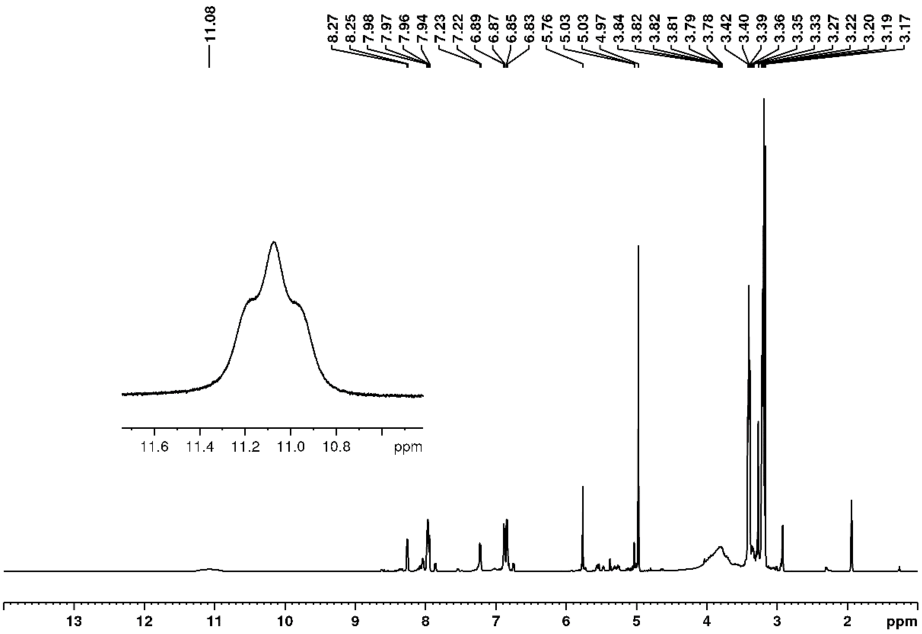


**Figure S9**: $^{1}H$ NMR spectrum (500 MHz) of a reaction mixture of Selectfluor (17.7 mg, 0.05 mmol) and DMAP (6.1 mg, 0.05 mmol) in $CD_3CN$ (0.35 mL) after a reaction time of three days.

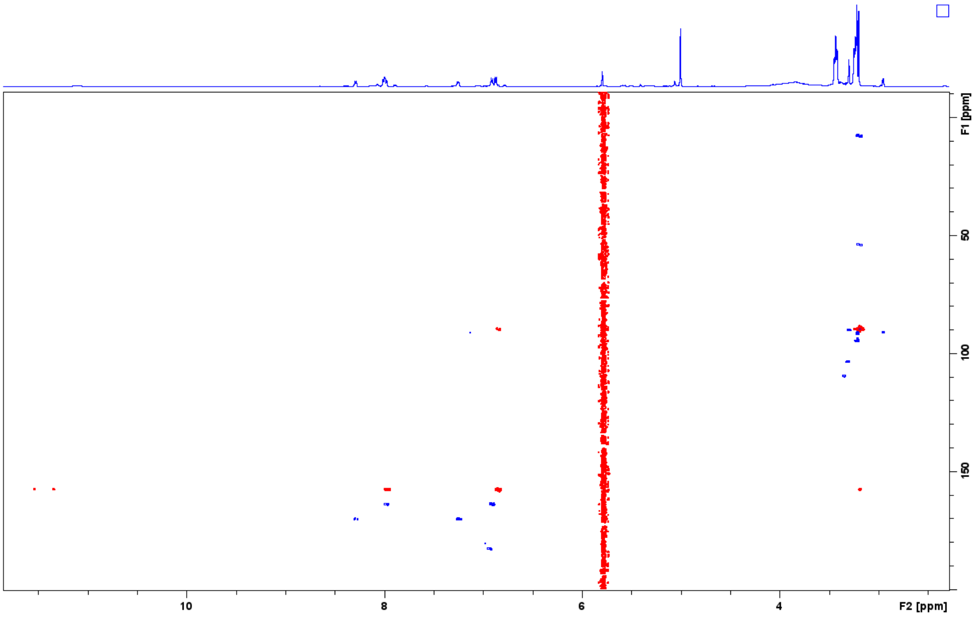


**Figure S10**. Superposition of $^{1}H$-$^{15}N$-HMBC spectra. Red: HF (48%, 0.038 mL, 1.06 mmol) was added to DMAP (6.1 mg, 0.05 mmol) in $CD_3CN$ (0.35 mL). Blue: Selectfluor (17.7 mg, 0.05 mmol) and DMAP (6.1 mg, 0.05 mmol) in $CD_3CN$ (0.35 mL) after a reaction time of 3 days.

$^{19}$F NMR measurements show that HF is liberated very early after mixing the reactants ($\delta_{19F}$ = -166 ppm, see Figure S10). Right after mixing, additional, transient signals ($\delta_{19F}$ = -19 and -14 ppm, respectively) are observed in the $^{19}$F NMR spectrum. These might originate from *N*-fluorinated species. However, we have not been able to prove this beyond doubt. After a reaction time of (here) several days, these transient signals have disappeared, and the $^{19}$F signal of HF has shifted slightly to ($\delta_{19F}$ = -177 ppm, Figure S11). In this regard, it has to be noted that the $^{19}$F chemical shift of HF depends strongly on its concentration, the presence of $H^+$ and other coordinating agents.

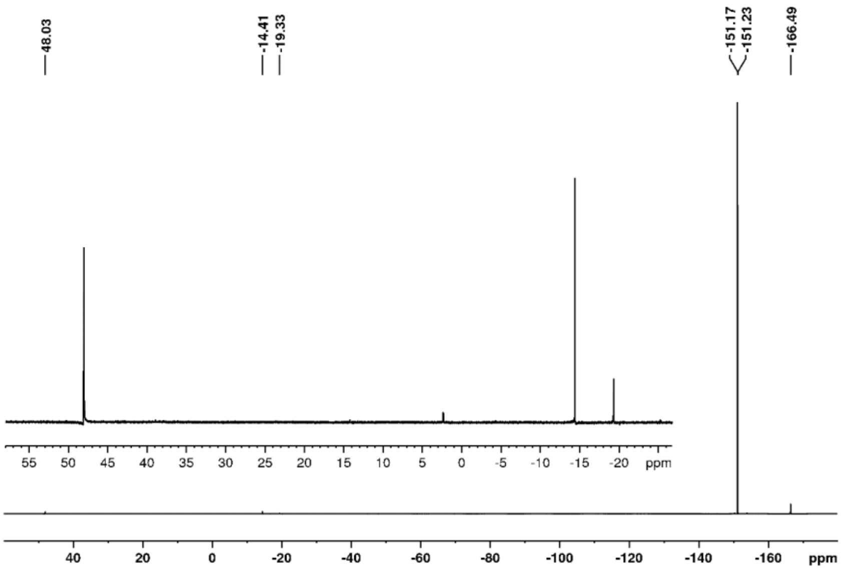


**Figure S11.** $^{19}$F NMR (471 MHz) spectrum directly after mixing Selectfluor (17.7 mg, 0.05 mmol) and DMAP (6.1 mg, 0.05 mmol) in $CD_3CN$ (0.35 mL). The large signal at ca. -150 ppm originates from $BF_4^-$.

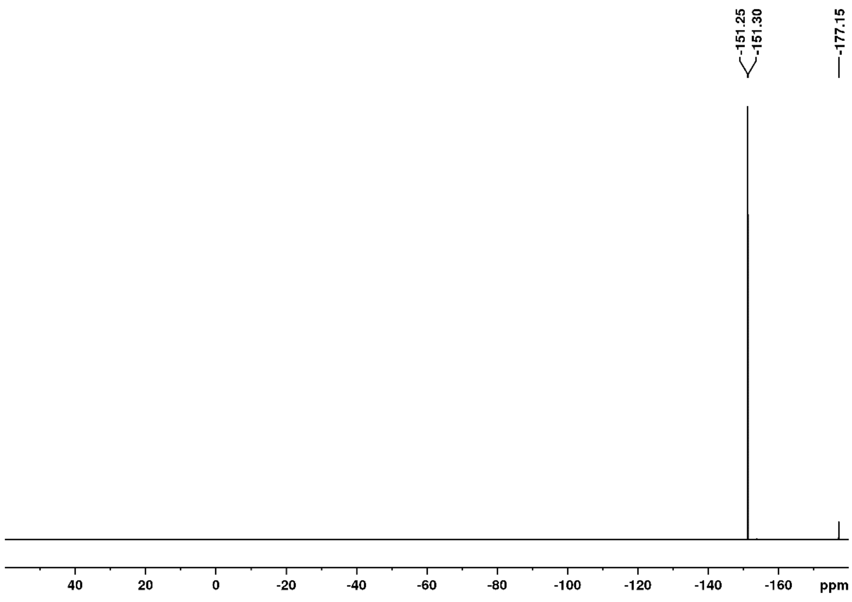


**Figure S12.** $^{19}$F NMR spectrum (471 MHz) of a reaction mixture of Selectfluor (17.7 mg, 0.05 mmol) and DMAP (6.1 mg, 0.05 mmol) in $CD_3CN$ (0.35 mL) after three days. Transient $^{19}$F signals, observed right after mixing, have disappeared. Only the large signals of $BF_4^-$ at ca. -150 ppm and HF at -177 ppm are visible.

Neutralization with NaOH converts HF into $F^-$. Applied to the sample shown in Figure S11, still only two $^{19}F$ signals are observed, confirming the presence of $BF_4^-$ and $F^-$ (Figure S12). The amount of HF and $F^-$, resp., increases with time. Calibrating the integral of the $BF_4^-$-signal to 100%, the integrals of the HF signals change from 20 % (after mixing), 26 % (after 4h) to 28% and reach 25% for the quenched $F^-$ signal.

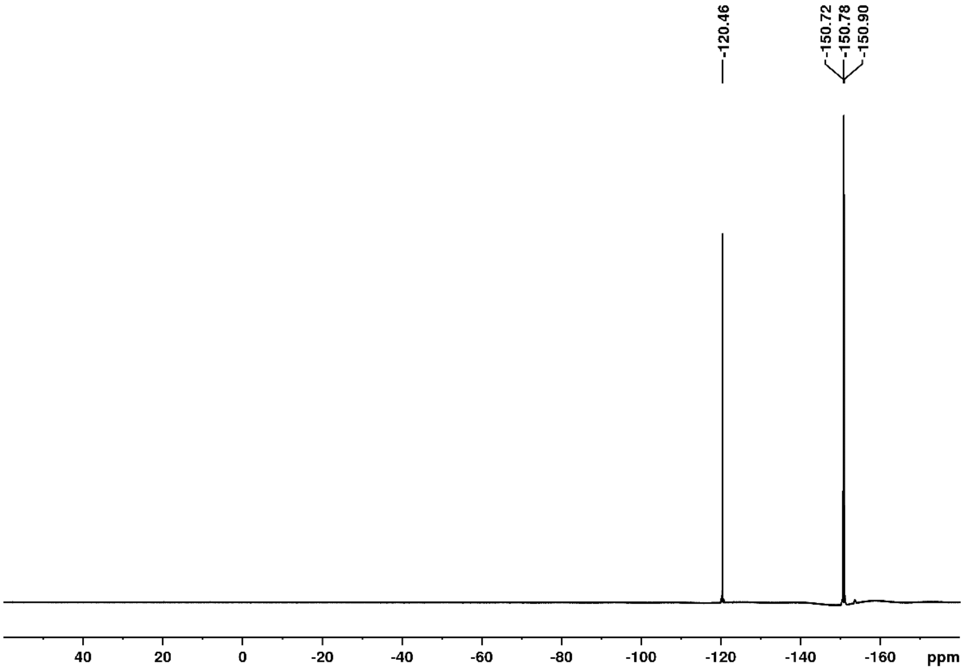


**Figure S13:** $^{19}F$ NMR spectrum (471 MHz) of a reaction mixture of Selectfluor (17.7 mg, 0.05 mmol) and DMAP (6.1 mg, 0.05 mmol) in $CD_3CN$ (0.35 mL) after 4 days after adding 0.2 ml 1M NaOH (0.2 mmol) for neutralization. The large signal at -150 ppm originates from of $BF_4^-$and the signal at -120 ppm originates from $F^-$.

## 5 Scope

**General experimental procedure.** An oven-dried vessel (19 x 100 mm) equipped with a stir bar was charged with substrate (1.8 mmol, 1 equiv, 0.2 mM), 4-dimethylaminopyridine (3.6 mmol, 2 equiv.) and Selectfluor. MeCN (9 mL) was added, the vessel was sealed stirred at room temperature for one hour. HCl (10 mL, 1 M) was added and the mixture was extracted with diethylether (3 x 10 mL). The combined organic phases were dried over $Na_2SO_4$, filtered and concentrated. The product was purified by column chromatography ($SiO_2$, Hexane/EtOAc or Hexane/MeOH) on a Grace™ Reveleris™ system.

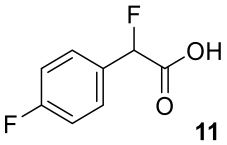

**11**

**2-Fluoro-2-(4-fluorophenyl)acetic acid (11).** From 2-(4-fluorophenyl)acetic acid (277.45 mg, 1.8 mmol, 0.2 mM) using 4-(dimethylamino)-pyridine (439.8 mg, 3.6 mmol, 2 equiv.) and 1-chloromethyl-4-fluoro-1,4-diazoniabicyclo[2.2.2]octanebis(tetrafluoroborate) (765.2 mg, 2.16 mmol, 1.2 equiv.) in 9 mL MeCN. The crude mixture was purified over silica with flash chromatography, using an eluent gradient of 5-20% EA in hexane to afford the title compound as a white powder (221.8 mg, 1.3 mmol, 72%).

**$^{1}$H NMR** (400 MHz, $CDCl_3$) δ 8.84 (s, 1H), 7.49–7.46 (m, 2H), 7.15 – 7.07 (m, 2H), 5.81 (d, *J* = 47.2 Hz, 1H) ppm. **$^{13}$C NMR** (101 MHz, $CDCl_3$) δ 173.8 (d, *J* = 28.2 Hz), 163.7 (dd, *J* = 249.5, 2.2 Hz), 129.4 (dd, *J* = 21.1, 3.4 Hz), 129.0 – 128.8 (m), 116.2 (d, *J* = 22.1 Hz), 88.3 (d, *J* = 187.3 Hz) **$^{19}$F NMR** (376 MHz, $CDCl_3$) δ -110.59– -110.67 (m) -179.54 (d, *J* = 46.8 Hz) ppm.

These data are in full agreement with those previously published in the literature.[7]

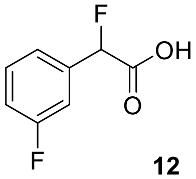

**12**

**2-fluoro-2-(3-fluorophenyl)acetic acid (12).** From 2-(3-fluorophenyl)acetic acid (277.45 mg, 1.8 mmol, 0.2 mM) using 4-(dimethylamino)-pyridine (439.8 mg, 3.6 mmol, 2 equiv.) and 1-chloromethyl-4-fluoro-1,4-diazoniabicyclo[2.2.2]octanebis(tetrafluoroborate) (765.2 mg, 2.16 mmol, 1.2 equiv.) in 9 mL MeCN. The crude mixture was purified over silica with flash chromatography, using an eluent gradient of 0–10% EA in hexane to afford the title compound as a yellowish powder (160.0 mg, 0.93 mmol, 52%).

$^{1}$H NMR (400 MHz, $CDCl_3$) δ 11.56 (s, 1H), 7.40– 7.35 (m, 1H), 7.26– 7.24 (m, 1H), 7.19 (d, *J* = 9.2 Hz, 1H), 7.12– 7.07 (m, 1H), 5.80 (d, *J* = 47.1 Hz, 1H) ppm. $^{13}$C NMR (101 MHz, $CDCl_3$) δ 174.3 (d, *J* = 27.5 Hz), 162.9 (d, *J* = 247.9 Hz), 135.6 (dd, *J* = 27.9, 8.9 Hz), 130.8 (d, *J* = 8.2 Hz), 122.3 (dd, *J* = 6.6, 3.1 Hz), 117.1 (dd, *J* = 21.0, 1.8 Hz), 113.8 (dd, *J* = 23.2, 6.9 Hz), 88.1 (dd, *J* = 188.6, 2.2 Hz) ppm. $^{19}$F NMR (376 MHz, $CDCl_3$) δ -111.37, -183.17 (d, *J* = 47.3 Hz) ppm. HRMS (ESI) *m/z* calculated [M+2Na-H] 217.0011, found, 217.0012.

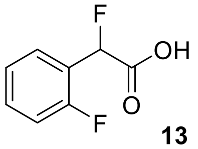


**13**

**2-Fluoro-2-(2-fluorophenyl)acetic acid (13).** From 2-(2-fluorophenyl)acetic acid (277.45 mg, 1.8 mmol, 0.2 mM) using 4-(dimethylamino)-pyridine (439.8 mg, 3.6 mmol, 2 equiv.) and 1-chloromethyl-4-fluoro-1,4-diazoniabicyclo[2.2.2]octanebis(tetrafluoroborate) (765.2 mg, 2.16 mmol, 1.2 equiv.) in 9 mL MeCN. The crude mixture was purified over silica with flash chromatography, using an eluent gradient of 0–10% EA in hexane to afford the title compound as a yellowish powder (163.1 mg, 0.95 mmol, 53%).

$^{1}$H NMR (400 MHz, $CDCl_3$) δ 8.87 (s, 1H), 7.55 – 7.40 (m, 2H), 7.27 – 7.09 (m, 2H), 6.13 (d, *J* = 48.5 Hz, 1H) ppm. $^{13}$C NMR (101 MHz, $CDCl_3$) δ 173.3 (dd, *J* = 28.1, 1.3 Hz), 160.4 (dd, *J* = 250.7, 4.4 Hz), 132.1 (dd, *J* = 8.3, 2.6 Hz), 128.9 (dd, *J* = 5.1, 2.6 Hz), 124.7 (dd, *J* = 4.1, 1.2 Hz), 121.2 (dd, *J* = 20.9, 13.9 Hz), 116.0 (d, *J* = 20.9 Hz), 83.2 (dd, *J* = 185.6, 3.9 Hz) ppm. $^{19}$F NMR (376 MHz, $CDCl_3$) δ -117.09 (m), -181.17 (d, *J* = 46.7 Hz) ppm. These data are in full agreement with those previously published in the literature. [7]

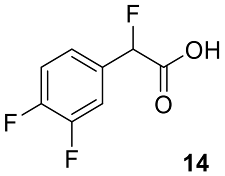


**14**

**2-(3,4-difluorophenyl)-2-fluoroacetic acid (14).** From 2-(3,4-difluorophenyl)acetic acid (309.8 mg, 1.8 mmol, 0.2 mM), using 4-(dimethylamino)pyridine (439.8 mg, 3.6 mmol, 2 equiv.) and 1-chloromethyl-4-fluoro-1,4-diazoniabicyclo[2.2.2]octanebis(tetrafluoroborate) (765.2 mg, 2.16 mmol, 1.2 equiv.) in 9 mL MeCN. The crude mixture was purified over silica with flash chromatography, using an eluent gradient of 0-10% EA in hexane to afford the title compound as white crystals (161.6 mg, 0.85 mmol, 47%).

$^{1}$H NMR (400 MHz, $CDCl_3$) δ 7.39– 7.34 (m, 1H), 7.28 – 7.20 (m, 2H), 5.82 (d, *J* = 47.0 Hz, 1H) ppm. $^{13}$C NMR (101 MHz, $CDCl_3$) δ 174.2 (d, *J* = 27.8 Hz), 151.4 (ddd, *J* = 249.8, 10.5, 2.1 Hz), 150.6 (dd, *J* = 249.7, 12.1 Hz), 130.2 (ddd, *J* = 21.6, 5.8, 3.9 Hz), 123.2 (td, *J* = 6.7, 3.8 Hz), 118.1 (d, *J* = 17.8 Hz), 116.0 (dd, *J* = 18.9, 6.6 Hz), 87.6 (dd, *J* = 188.8, 1.5 Hz) ppm. $^{19}$F NMR (376 MHz, $CDCl_3$) δ -135.10 – -135.16 (m), -135.54 – -135.63 (m), -181.48 (d, *J* = 47.2 Hz) ppm. HRMS (ESI) *m/z* calculated [M+2Na-H] 234.9911, found, 234.9918.

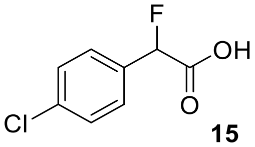


**15**

**2-(4-chlorophenyl)-2-fluoroacetic acid (15).** From 2-(4-chlorophenyl)acetic acid (307.8 mg, 1.8 mmol, 0.2 mM), using 4-(dimethylamino)pyridine (439.8 mg, 3.6 mmol, 2 equiv.) and 1-chloromethyl-4-fluoro-1,4-diazoniabicyclo[2.2.2]octanebis(tetrafluoroborate) (765.2 mg, 2.16 mmol, 1.2 equiv.) in 9 mL MeCN. The crude mixture was purified over silica with flash chromatography, using an eluent gradient of 0–10% EA in hexane to afford the title compound as a white solid (286.7 mg, 1.5 mmol, 84%).

$^{1}$H NMR (400 MHz, $CDCl_3$) δ 10.98 – 10.64 (m, 1H), 7.49 – 7.38 (m, 4H), 5.83 (d, *J* = 47.5 Hz, 1H) ppm. $^{13}$C NMR (101 MHz, $CDCl_3$) δ 174.1 (d, *J* = 32.3 Hz), 136.1 (d, *J* = 2.5 Hz), 131.8 (d, *J* = 21.1 Hz), 129.2, 127.9 (d, *J* = 6.2 Hz), 88.0 (d, *J* = 188.2 Hz) ppm. $^{19}$F NMR (376 MHz, $CDCl_3$) δ -181.97 (d, *J* = 47.2 Hz) ppm. These data are in full agreement with those previously published in the literature.[7]

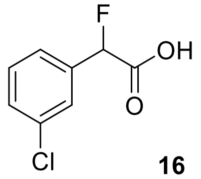

16

**2-(3-chlorophenyl)-2-fluoroacetic acid (16).** From 2-(3-chlorophenyl)acetic acid (307.8 mg, 1.8 mmol, 0.2 mM) using 4-(dimethylamino)pyridine (439.8 mg, 3.6 mmol, 2 equiv.) and 1-chloromethyl-4-fluoro-1,4-diazoniabicyclo[2.2.2]octanebis(tetrafluoroborate) (765.2 mg, 2.16 mmol, 1.2 equiv.) in 9 mL MeCN. The crude mixture was purified over silica with flash chromatography (30% EA in hexane) to afford the title compound as a yellow oil (164.5 mg, 0.87 mmol, 48%).

$^{1}$H NMR (400 MHz, $CDCl_3$) δ 7.47 (s, 1H), 7.39 – 7.34 (m, 3H), 5.77 (d, $J$ = 47.3 Hz, 1H) ppm. $^{13}$C NMR (101 MHz, $CDCl_3$) δ 168.6 (d, $J$ = 27.3 Hz), 136.1 (d, $J$ = 21.0 Hz), 134.9, 130.23, 129.9 (d, $J$ = 1.8 Hz), 126.8 (d, $J$ = 6.8 Hz), 124.8 (d, $J$ = 6.3 Hz), 88.6 (d, $J$ = 187.2 Hz) ppm. $^{19}$F NMR (376 MHz, $CDCl_3$) δ -182.24 (d, $J$ = 47.4 Hz) ppm. These data are in full agreement with those previously published in the literature.[7]

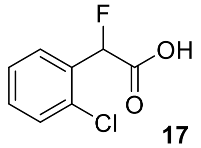

17

**2-(2-chlorophenyl)-2-fluoroacetic acid (17).** From 2-(2-chlorophenyl)acetic acid (307.8 mg, 1.8 mmol, 0.2 mM) using 4-(dimethylamino)pyridine (439.8 mg, 3.6 mmol, 2 equiv.) and 1-chloromethyl-4-fluoro-1,4-diazoniabicyclo[2.2.2]octanebis(tetrafluoroborate) (765.2 mg, 2.16 mmol, 1.2 equiv.) in 9 mL MeCN. The crude mixture was purified over silica with flash chromatography (30% EA in hexane) to afford the title compound as a yellowish powder (274.5 mg, 1.5 mmol, 81%).

$^{1}$H NMR (400 MHz, MeOD-$d_4$): δ 7.57–7.35 (m, 4H), 6.24 (d, $J$ = 46.8 Hz, 1H) ppm. $^{13}$C NMR (100 MHz, MeOD-$d_4$): δ 170.1 (d, $J$ = 27.4 Hz), 133.5 (d, $J$ = 4.1 Hz), 132.9 (d, $J$ = 20.2 Hz), 130.9 (d, $J$ = 2.6 Hz), 129.7 (d, $J$ = 1.1 Hz), 128.8 (d, $J$ = 5.5 Hz), 127.2 (d, $J$ = 1.2 Hz), 86.1 (d, $J$ = 182.1 Hz) ppm. $^{19}$F NMR (376 MHz, MeOD-$d_4$): δ -180.44 (d, $J$ = 45.7 Hz) ppm. HRMS (ESI) *m/z* calculated [M+2Na-H] 232.9711, found, 232.9720.

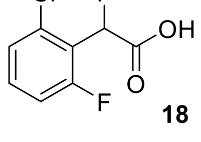

18

**2-(2-chloro-6-fluorophenyl)-2-fluoroacetic acid (18).** From2-(2-chloro-6-fluorophenyl)acetic acid (339.44 mg, 1.8 mmol, 0.2 mM), using 4-(dimethylamino)pyridine (439.8 mg, 3.6 mmol, 2 equiv.) and 1-chloromethyl-4-fluoro-1,4-diazoniabicyclo[2.2.2]octanebis(tetrafluoroborate) (765.2 mg, 2.16 mmol, 1.2 equiv.) in 9 mL MeCN. A yield of 64% was determined by $^{1}$H NMR, using dimethyl maleate as an internal standard. The product could not be separated from the substrate by column chromatography.

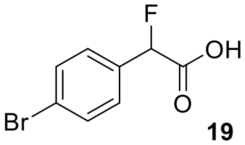


**19**

**2-(4-bromophenyl)-2-fluoroacetic acid (19).** From 2-(4-bromophenyl)acetic acid (387.9 mg, 1.8 mmol, 0.2 mM), using 4-(dimethylamino)pyridine (439.8 mg, 3.6 mmol, 2 equiv.) and 1-chloromethyl-4-fluoro-1,4-diazoniabicyclo[2.2.2]octanebis(tetrafluoroborate) (765.2 mg, 2.16 mmol, 1.2 equiv.) in 9 mL MeCN. The crude mixture was purified over silica with flash chromatography (40% EA in hexane) to afford the title compound as white powder (254.5 mg, 1.2 mmol, 61%).

$^1$H NMR (400 MHz, $CDCl_3$): δ 7.58 (d, $J$ = 7.8 Hz, 2H), 7.38 (d, $J$ = 8.2 Hz, 2H) 5.81 (d, $J$ = 47.0 Hz, 1H) ppm. $^{13}$C NMR (101 MHz, $CDCl_3$) δ 172.8 (d, $J$ = 27.8 Hz), 133.1 (d, $J$ = 20.8 Hz), 132.7 (d, $J$ = 21.0 Hz), 132.2, 128.3 (d, $J$ = 5.8 Hz), 124.3 (d, $J$ = 2.5 Hz), 88.8 (d, $J$ = 186.4 Hz), 88.3 (d, $J$ = 187.2 Hz) ppm. $^{19}$F NMR (376 MHz, $CDCl_3$) δ -182.43 (d, $J$ = 49 Hz) ppm. These data are in full agreement with those previously published in the literature.[8]

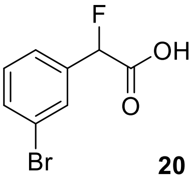


**20**

**2-(3-bromophenyl)-2-fluoroacetic acid (20).** From 2-(3-bromophenyl)acetic acid (387.9 mg, 1.8 mmol, 0.2 mM), using 4-(dimethylamino)pyridine (439.8 mg, 3.6 mmol, 2 equiv.) and 1-chloromethyl-4-fluoro-1,4-diazoniabicyclo[2.2.2]octanebis(tetrafluoroborate) (765.2 mg, 2.16 mmol, 1.2 equiv.) in 9 mL MeCN. The crude mixture was purified over silica with flash chromatography (40% EA in hexane) to afford the title compound as white powder (157.6 mg, 0.68 mmol, 38%).

$^1$H NMR (400 MHz, $CDCl_3$) δ 10.94 (s, 1H), 7.61 (s, 1H), 7.53– 7.51 (m, 1H), 7.40– 7.37 (m, 1H), 7.28– 7.24 (m, 1H) 5.76 (d, $J$ = 47.1 Hz, 1H) ppm. $^{13}$C NMR (101 MHz, $CDCl_3$) δ 174.0 (d, $J$ = 27.7 Hz), 135.5 (d, $J$ = 20.8 Hz), 133.2 (d, $J$ = 1.8 Hz), 130.6, 129.6 (d, $J$ = 6.8 Hz), 125.2 (d, $J$ = 6.3 Hz), 123.0, 87.9 (d, $J$ = 188.8 Hz) ppm. $^{19}$F NMR (376 MHz, $CDCl_3$) δ -183.16 (d, $J$ = 46.9 Hz) ppm. These data are in full agreement with those previously published in the literature.[8]

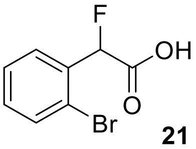


**21**

**2-(2-bromophenyl)-2-fluoroacetic acid (21).** From 2-(2-bromophenyl)acetic acid (387.9 mg, 1.8 mmol, 0.2 mM), using 4-(dimethylamino)pyridine (439.8 mg, 3.6 mmol, 2 equiv.) and 1-chloromethyl-4-fluoro-1,4-diazoniabicyclo[2.2.2]octanebis(tetrafluoroborate) (765.2 mg, 2.16 mmol, 1.2 equiv.) in 9 mL MeCN. The crude mixture was purified over silica with flash chromatography (40% EA in hexane) to afford the title compound as white powder (212.6 mg, 0.91 mmol, 51%).

$^1$H NMR (400 MHz, $CDCl_3$) δ 7.63 (d, $J$ = 8.1 Hz, 1H), 7.54 –7.51 (m, 1H), 7.42 – 7.37 (m, 1H), 7.32 – 7.28 (m, 1H), 6.28 (d, $J$ = 46.3 Hz, 1H) ppm. $^{13}$C NMR (101 MHz, $CDCl_3$) δ 172.9 (d, $J$ = 28.1 Hz), 133.4, 133.3 (d, $J$ = 20.7 Hz), 131.5 (d, $J$ = 2.5 Hz), 128.7 (d, $J$ = 6.2 Hz), 128.0, 123.4 (d, $J$ = 4.7 Hz), 87.9 (d, $J$ = 186.9 Hz) ppm. $^{19}$F NMR (376 MHz, $CDCl_3$) δ -180.05 (d, $J$ = 46.2 Hz) ppm. HRMS (ESI) *m/z* calculated [M+2Na-H] 276.9211, found, 278.9203.

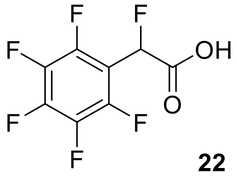

**22**

**2-Fluoro-2-(perfluorophenyl)acetic acid (22)**. From 2-(perfluorophenyl)acetic acid (406.9 mg, 1.8 mmol, 0.2 mM), using 4-(dimethylamino)pyridine (439.8 mg, 3.6 mmol, 2 equiv.) and 1-chloromethyl-4-fluoro-1,4-diazoniabicyclo[2.2.2]octanebis(tetrafluoroborate) (765.2 mg, 2.16 mmol, 1.2 equiv.) in 9 mL MeCN. The crude mixture was purified over silica with flash chromatography, using an eluent gradient of 0-10% EA in hexane to afford the title compound as yellow oil (163.8 mg, 0.67 mmol 38%).

$^{1}$H NMR (400 MHz, DMSO) δ 14.17 (s, 1H), 6.48 (d, *J* = 44.7 Hz, 1H) ppm. $^{13}$C NMR (101 MHz, DMSO) δ 167.5 (d, *J* = 26.0 Hz), 146.1, 143.6, 140.7, 138.5, 136.02 109.4 (d, *J* = 17.0 Hz), 78.6 (d, *J* = 185.2 Hz) ppm. $^{19}$F NMR (376 MHz, DMSO) δ -142.28 – -142.42 (m), -149.81 – -151.43 (m), -160.86 – -161.85 (m), -180.97 (d, *J* = 44.5 Hz) ppm. HRMS (ESI) *m/z* calculated [M+2Na-H], 288.9711 found, 288.9639.

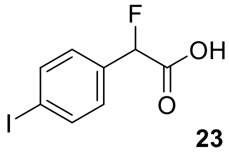

**23**

**2-fluoro-2-(4-iodophenyl)acetic acid (23).** From 2-(4-iodophenyl)acetic acid ( 225.8 mg, 1.8 mmol, 0.2 mM), using 4-(dimethylamino)pyridine (439.8 mg, 3.6 mmol, 2 equiv.) and 1-chloromethyl-4-fluoro-1,4-diazoniabicyclo[2.2.2]octanebis(tetrafluoroborate) (765.2 mg, 2.16 mmol, 1.2 equiv.) in 9 mL MeCN. The crude mixture was purified over silica with flash chromatography, using an eluent gradient of 0–10% EA in hexane to afford the title compound as a white solid (86.2 mg, 0.31 mmol, 35%).

$^{1}$H NMR (400 MHz, $CDCl_3$) δ 7.80 (d, *J* = 7.6 Hz, 2H), 7.26 (d, *J* = 8.7 Hz, 2H), 5.81 (d, *J* = 47.2 Hz, 1H) ppm. $^{13}$C NMR (101 MHz, $CDCl_3$) δ 173.9 (d, *J* = 27.6 Hz), 138.2, 133.1 (d, *J* = 20.9 Hz), 128.4 (d, *J* = 6.2 Hz), 96.3 (d, *J* = 2.7 Hz), 88.3 (d, *J* = 188.1 Hz) ppm. $^{19}$F NMR (376 MHz, $CDCl_3$) δ -182.15 (d, *J* = 47.0 Hz) ppm. HRMS (ESI) *m/z* calculated [M+2Na-H] 324.9111, found, 324.9089.

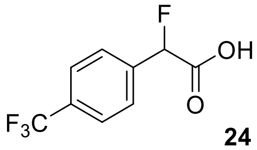

**24**

**2-Fluoro-2-(4-(trifluoromethyl)phenyl)acetic acid (24).** From 2-(4-(trifluoromethyl)phenyl)acetic acid (367.47 1.8 mmol, 0.2 mM) using 4-(dimethylamino) pyridine (439.8 mg, 3.6 mmol, 2 equiv.) and 1-chloromethyl-4-fluoro-1,4-diazoniabicyclo[2.2.2]octanebis(tetrafluoroborate) (765.2 mg, 2.16 mmol, 1.2 equiv.) in 9 mL MeCN. The crude mixture was purified over silica with flash chromatography (10% EA in hexane) to afford the title compound as yellow crystals (343.3 mg, 1.55 mmol, 86%).

$^{1}$H NMR (400 MHz, $CDCl_3$) δ 11.54 (s, 1H), 7.83 – 7.50 (m, 4H), 5.90 (d, *J* = 47.1 Hz, 1H) ppm. $^{13}$C NMR (101 MHz, $CDCl_3$) δ 173.9 (d, *J* = 27.2 Hz), 137.2 (d, *J* = 21.1 Hz), 132.7–131.7 (m), 126.9 (d, *J* = 6.7 Hz), 126.0 (d, *J* = 3.8 Hz), 88.1 (d, *J* = 189.0 Hz) ppm. $^{19}$F NMR (376 MHz, $CDCl_3$) δ -62.95, -185.25 (d, *J* = 47.3 Hz) ppm. These data are in full agreement with those previously published in the literature.[8]

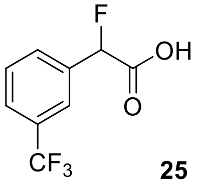

**25**

**2-fluoro-2-(3-(trifluoromethyl)phenyl)acetic acid (25).** From 2-(3-(trifluoromethyl)phenyl)acetic acid (367.47 mg, 1.8 mmol, 0.2 mM) using 4-(dimethylamino)-pyridine (439.8 mg, 3.6 mmol, 2 equiv.) and 1-chloromethyl-4-fluoro-1,4-diazoniabicyclo[2.2.2]octanebis(tetrafluoroborate) (765.2 mg, 2.16 mmol, 1.2 equiv.) in 9 mL MeCN. The crude mixture was purified over silica with flash chromatography using an eluent gradient of 0-20% EA in hexane to afford the title compound as a yellowish powder (126.6 mg, 0.57 mmol, 32%).

$^{1}$H NMR (400 MHz, $CDCl_3$) δ 9.67 (s, 1H), 7.79 (s, 1H), 7.72 (d, *J* = 7.9 Hz, 2H), 7.59 (t, *J* = 7.8 Hz, 1H), 5.93 (d, *J* = 47.2, 1H) ppm. $^{13}$C NMR (101 MHz, $CDCl_3$) δ 173.2 (d, *J* = 27.2 Hz), 134.3 (d, *J* = 21.4 Hz), 131.3 (q, *J* = 32.9 Hz), 129.7 (d, *J* = 6.5 Hz), 129.5, 126.8– 126.7 (m), 123.2– 123.3 (m), 87.9 (d, *J* = 188.9 Hz) ppm.$^{19}$F NMR (376 MHz, $CDCl_3$) δ -62.81, -184.25 (d, *J* = 46.7 Hz) ppm. HRMS (ESI) *m/z* calculated [M+2Na-H] 267.0011, found, 266.9985.

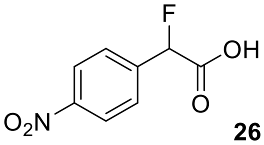

**26**

**2-fluoro-2-(4-nitrophenyl)acetic acid (26).** From 2-fluoro-2-(4-nitrophenyl)acetic acid (326.07 mg, 1.8 mmol, 0.2 mM) using 4-(dimethylamino)pyridine (439.8 mg, 3.6 mmol, 2 equiv.) and 1-chloromethyl-4-fluoro-1,4-diazoniabicyclo[2.2.2]octanebis(tetrafluoroborate) (765.2 mg, 2.16 mmol, 1.2 equiv.) in 9 mL MeCN. A yield of 87% was determined by $^{1}$H NMR, using dimethyl maleate as an internal standard. Isolation was not possible, because the title compound decomposed during column chromatography.

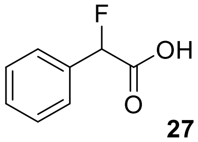

**27**

**2-fluoro-2-phenylacetic acid (27).** From 2-phenylacetic acid (245.07 mg, 1.8 mmol, 0.2 mM), using 4-(dimethylamino)pyridine (439.8 mg, 3.6 mmol, 2 equiv.) and 1-chloromethyl-4-fluoro-1,4-diazoniabicyclo[2.2.2]octanebis(tetrafluoroborate) (765.2 mg, 2.16 mmol, 1.2 equiv.) in 9 mL MeCN. The crude mixture was purified over silica with flash chromatography (5% EA in hexane) to afford the title compound as white crystals (136.8 mg, 0.89 mmol, 49%).

$^{1}$H NMR (400 MHz, $CDCl_3$): δ 10.40 (s, 1H) 7.61–7.33 (m, 5H), 5.86 (d, *J* = 47.4 Hz, 1H) ppm. $^{13}$C NMR (101 MHz, $CDCl_3$) δ 174.6 (d, *J* = 28.0 Hz), 133.5 (d, *J* = 20.4 Hz), 130.1 (d, *J* = 2.2 Hz), 129.0, 126.8 (d, *J* = 6.1 Hz), 88.9 (d, *J* = 186.6Hz) ppm. $^{19}$F NMR (376 MHz, $CDCl_3$): δ -180.82 (d, *J* = 47.5 Hz) ppm. These data are in full agreement with those previously published in the literature.[8]

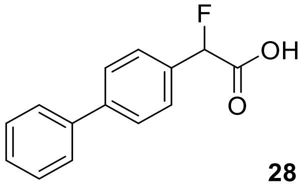

**28**

**2-([1,1'-biphenyl]-4-yl)-2-fluoroacetic acid) (28)**. From 2-([1,1'-biphenyl]-4-yl)acetic acid (382.05 mg, 1.8 mmol, 0.2 mM) using 4-(dimethylamino)-pyridine (439.8 mg, 3.6 mmol, 2 equiv.) and 1-chloromethyl-4-fluoro-1,4-diazoniabicyclo[2.2.2]octanebis(tetrafluoroborate) (765.2 mg, 2.16 mmol, 1.2 equiv.) in 9 mL MeCN. The crude mixture was purified over silica with flash chromatography using an eluent gradient of 5–10 % EA in hexane to afford the title compound as a yellowish powder (308.7 mg, 1.34 mmol, 75%).

$^{1}$H NMR (400 MHz, MeOD) δ 7.68 (d, *J* = 7.7 Hz, 2H), 7.63 (d, *J* = 8.3 Hz, 2H), 7.55 (d, *J* = 8.0 Hz, 2H), 7.44 (t, *J* = 7.3 Hz, 2H), 7.35 (t, *J* = 6.6 Hz, 1H), 5.90 (d, *J* = 47.9 Hz, 1H). $^{13}$C NMR (100 MHz, MeOD-$d_4$): δ 170.8 (d, *J* = 28.0 Hz), 141.2 (d, *J* = 214.3 Hz), 134.0 (d, *J* = 20.4 Hz), 128.6 – 126.6 (m), 88.9 (d, *J* = 182.1 Hz) ppm. $^{19}$F NMR (376 MHz, MeOD-$d_4$): δ -179.43 (d, *J* = 47.5 Hz) ppm. These data are in full agreement with those previously published in the literature.[7]

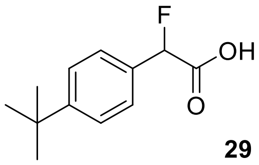

**29**

**2-(4-(*tert*-butyl)phenyl)-2-fluoroacetic acid (29).** From 2-(4-(tert-butyl)phenyl)acetic acid (346.06mg, 1.8 mmol, 0.2 mM), using 4-(dimethylamino)pyridine (439.8 mg, 3.6 mmol, 2 equiv.) and 1-chloromethyl-4-fluoro-1,4-diazoniabicyclo[2.2.2]octanebis(tetrafluoroborate) (765.2 mg, 2.16 mmol, 1.2 equiv.) in 9 mL MeCN. The crude mixture was purified over silica with flash chromatography, using an eluent gradient of 0–10% EA in hexane to afford the title compound as white crystals (185.4 mg, 0.88 mmol, 49%).

$^{1}$H NMR (400 MHz, $CDCl_3$) δ 10.50 (s, 1H), 7.48 – 7.39 (m, 4H), 5.80 (d, *J* = 47.5 Hz, 1H) 1.33 (s, 9H) ppm. $^{13}$C NMR (101 MHz, $CDCl_3$) δ 174.7 (d, *J* = 28.3 Hz), 153.4 (d, *J* = 2.3 Hz), 130.5 (d, *J* = 20.6 Hz), 126.7 (d, *J* = 5.7 Hz), 126.0, 88.8 (d, *J* = 186.1 Hz), 34.9, 31.3 ppm. $^{19}$F NMR (376 MHz, $CDCl_3$) δ -179.39 (d, *J* = 47.5 Hz) ppm. These data are in full agreement with those previously published in the literature.[8]

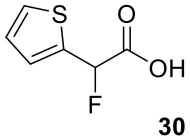

**30**

**2-fluoro-2-(thiophen-2-yl)acetic acid (30).** From 2-(thiophen-2-yl)acetic acid (255.02 mg, 1.8 mmol, 0.2 mM), using 4-(dimethylamino)pyridine (439.8 mg, 3.6 mmol, 2 equiv.) and 1-chloromethyl-4-fluoro-1,4-diazoniabicyclo[2.2.2]octanebis(tetrafluoroborate) (765.2 mg, 2.16 mmol, 1.2 equiv.) in 9 mL MeCN. The yield of 83% was determined by $^{1}$H NMR, using dimethyl maleate as an internal standard. Isolation was not possible, because the title compound decomposed during column chromatography.

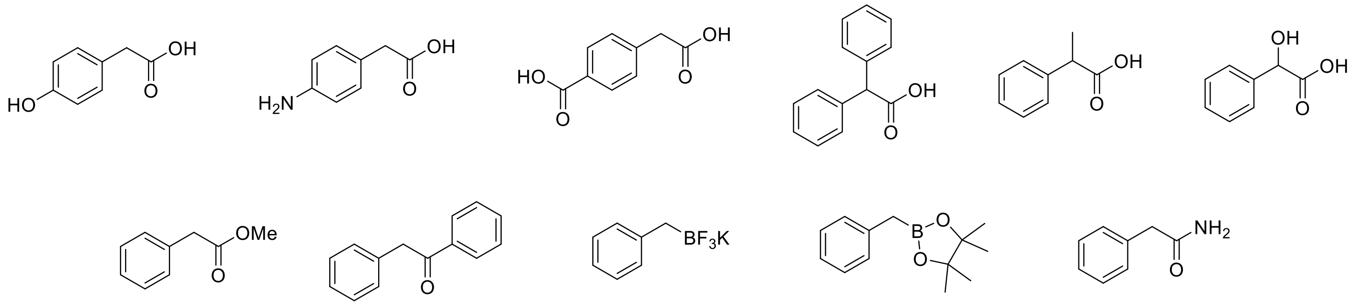

**Scheme S6.** Unsuccessful substrates

## 6 References


(1) Blessley, G.; Holden, P.; Walker, M.; Brown, J. M.; Gouverneur, V. Palladium-Catalyzed Substitution and Cross-Coupling of Benzylic Fluorides. *Org. Lett.* **2012**, *14*, 2754-2757.

(2) Tanaka, T.; Yazaki, R.; Ohshima, T. Chemoselective Catalytic α-Oxidation of Carboxylic Acids: Iron/Alkali Metal Cooperative Redox Active Catalysis. *J. Am. Chem. Soc.* **2020**, *142*, 4517-4524.

(3) Ye, L.; Tian, Y.; Meng, X.; Gu, Q.-S.; Liu, X.-Y. Enantioselective Copper(I)/Chiral Phosphoric Acid Catalyzed Intramolecular Amination of Allylic and Benzylic C−H Bonds. *Angew. Chem. Int. Ed.* **2020**, *59*, 1129-1133.

(4) Hua, A. M.; Bidwell, S. L.; Baker, S. I.; Hratchian, H. P.; Baxter, R. D. Experimental and Theoretical Evidence for Nitrogen–Fluorine Halogen Bonding in Silver-Initiated Radical Fluorinations. *ACS Catal.* **2019**, *9*, 3322-3326.

(5) Sundaraganesan, N.; Kalaichelvan, S.; Meganathan, C.; Joshua, B. D.; Cornard, J. FT-IR, FT-Raman spectra and ab initio HF and DFT calculations of 4-N,N′ -dimethylamino pyridine. *Spectrochim. Acta A Mol. Biomol. Spectros.* **2008**, *71*, 898-906.

(6) Olah, G. A.; Welch, J. T.; Vankar, Y. D.; Nojima, M.; Kerekes, I.; Olah, J. A. Synthetic methods and reactions. 63. Pyridinium poly(hydrogen fluoride) (30% pyridine-70% hydrogen fluoride): a convenient reagent for organic fluorination reactions. *J. Org. Chem.* **1979**, *44*, 3872-3881.

(7) Wang, H.; Liu, C.-F.; Song, Z.; Yuan, M.; Ho, Y. A.; Gutierrez, O.; Koh, M. J. Engaging α-Fluorocarboxylic Acids Directly in Decarboxylative C–C Bond Formation. *ACS Catal.* **2020**, *10*, 4451-4459.

(8) Sap, J. B. I.; Wilson, T. C.; Kee, C. W.; Straathof, N. J. W.; Ende, C. W. a.; Mukherjee, P.; Zhang, L.; Genicot, C.; Gouverneur, V. Synthesis of 18F-difluoromethylarenes using aryl boronic acids, ethyl bromofluoroacetate and [18F]fluoride. *Chem. Sci.* **2019**, *10*, 3237-3241.

# Copies of NMR spectra of isolated compounds

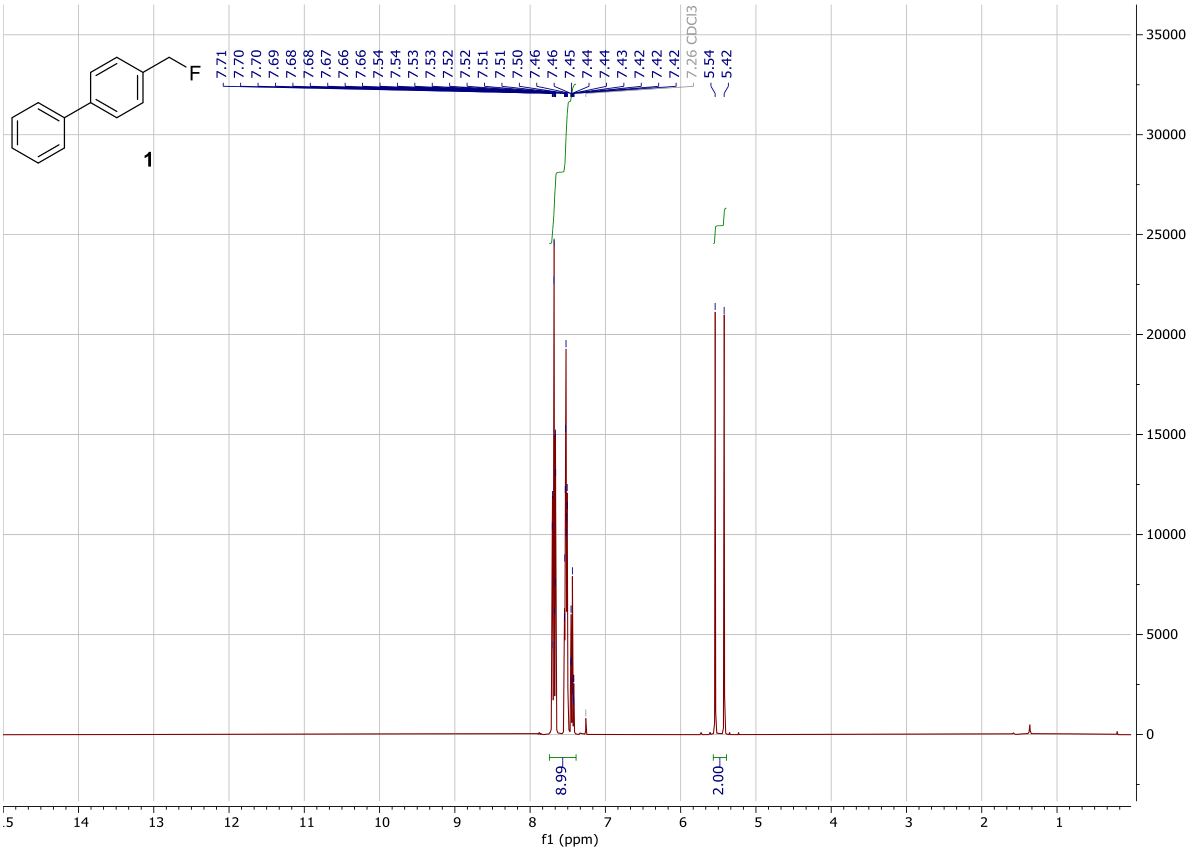

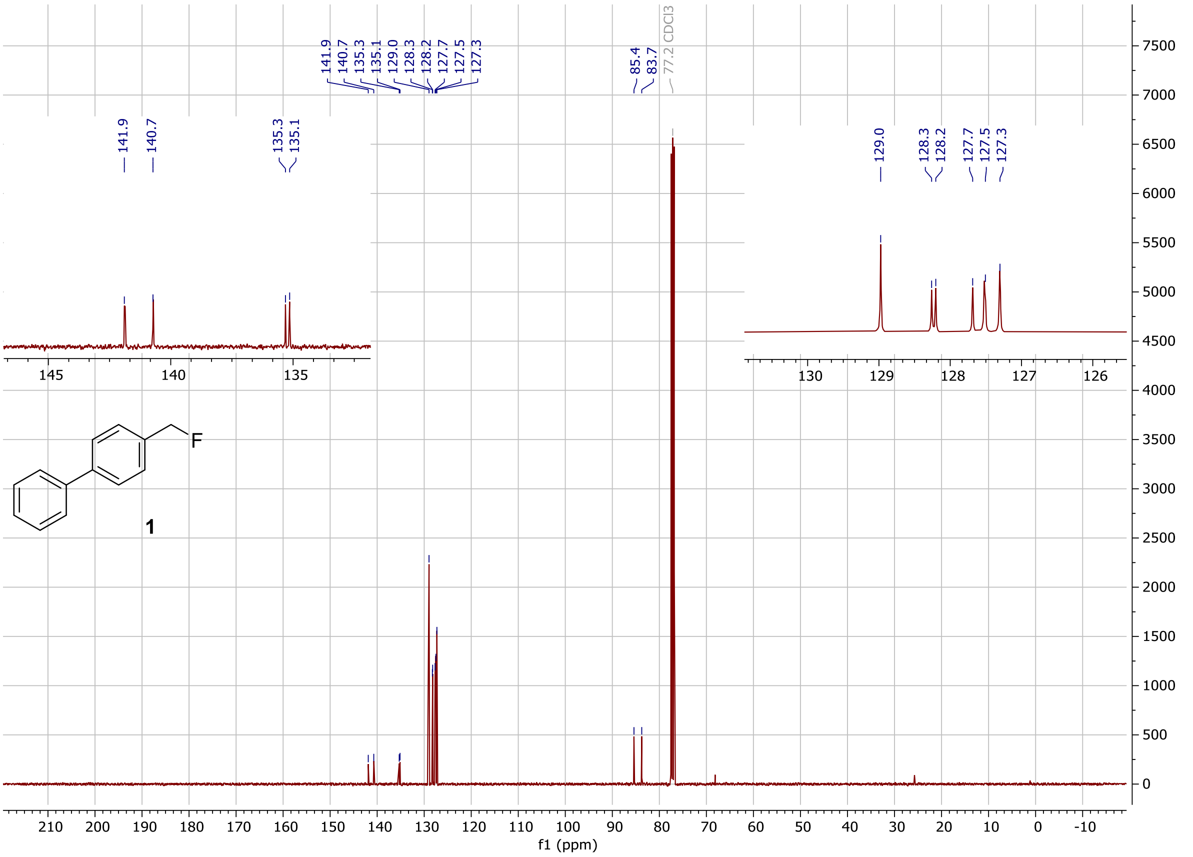

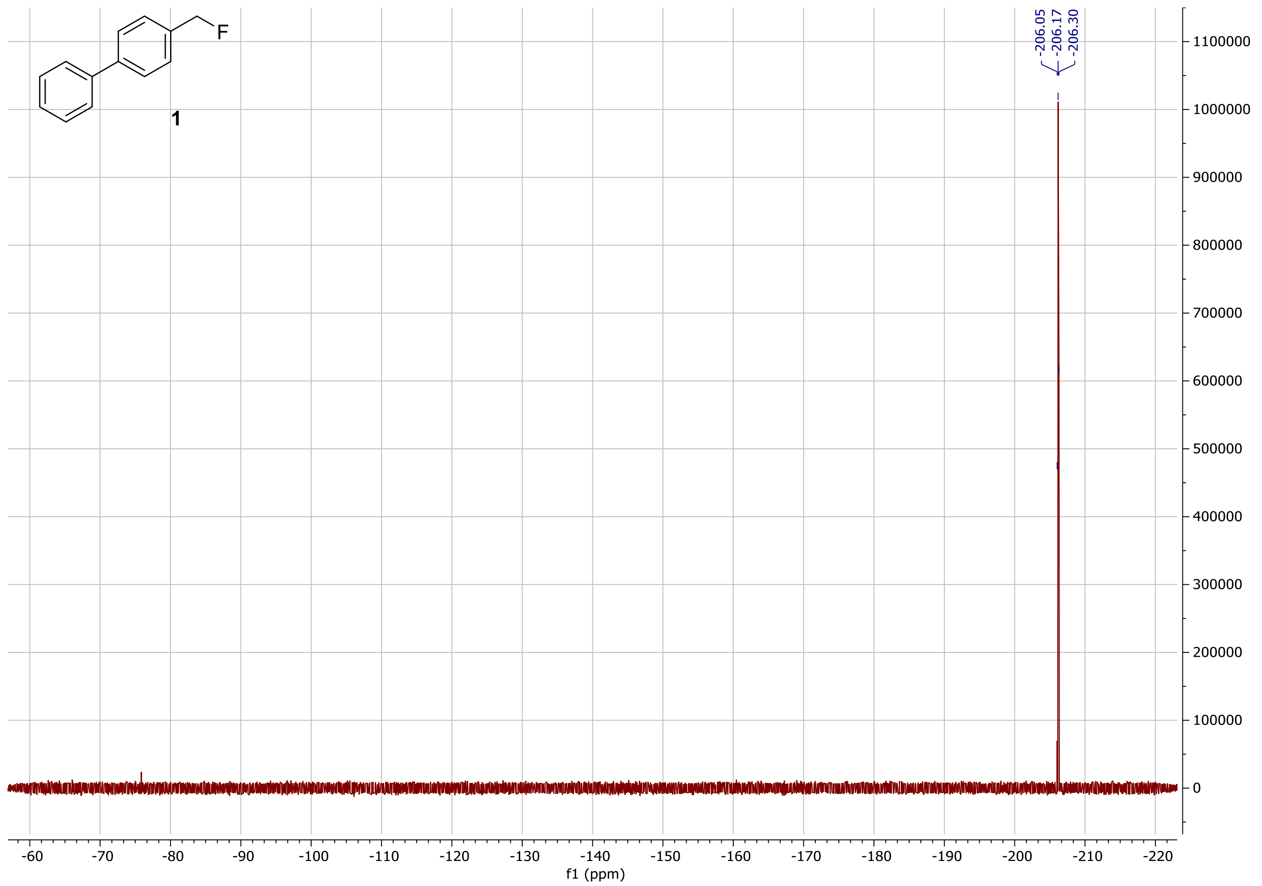

F
1
-206.05
-206.17
-206.30
f1 (ppm)


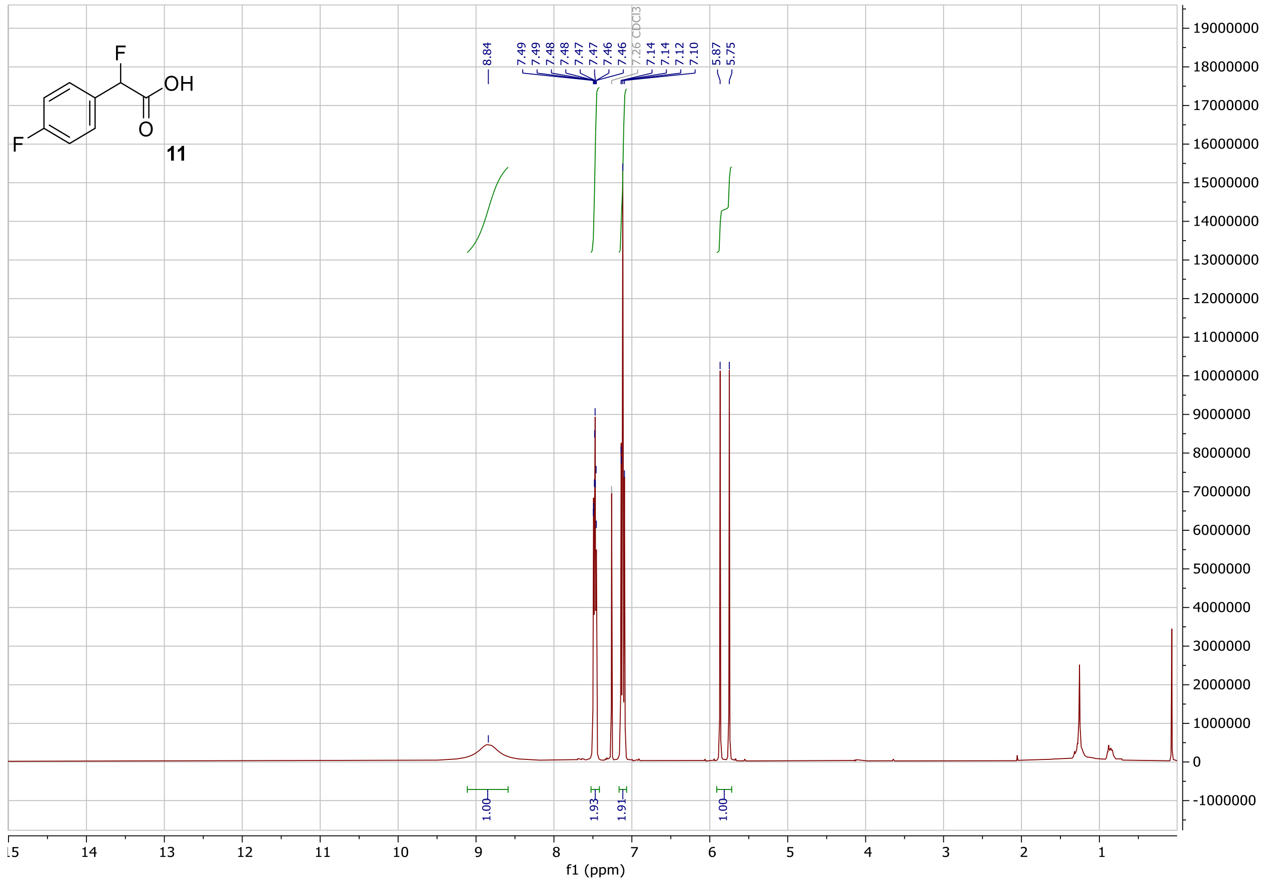

F
OH
O
F
11
8.84
7.49
7.49
7.48
7.48
7.47
7.46
7.46
7.26 CDCl3
7.14
7.14
7.12
7.10
5.87
5.75
1.00
1.93
1.91
1.00
f1 (ppm)

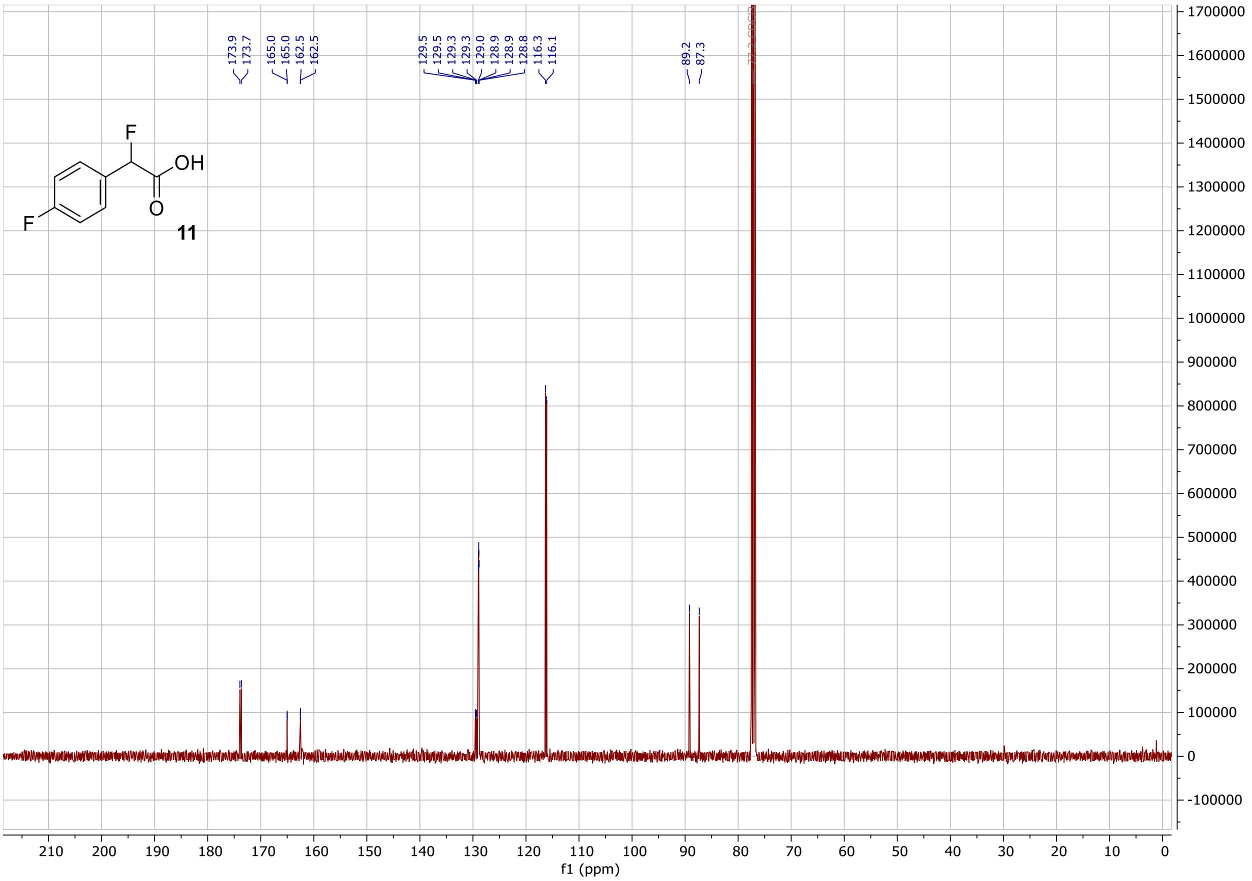
173.9
173.7
165.0
165.0
162.5
162.5
129.5
129.5
129.3
129.3
129.0
128.9
128.9
128.8
116.3
116.1
89.2
87.3
F
OH
O
F
11
f1 (ppm)

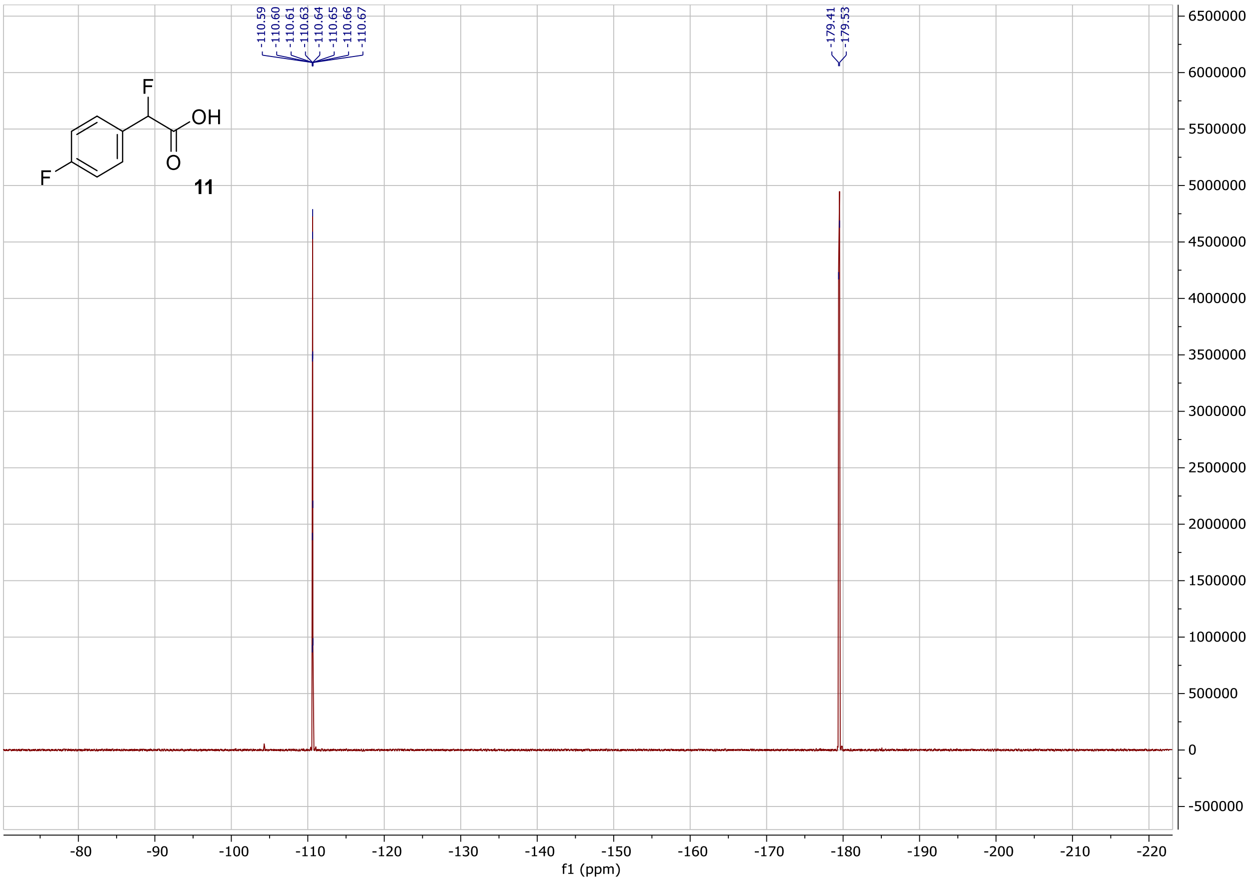
-110.59
-110.60
-110.61
-110.63
-110.64
-110.65
-110.66
-110.67
-179.41
-179.53
F
OH
O
F
11
f1 (ppm)

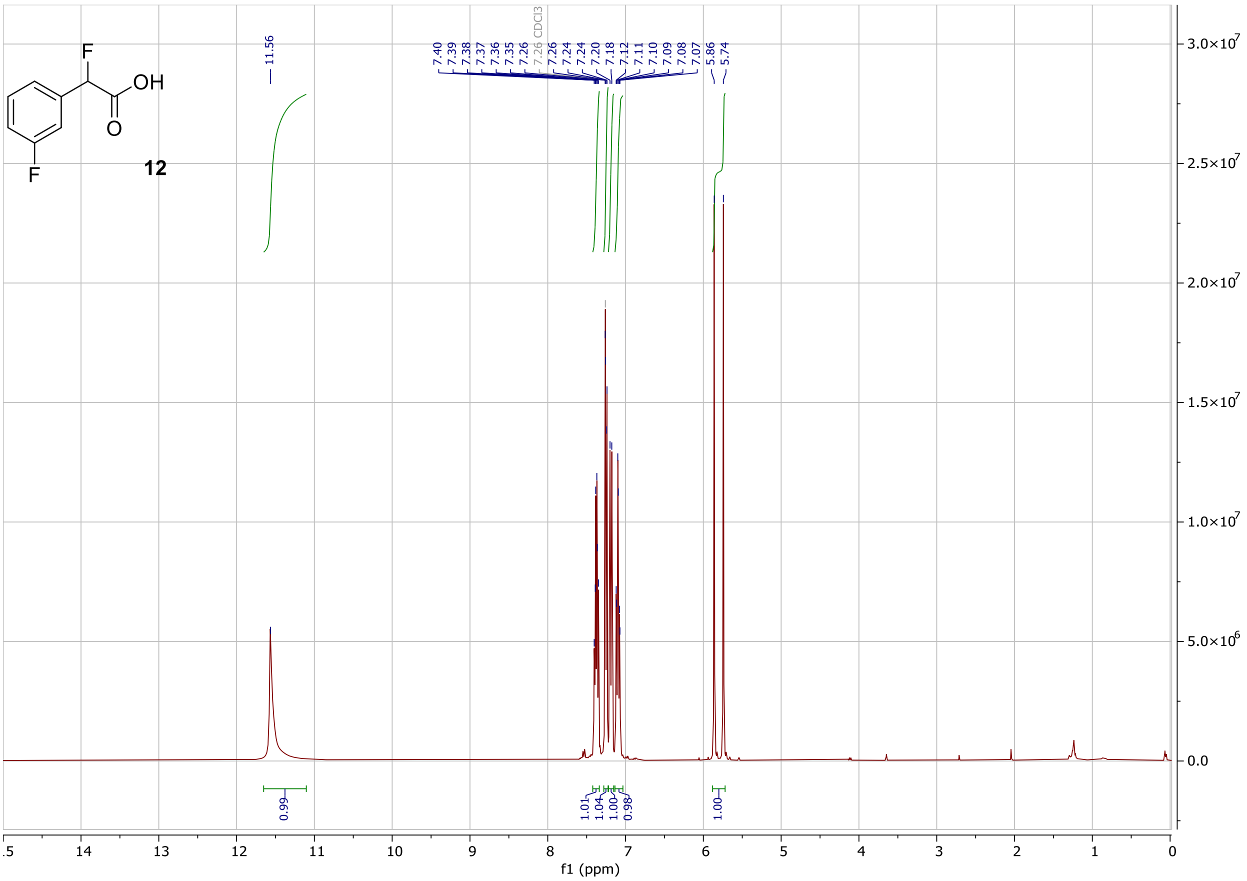
F
OH
O
F
12
11.56
7.40
7.39
7.38
7.37
7.36
7.35
7.26
7.26 CDCl3
7.26
7.24
7.24
7.20
7.18
7.12
7.11
7.10
7.09
7.08
7.07
5.86
5.74
0.99
1.01
1.04
1.00
0.98
1.00
f1 (ppm)
14
13
12
11
10
9
8
7
6
5
4
3
2
1
0
3.0×10^7
2.5×10^7
2.0×10^7
1.5×10^7
1.0×10^7
5.0×10^6
0.0

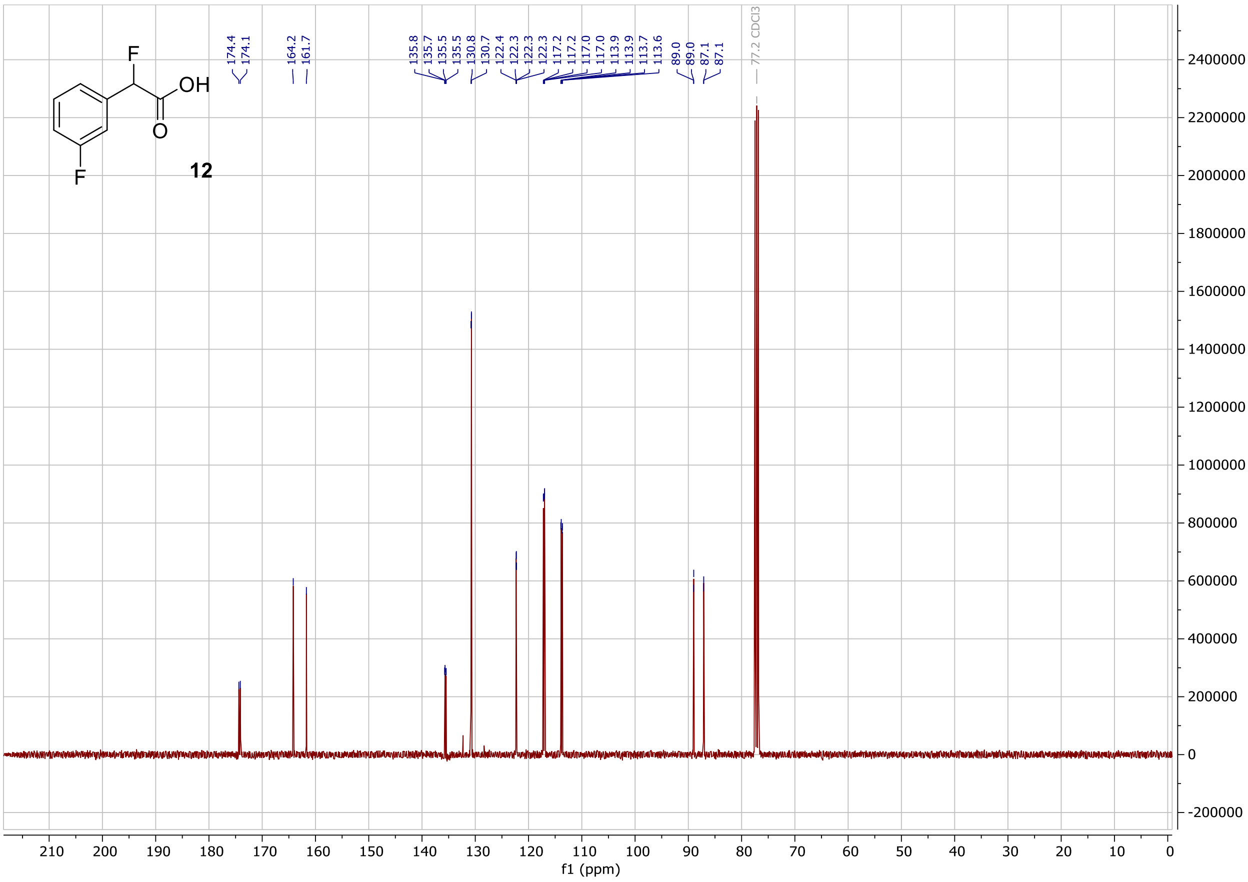
F
OH
O
F
12
174.4
174.1
164.2
161.7
135.8
135.7
135.5
135.5
130.8
130.7
122.4
122.3
122.3
122.3
117.2
117.2
117.0
117.0
113.9
113.7
113.6
89.0
89.0
87.1
87.1
77.2 CDCl3
f1 (ppm)
210
200
190
180
170
160
150
140
130
120
110
100
90
80
70
60
50
40
30
20
10
0
2400000
2200000
2000000
1800000
1600000
1400000
1200000
1000000
800000
600000
400000
200000
0
-200000

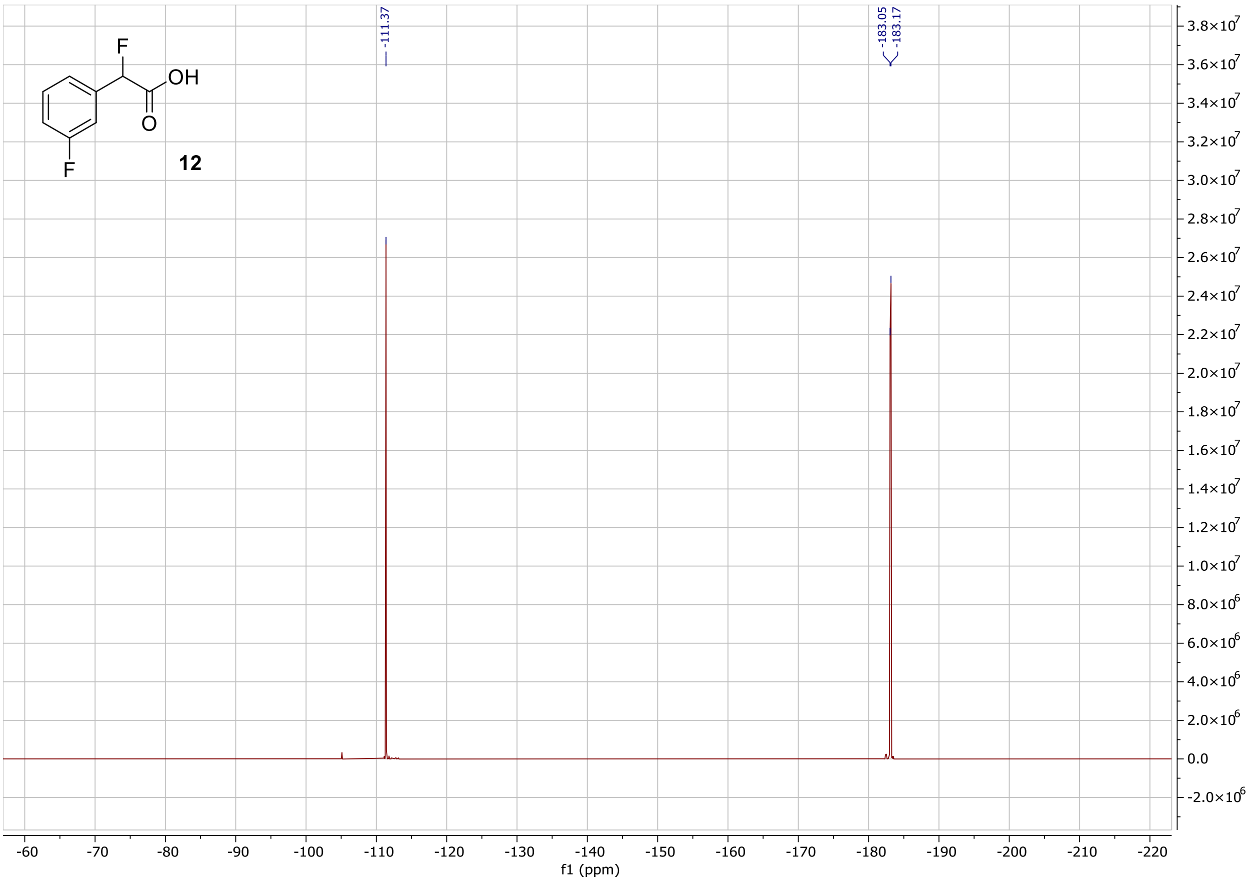

F
OH
O
F
12
-111.37
-183.05
-183.17
f1 (ppm)


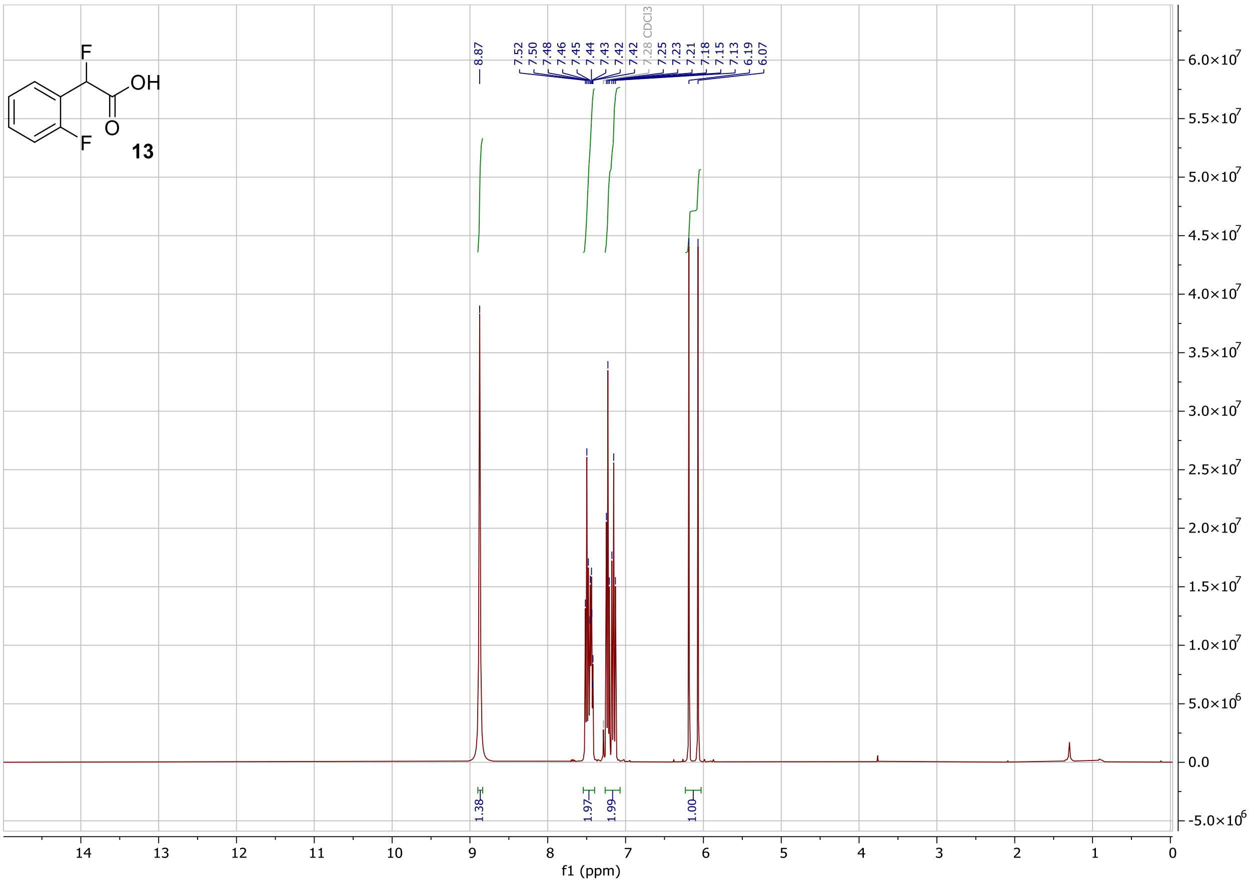

F
OH
O
F
13
8.87
7.52
7.50
7.48
7.46
7.45
7.44
7.43
7.42
7.42
7.28 CDCl3
7.25
7.23
7.21
7.18
7.15
7.13
6.19
6.07
1.38
1.97
1.99
1.00
f1 (ppm)

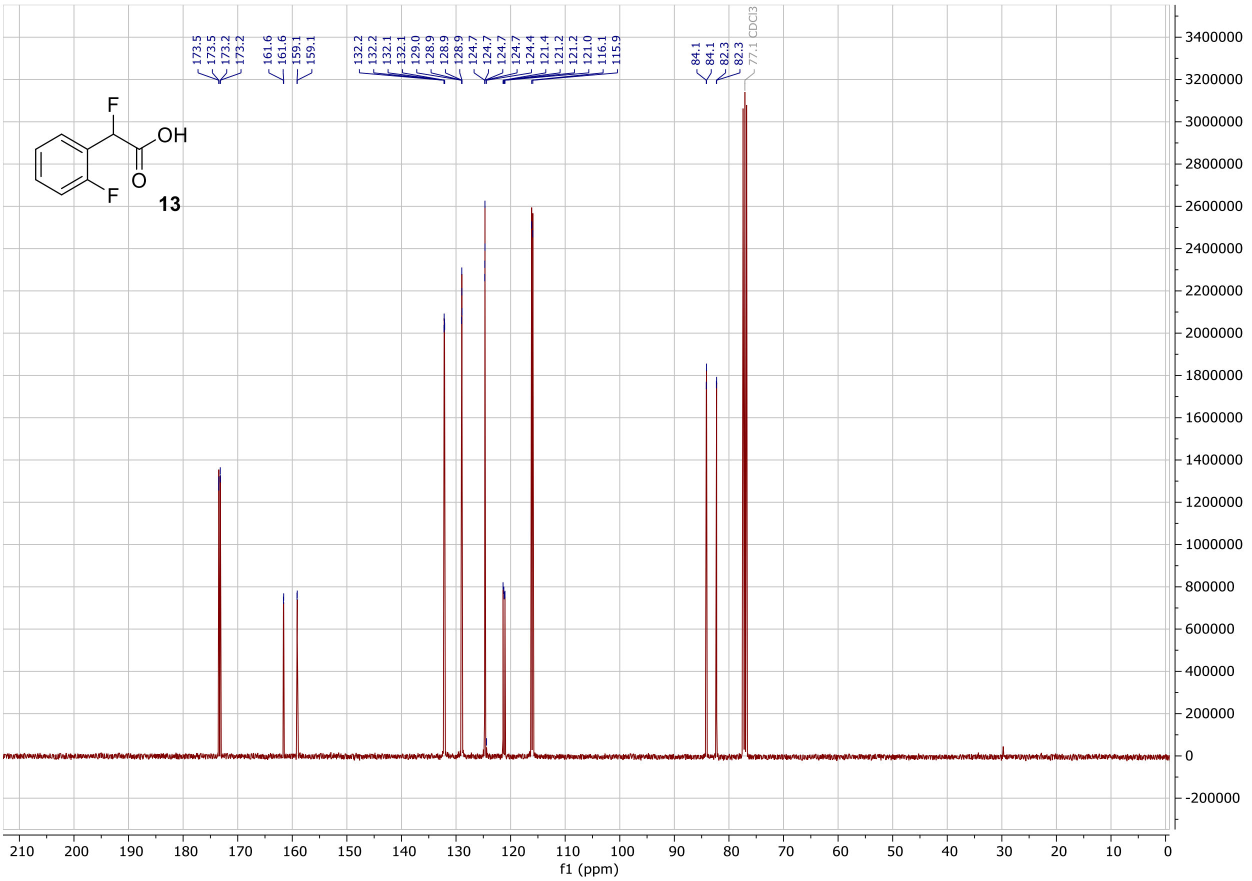

13
173.5
173.5
173.2
173.2
161.6
161.6
159.1
159.1
132.2
132.2
132.1
132.1
129.0
128.9
128.9
128.9
124.7
124.7
124.7
124.7
124.4
121.4
121.2
121.2
121.0
116.1
115.9
84.1
84.1
82.3
82.3
77.1 CDCl3
f1 (ppm)


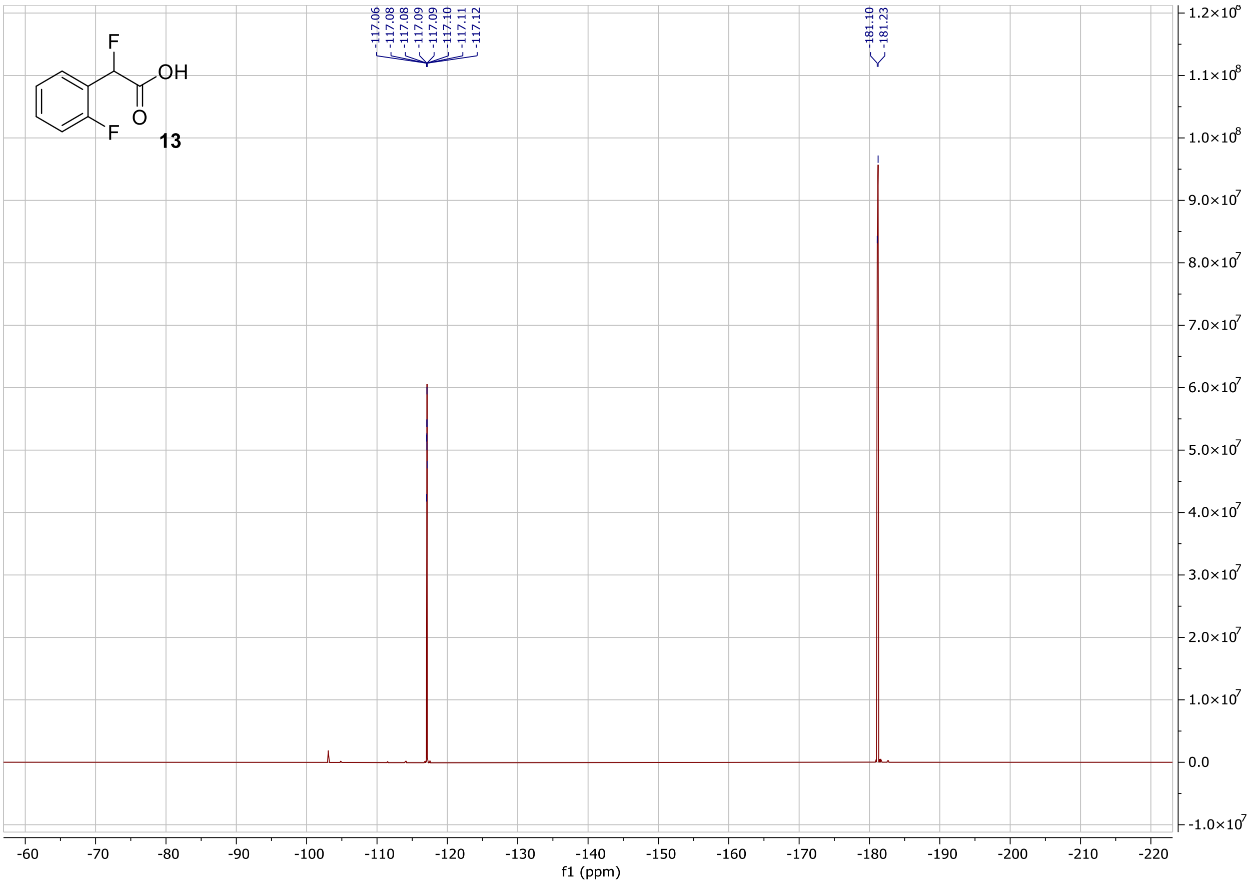

13
-117.06
-117.08
-117.08
-117.09
-117.09
-117.10
-117.11
-117.12
-181.10
-181.23
f1 (ppm)

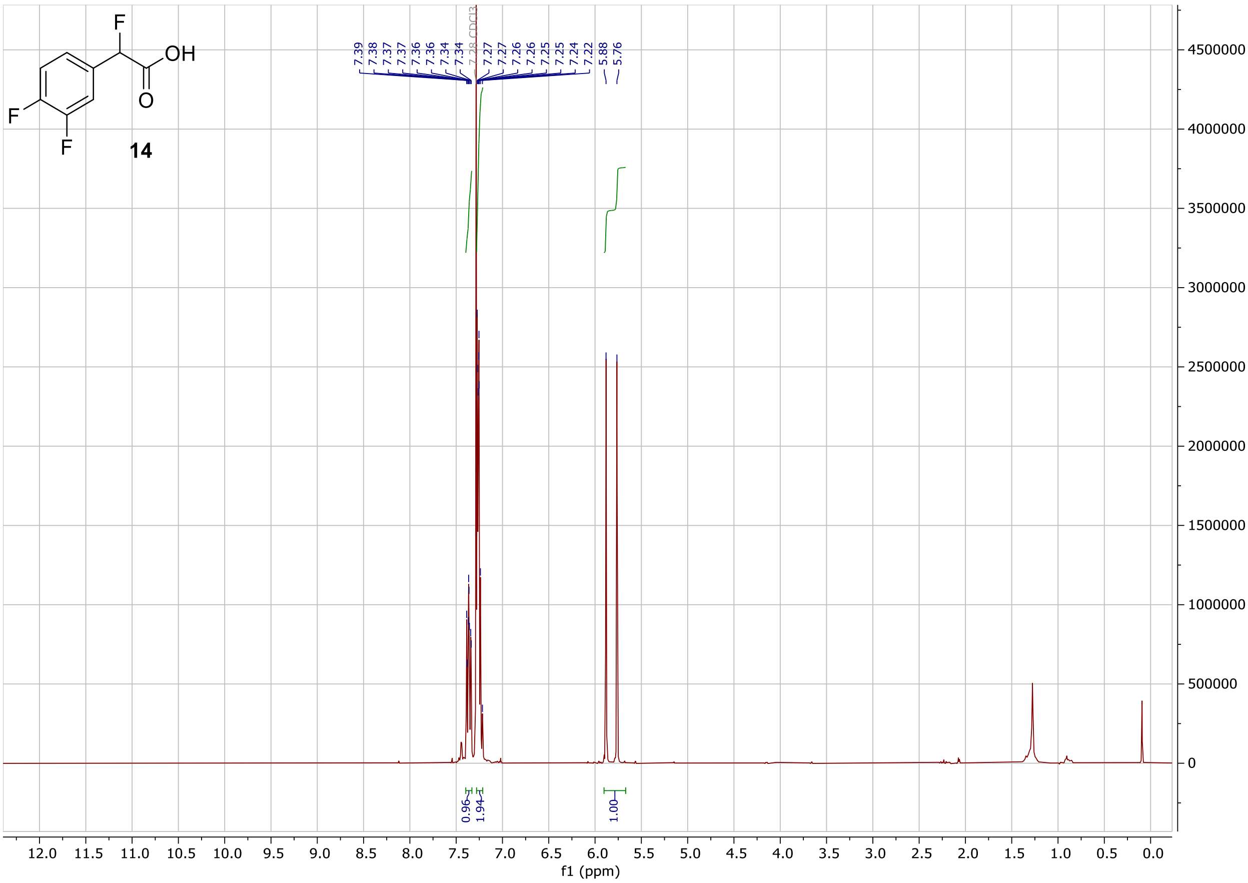
F
OH
O
F
F
14
7.39 7.38 7.37 7.37 7.36 7.36 7.34 7.34
7.28 CDCl3
7.27 7.27 7.26 7.26 7.25 7.25 7.24 7.22
5.88
5.76
0.96
1.94
1.00
f1 (ppm)

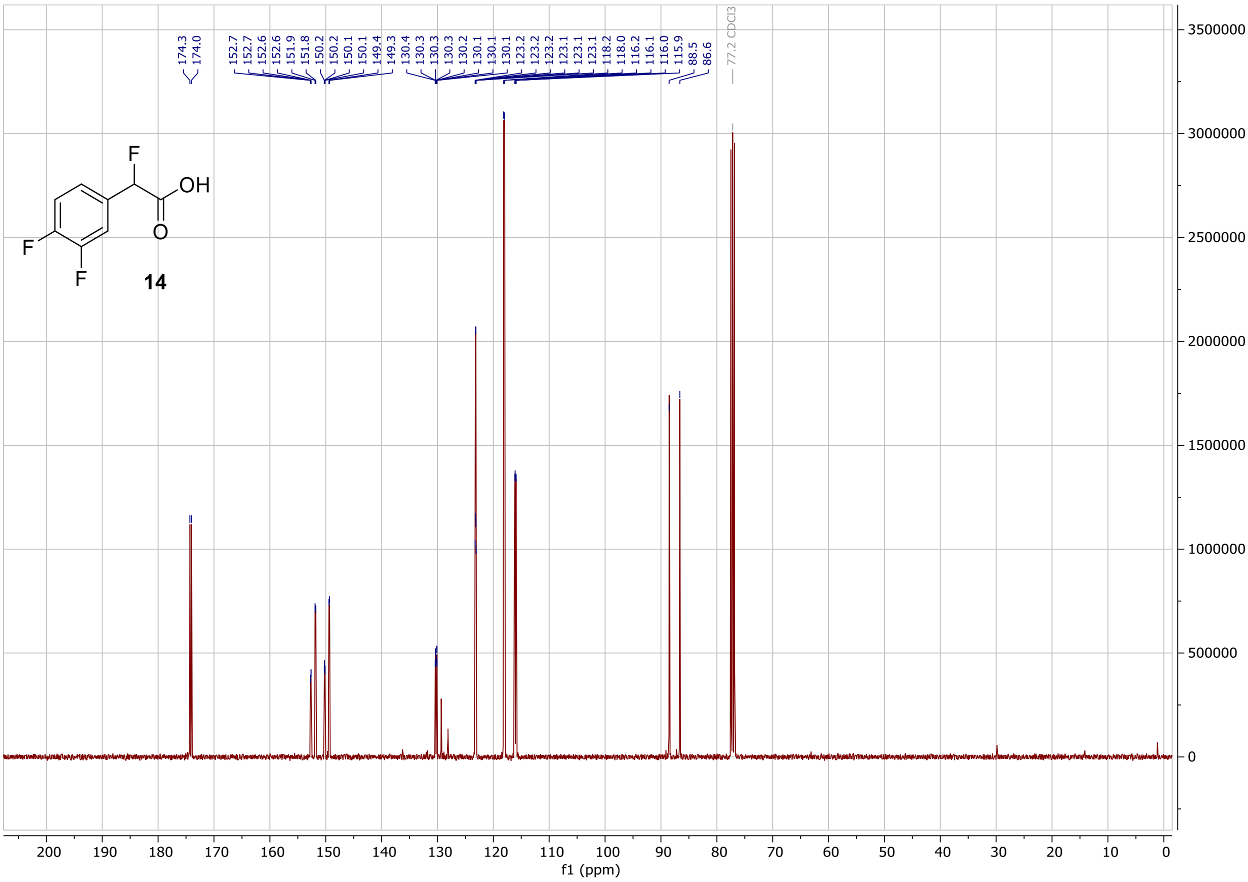
174.3
174.0
152.7 152.7 152.6 152.6 151.9 151.8 150.2 150.2 150.1 150.1 149.4 149.3
130.4 130.3 130.3 130.3 130.2 130.1 130.1 123.2 123.2 123.2 123.2 123.1 123.1 123.1 118.2 118.0 116.2 116.1 116.0 115.9
88.5
86.6
77.2 CDCl3
F
OH
O
F
F
14
f1 (ppm)

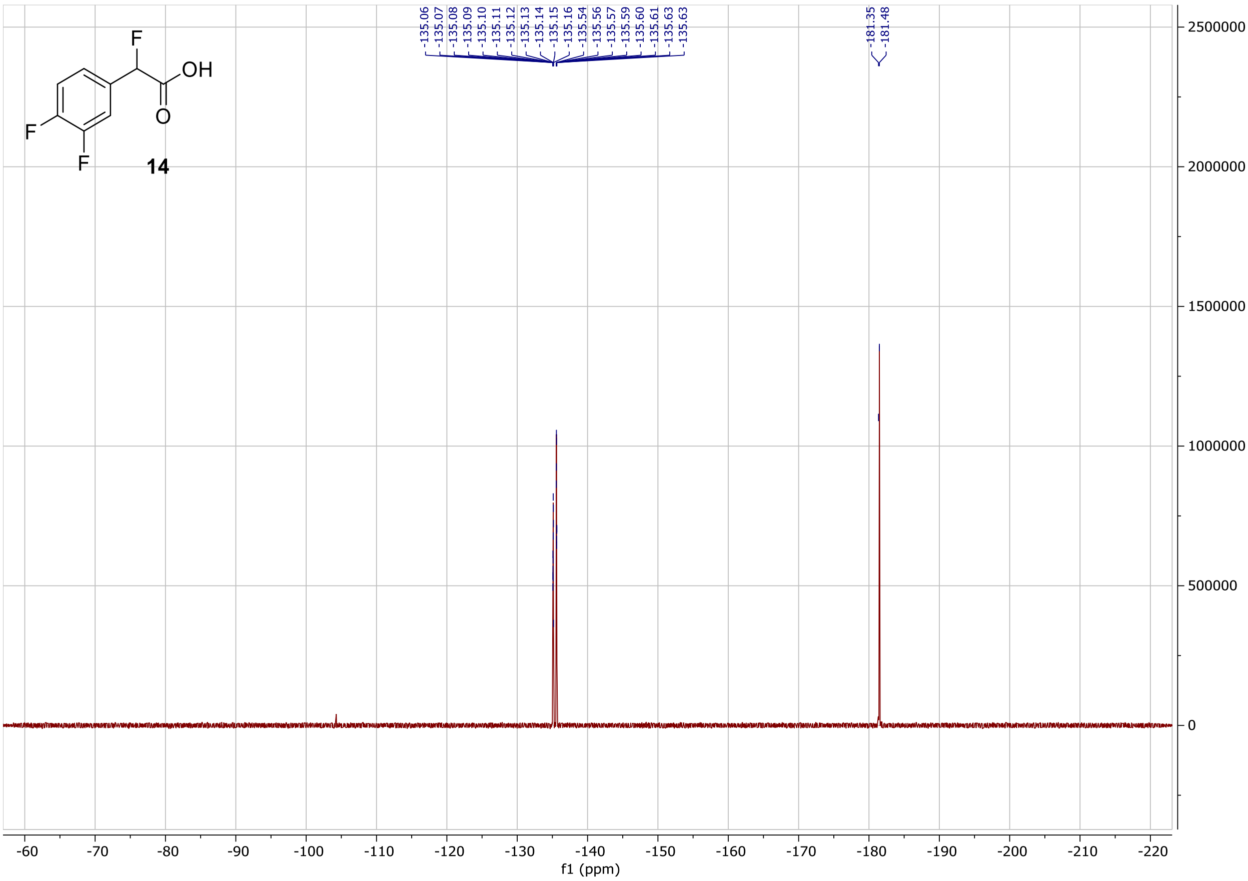

14
f1 (ppm)


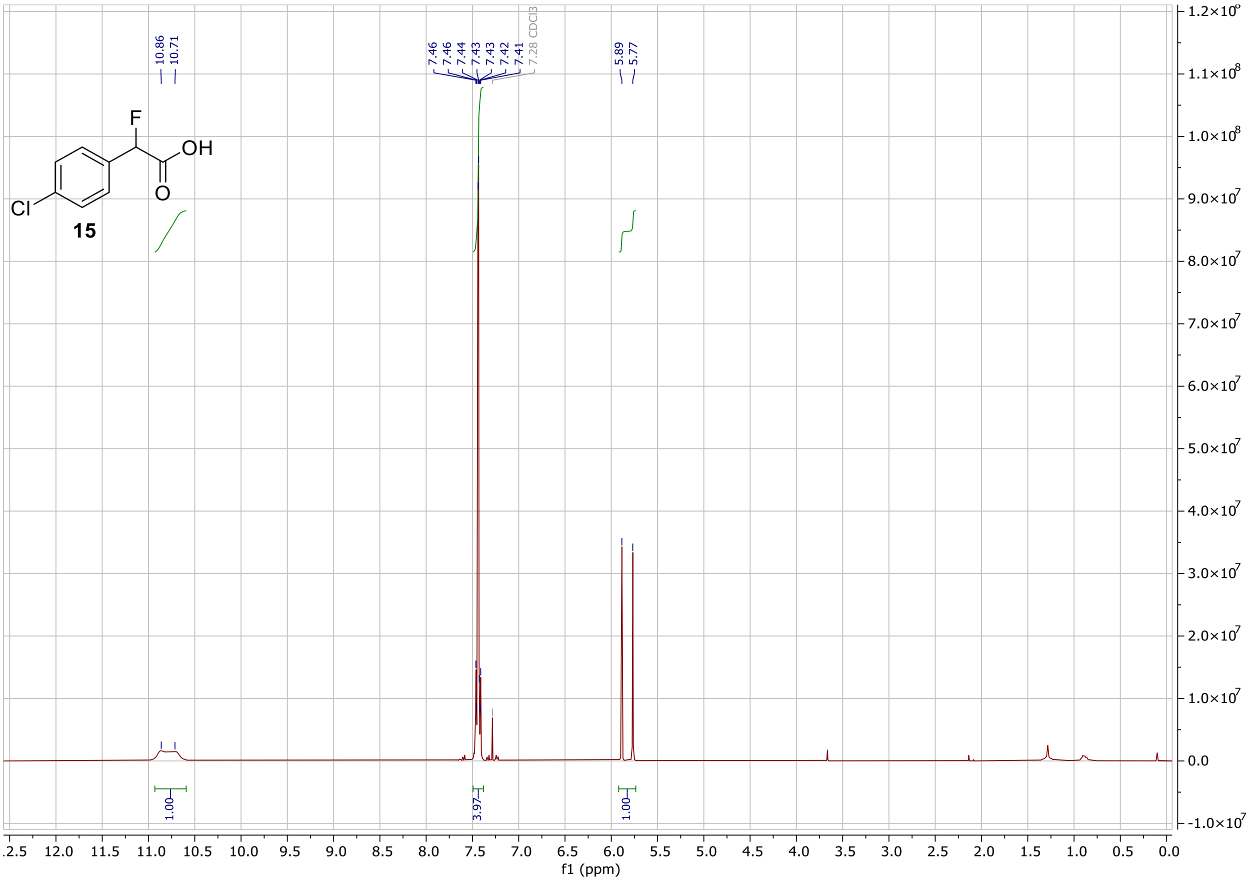

15
f1 (ppm)

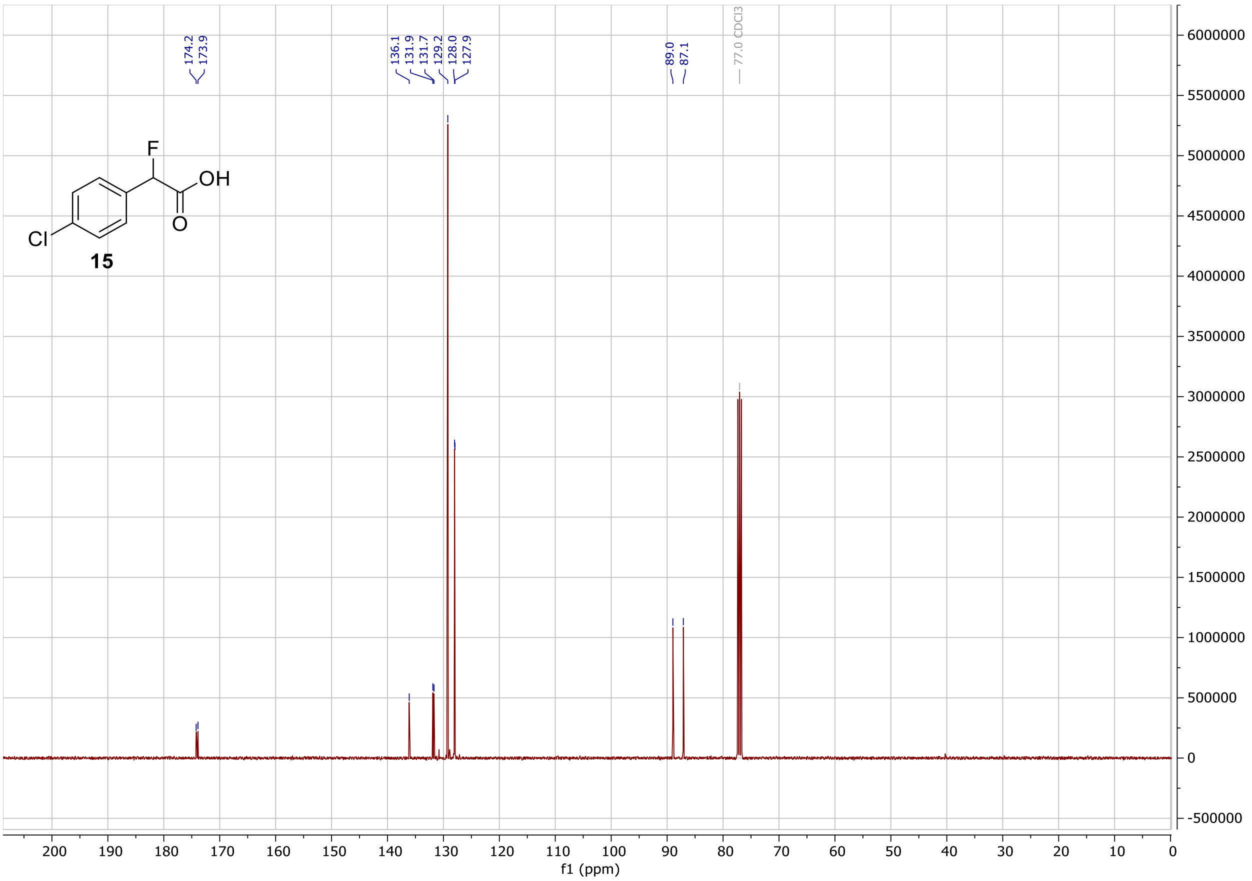

174.2
173.9
136.1
131.9
131.7
129.2
128.0
127.9
89.0
87.1
77.0 CDCl3
F
OH
O
Cl
15
f1 (ppm)


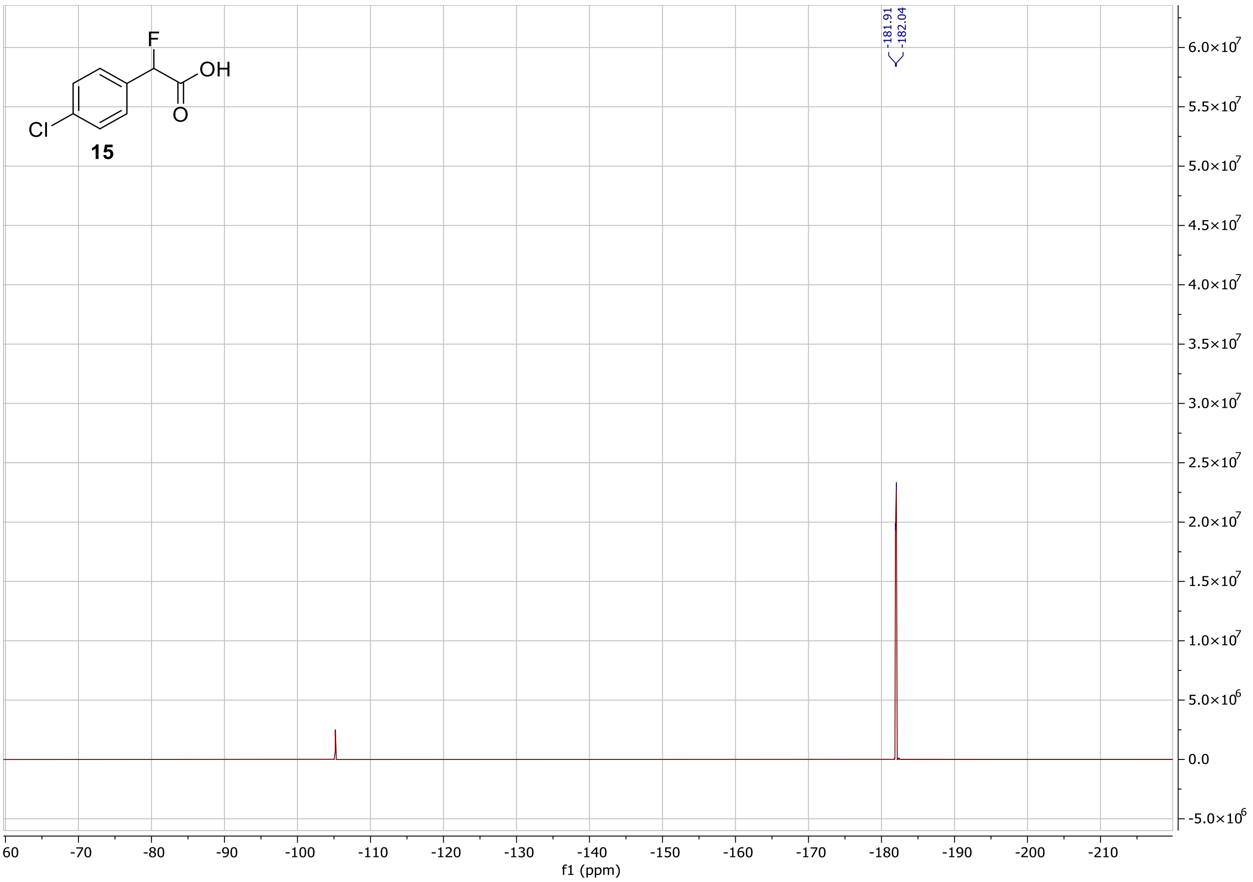

-181.91
-182.04
F
OH
O
Cl
15
f1 (ppm)

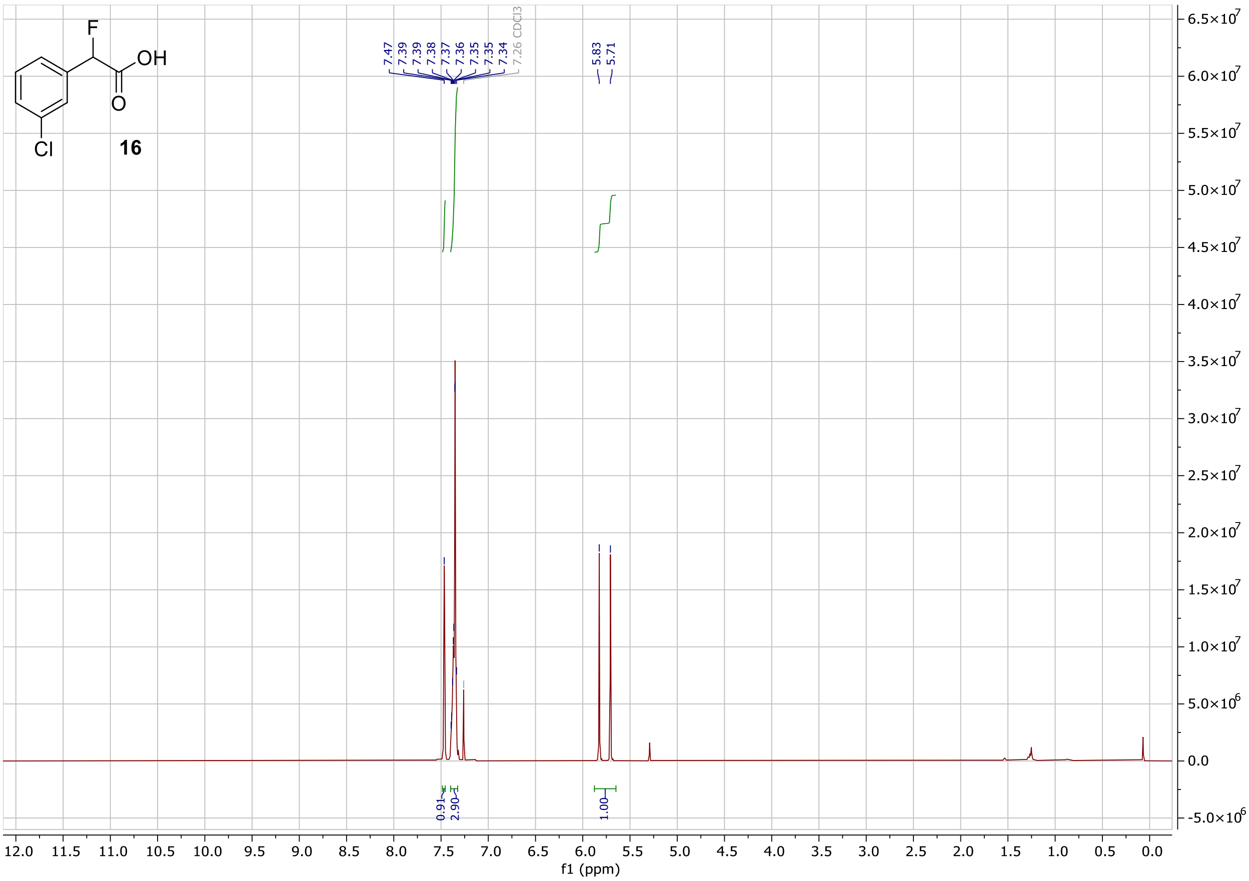
F
OH
O
Cl
16
7.47
7.39
7.39
7.38
7.37
7.36
7.35
7.35
7.34
7.26 CDCl3
5.83
5.71
0.91
2.90
1.00
f1 (ppm)

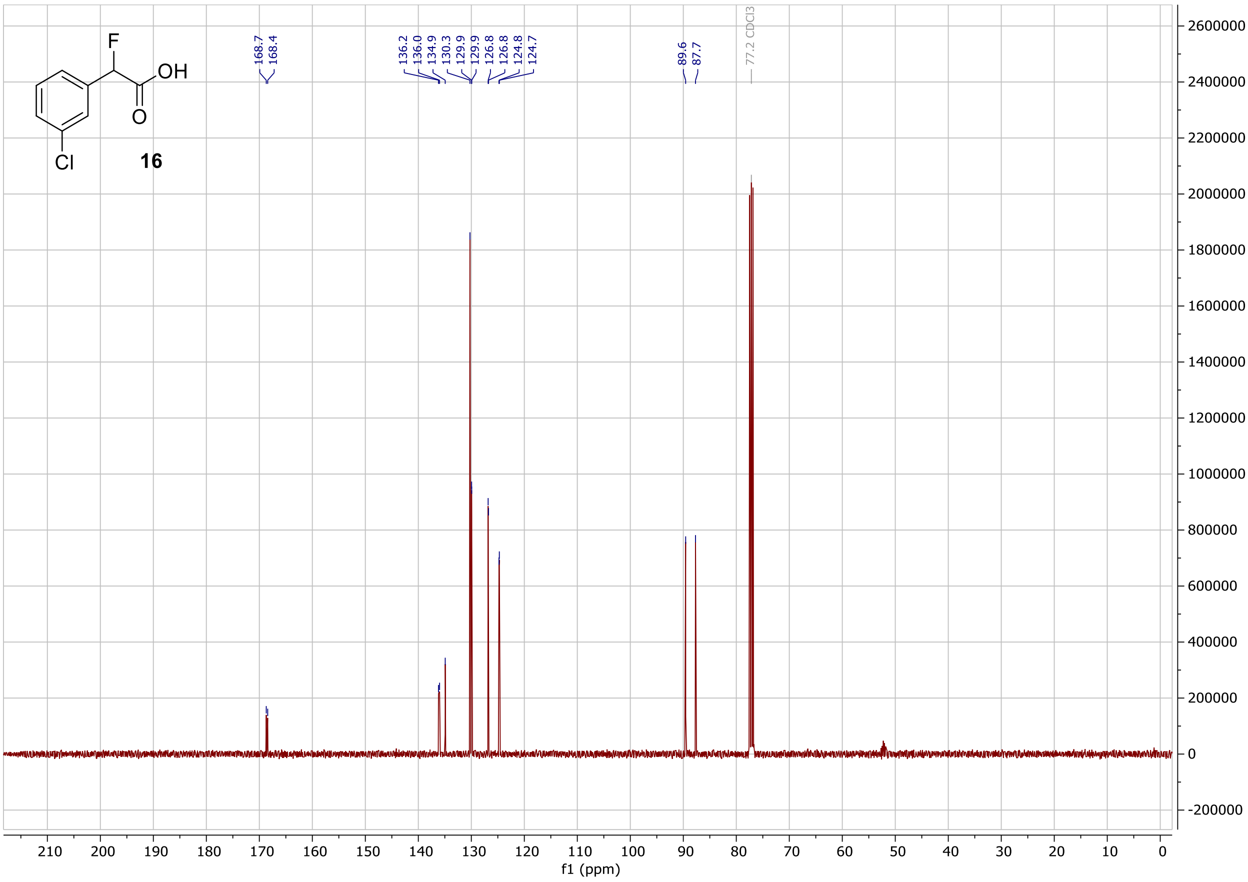
F
OH
O
Cl
16
168.7
168.4
136.2
136.0
134.9
130.3
129.9
129.9
126.8
126.8
124.8
124.7
89.6
87.7
77.2 CDCl3
f1 (ppm)

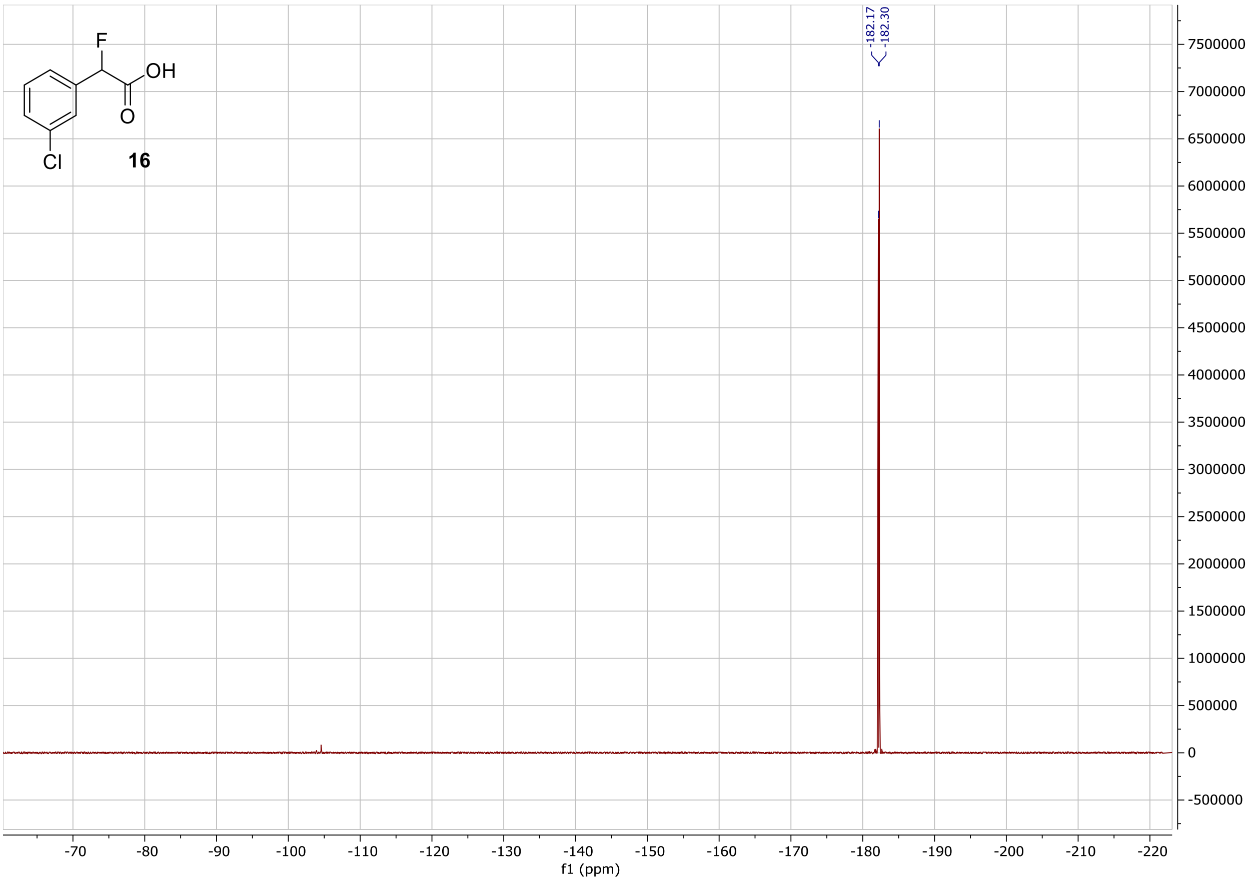

F
OH
O
Cl
16
-182.17
-182.30
7500000
7000000
6500000
6000000
5500000
5000000
4500000
4000000
3500000
3000000
2500000
2000000
1500000
1000000
500000
0
-500000
-70 -80 -90 -100 -110 -120 -130 -140 -150 -160 -170 -180 -190 -200 -210 -220
f1 (ppm)


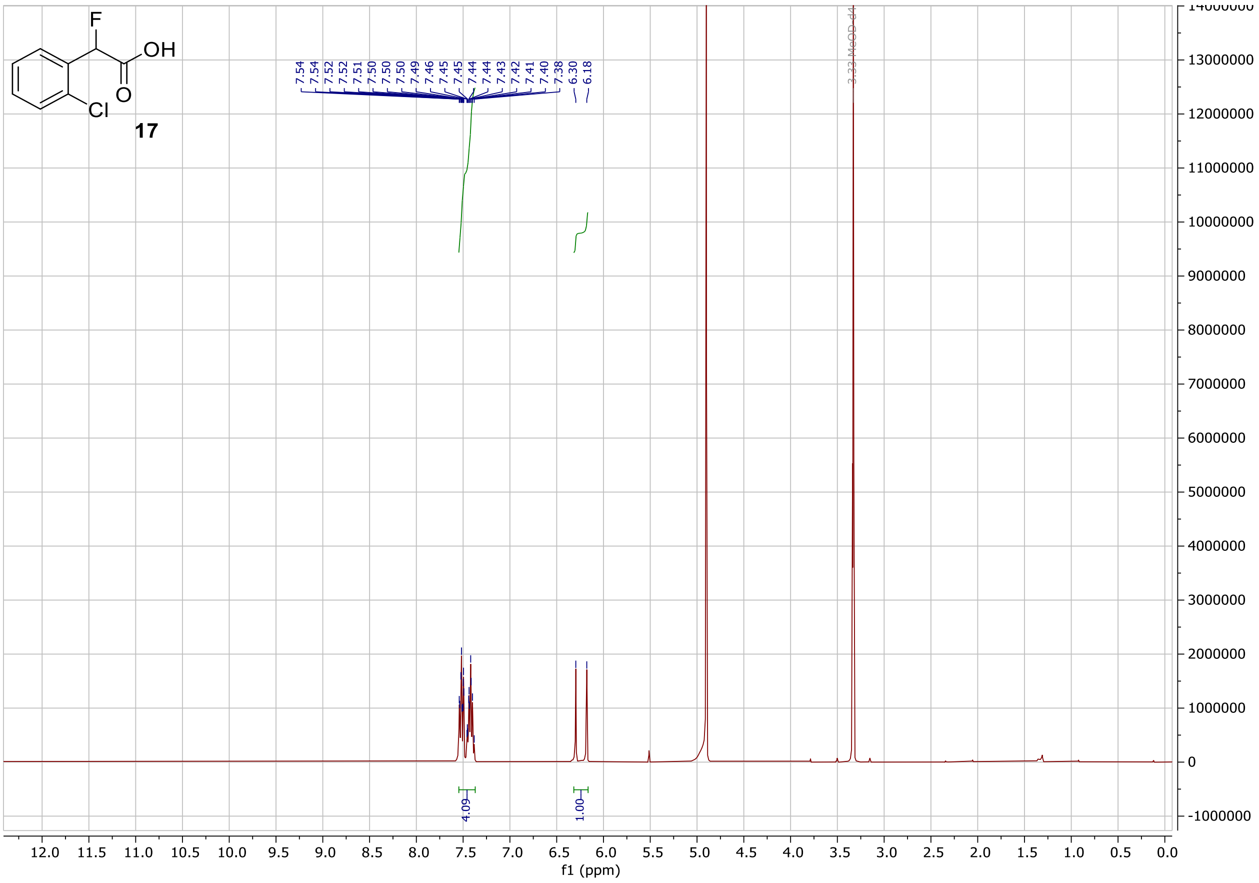

F
OH
O
Cl
17
7.54 7.54 7.52 7.52 7.51 7.50 7.50 7.50 7.49 7.46 7.45 7.45 7.44 7.44 7.43 7.42 7.41 7.40 7.38
6.30
6.18
3.33 MeOD-d4
4.09
1.00
13000000
12000000
11000000
10000000
9000000
8000000
7000000
6000000
5000000
4000000
3000000
2000000
1000000
0
-1000000
12.0 11.5 11.0 10.5 10.0 9.5 9.0 8.5 8.0 7.5 7.0 6.5 6.0 5.5 5.0 4.5 4.0 3.5 3.0 2.5 2.0 1.5 1.0 0.5 0.0
f1 (ppm)

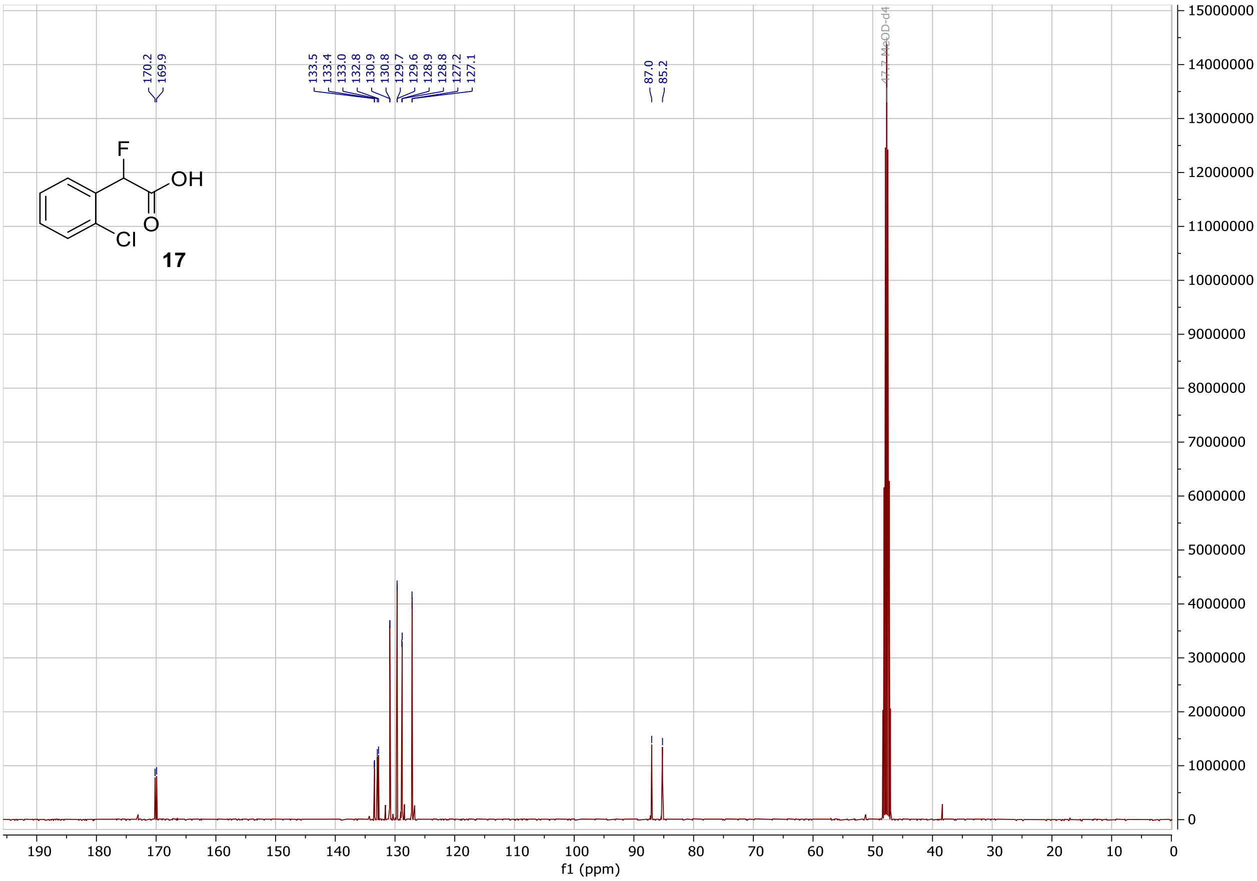
170.2
169.9
133.5
133.4
133.0
132.8
130.9
130.8
129.7
129.6
128.9
128.8
127.2
127.1
87.0
85.2
47.7 MeOD-d4
F
OH
O
Cl
17
f1 (ppm)

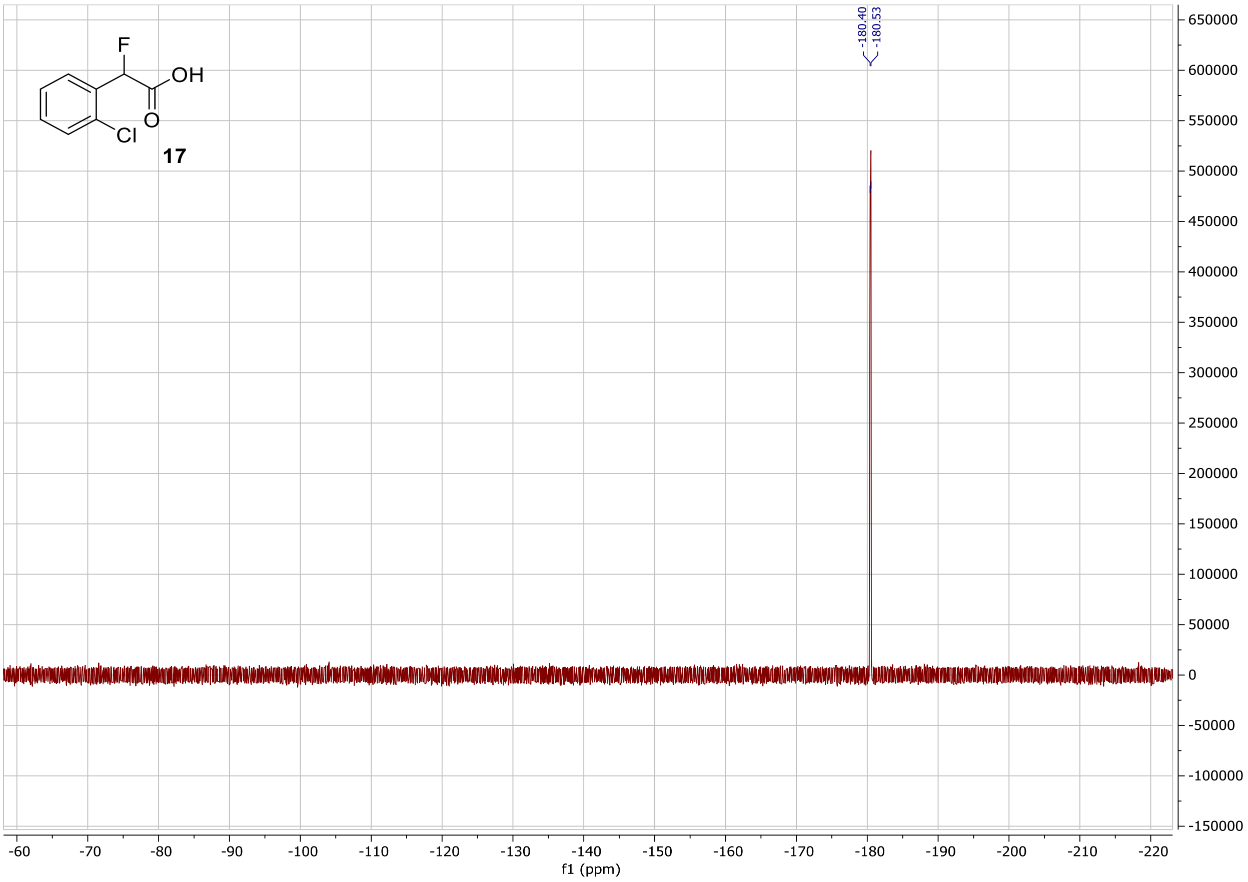
-180.40
-180.53
F
OH
O
Cl
17
f1 (ppm)

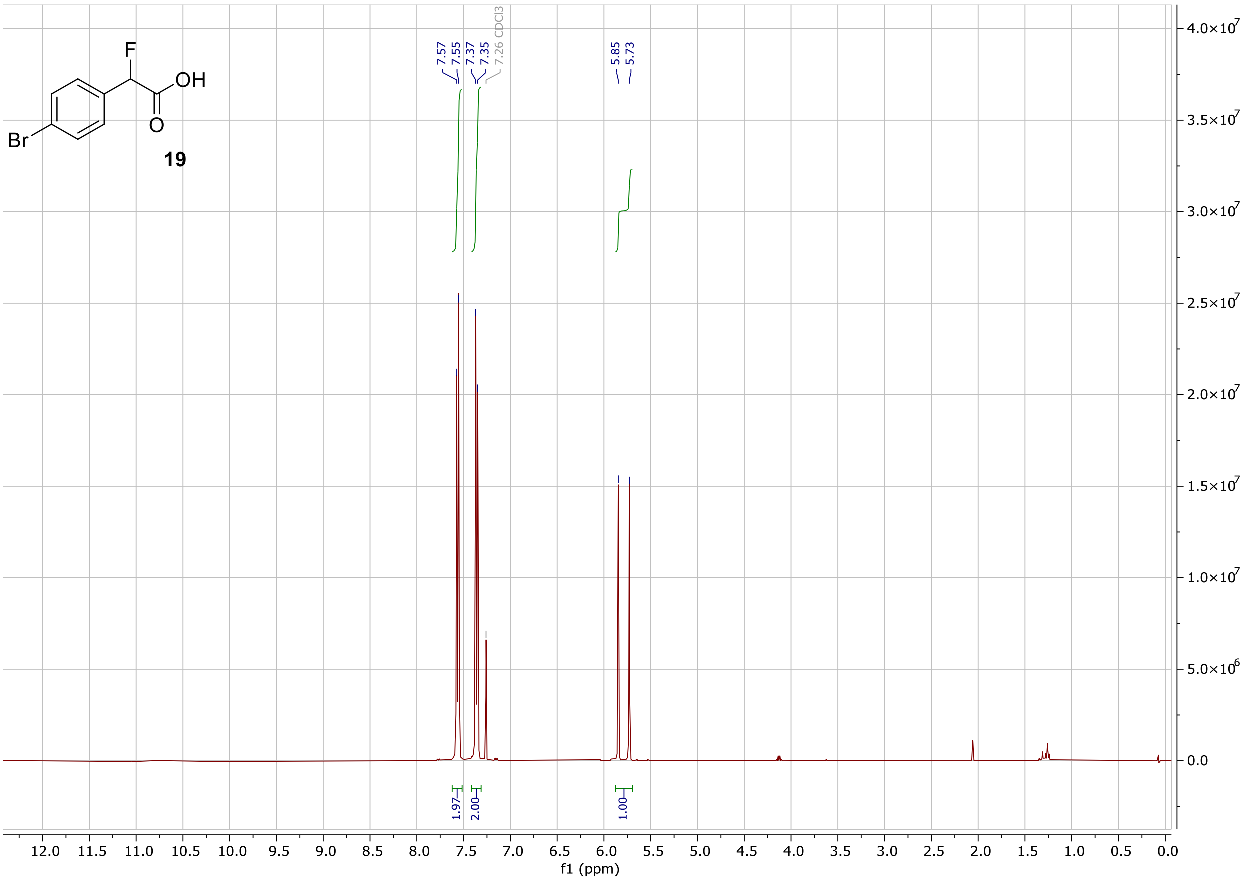

F
OH
O
Br
19
7.57
7.55
7.37
7.35
7.26 CDCl3
5.85
5.73
1.97
2.00
1.00
f1 (ppm)


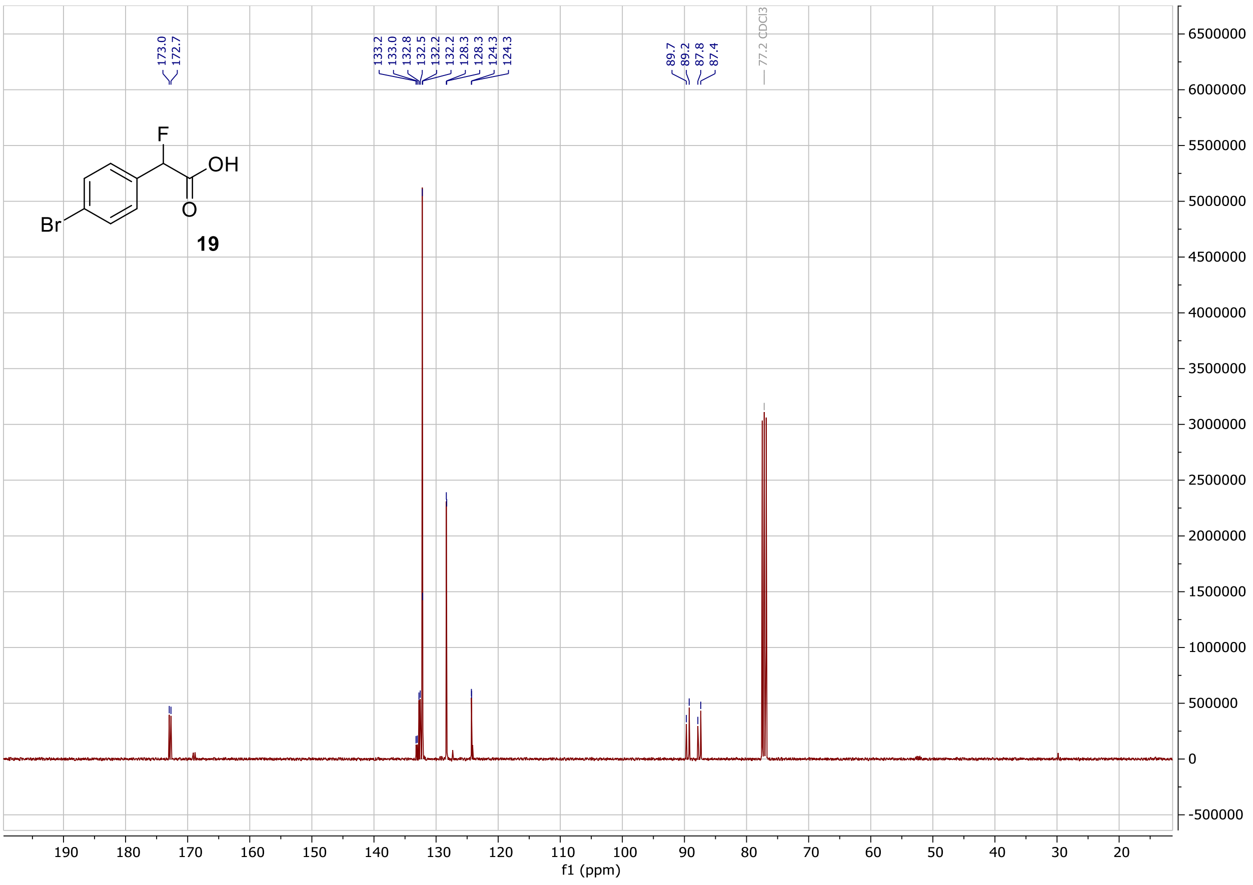

173.0
172.7
133.2
133.0
132.8
132.5
132.2
132.2
128.3
128.3
124.3
124.3
89.7
89.2
87.8
87.4
77.2 CDCl3
F
OH
O
Br
19
f1 (ppm)

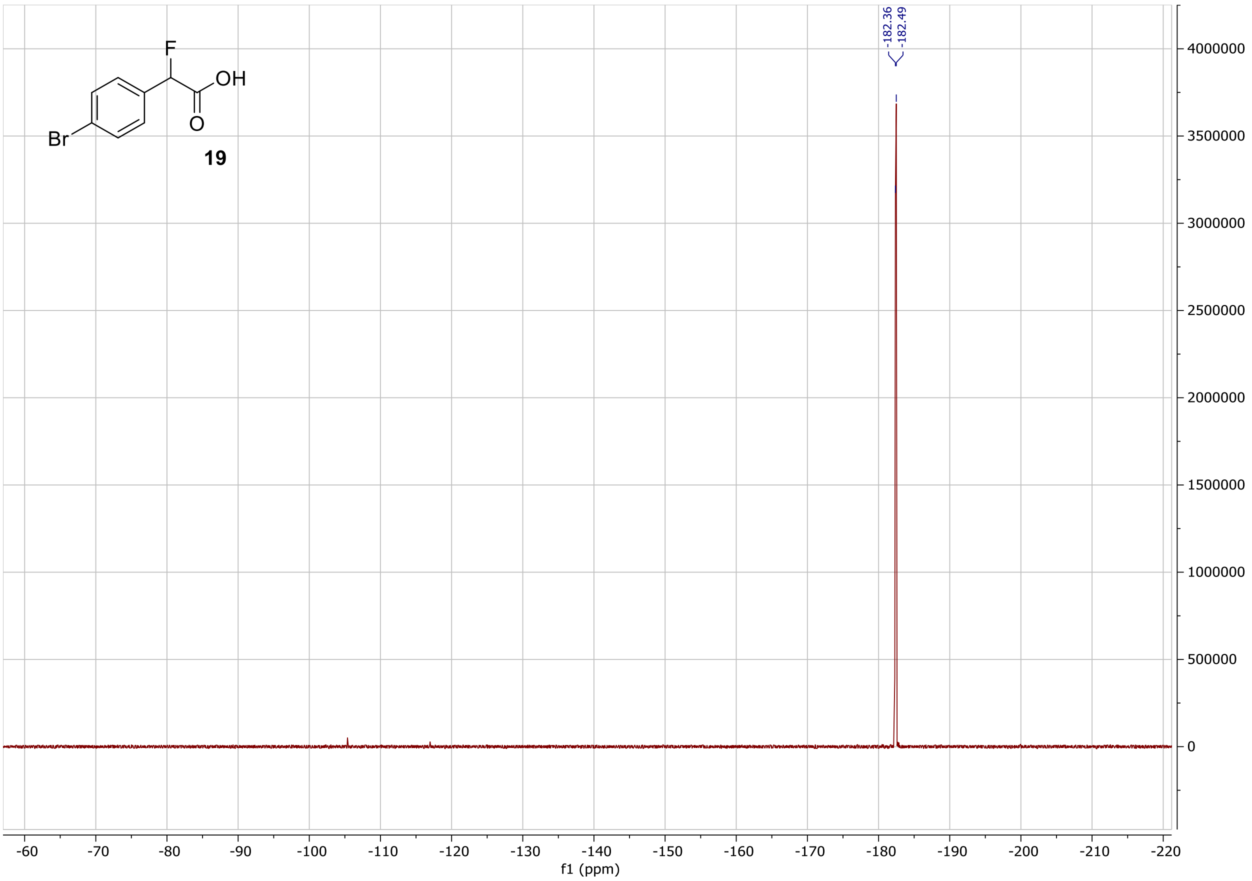
19
-182.36
-182.49
f1 (ppm)

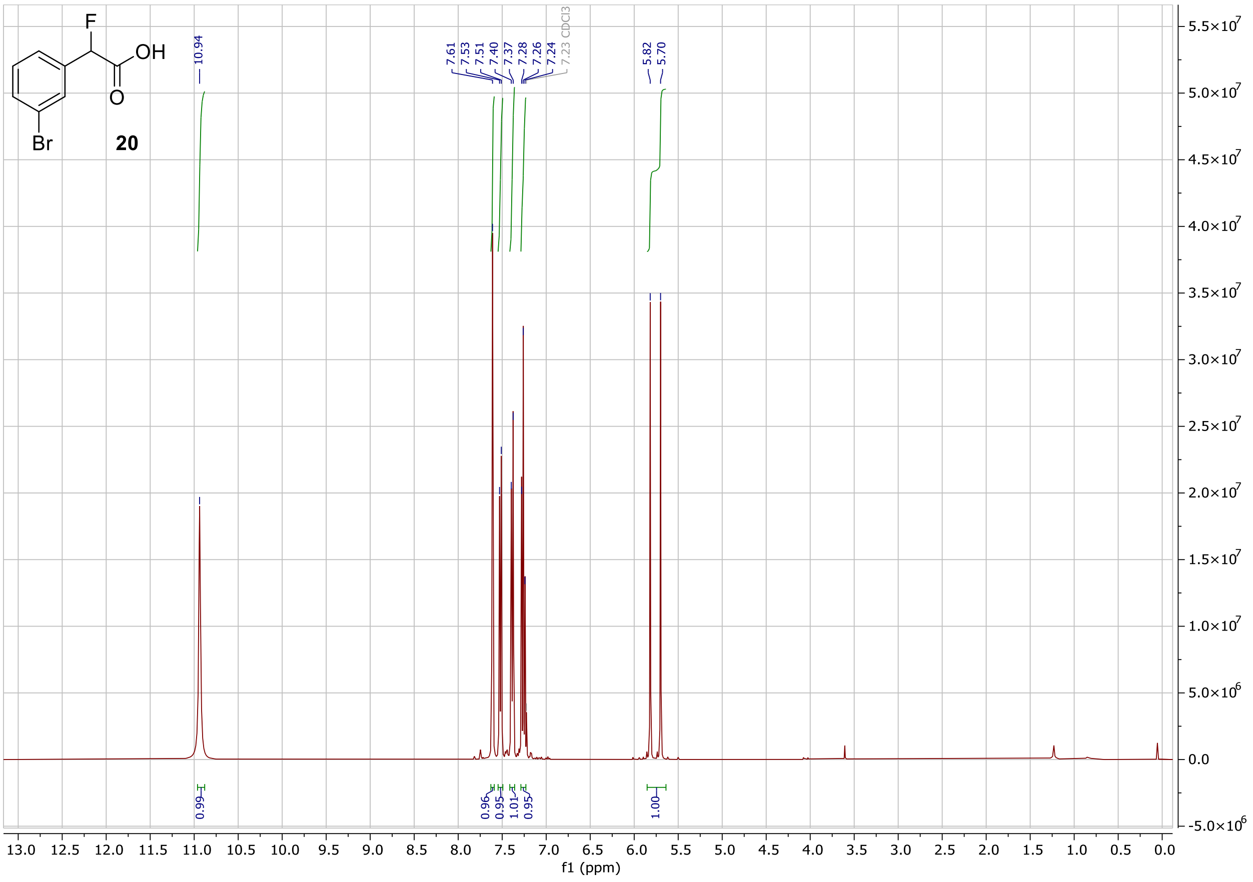
20
10.94
7.61
7.53
7.51
7.40
7.37
7.28
7.26
7.24
7.23 CDCl3
5.82
5.70
0.99
0.96
0.95
1.01
0.95
1.00
f1 (ppm)

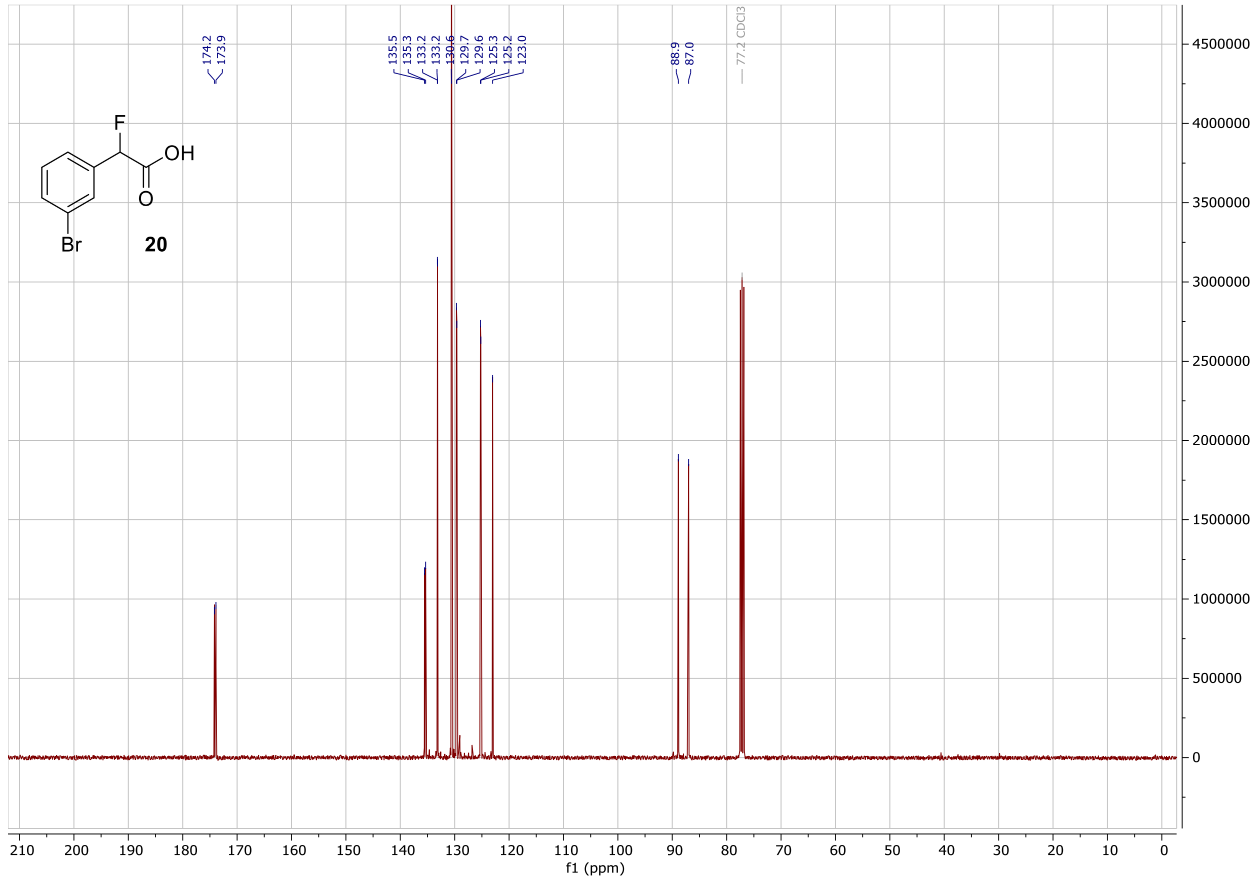
174.2
173.9
135.5
135.3
133.2
133.2
130.6
129.7
129.6
125.3
125.2
123.0
88.9
87.0
77.2 CDCl3
F
OH
O
Br
20
f1 (ppm)

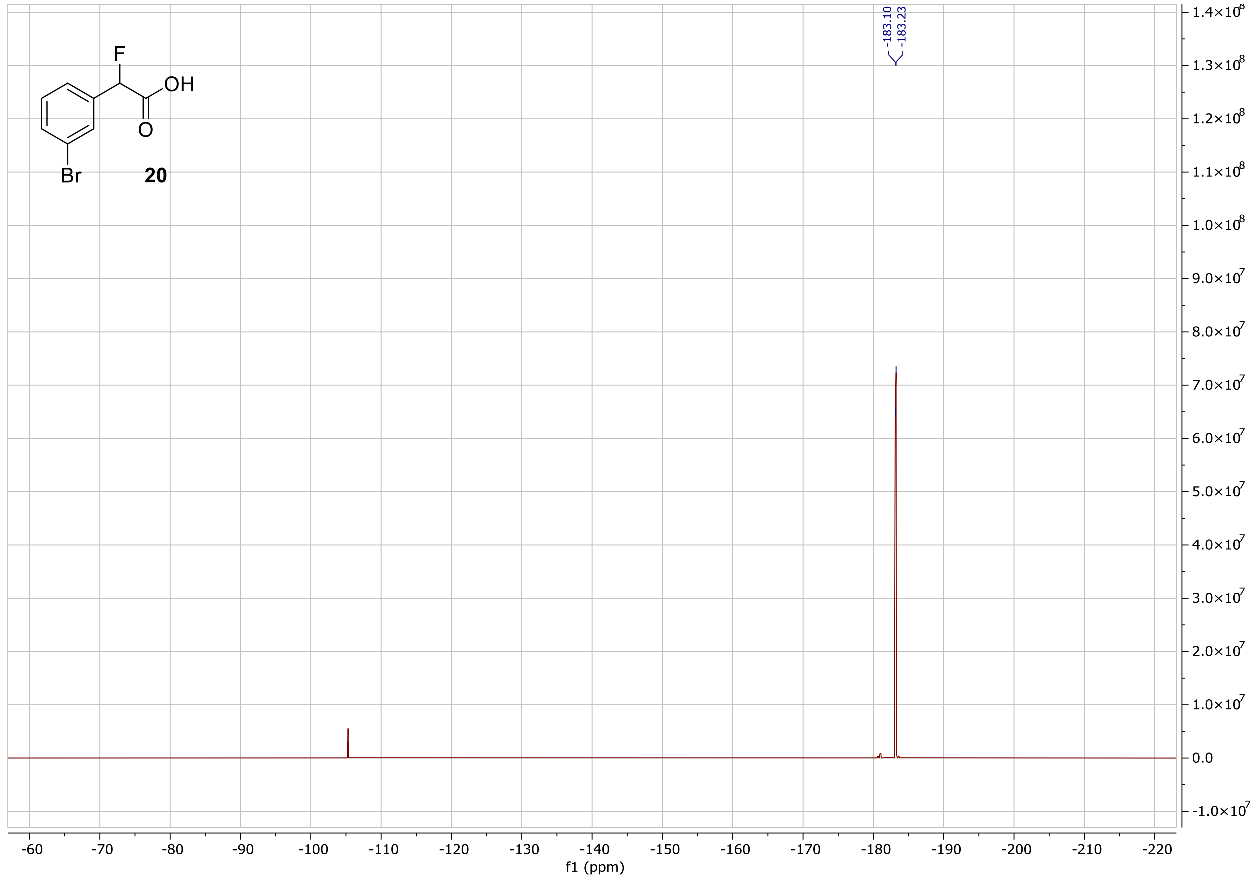
-183.10
-183.23
F
OH
O
Br
20
f1 (ppm)

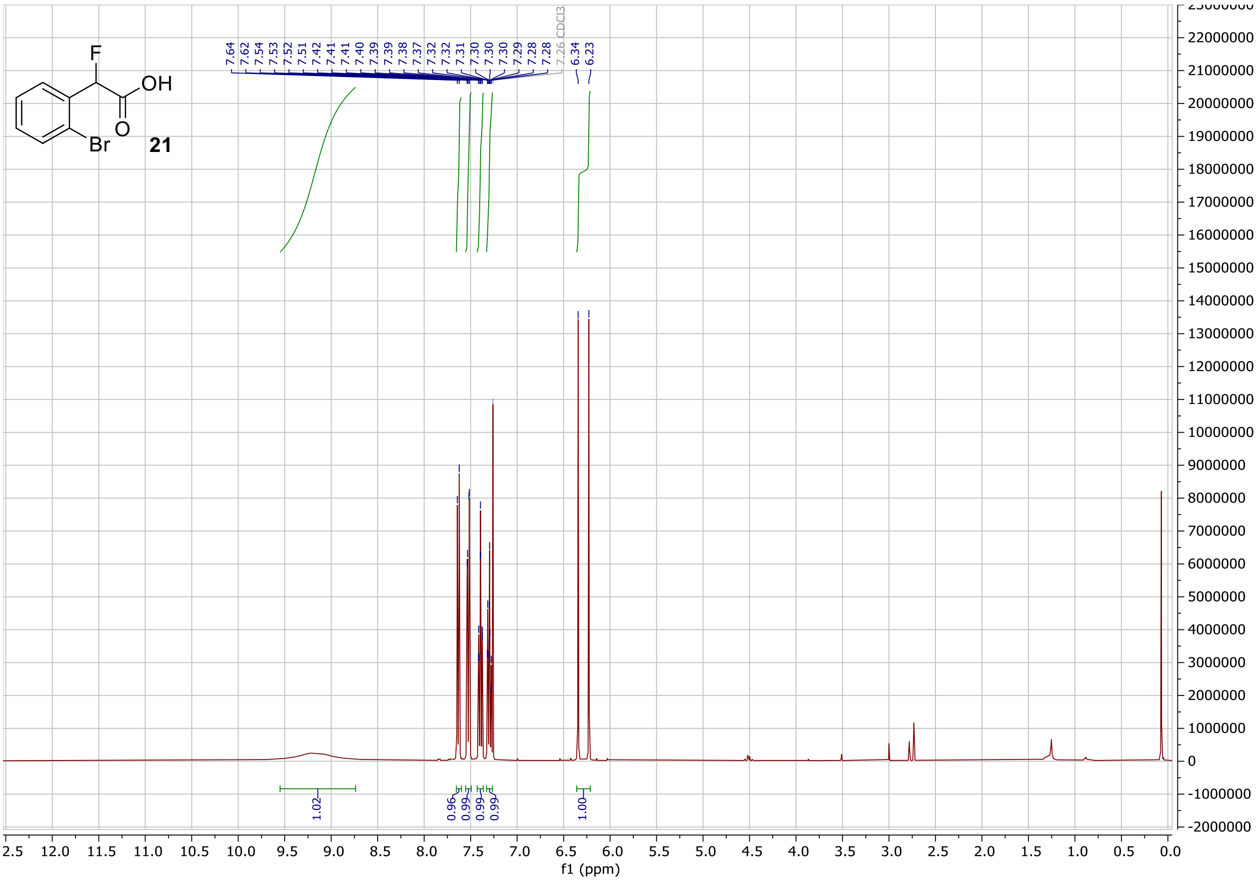

F
OH
O
Br
21
7.64 7.62 7.54 7.53 7.52 7.51 7.42 7.41 7.40 7.39 7.39 7.38 7.37 7.32 7.32 7.31 7.30 7.30 7.30 7.29 7.28 7.28
7.26 CDCl3
6.34
6.23
1.02
0.96 0.99 0.99 0.99
1.00
f1 (ppm)


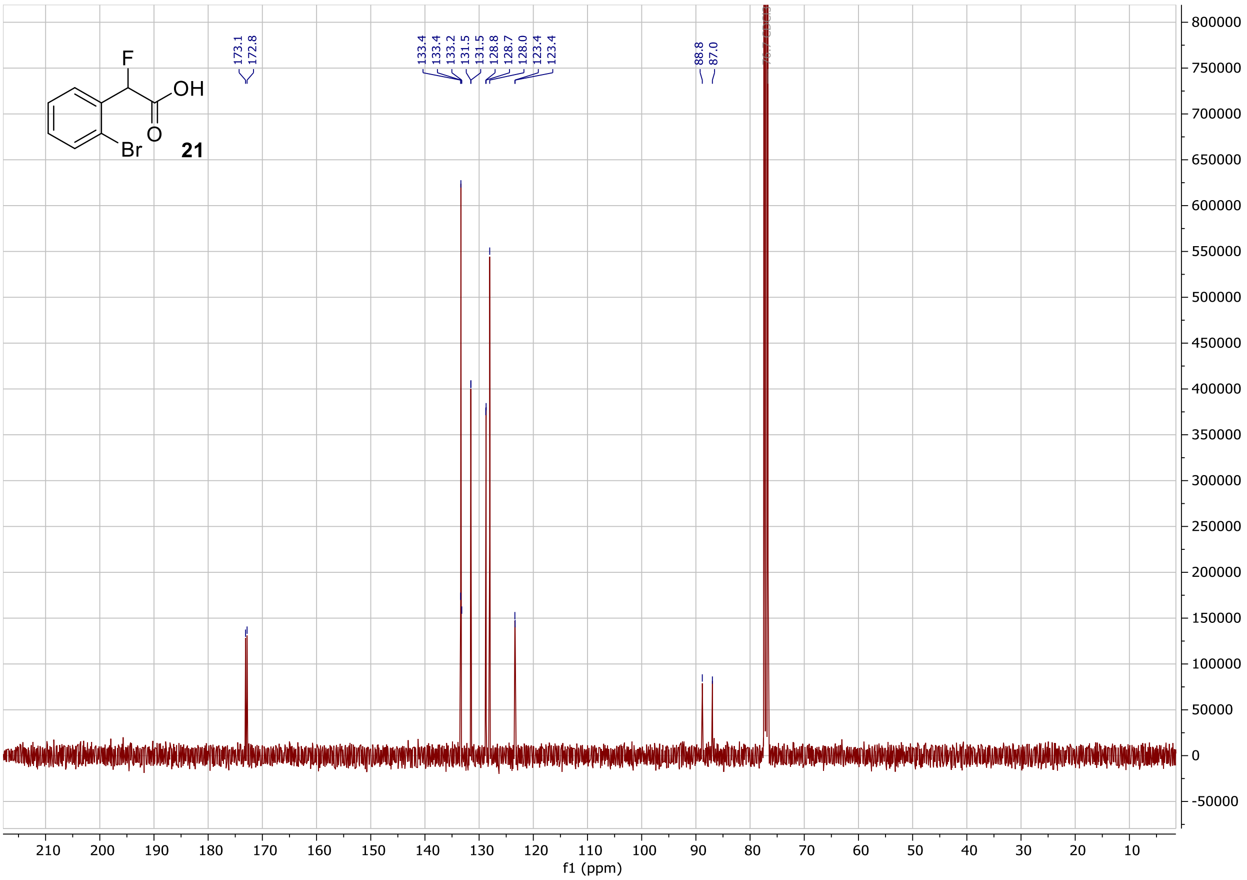

F
OH
O
Br
21
173.1 172.8
133.4 133.4 133.2 131.5 128.8 128.7 128.0 123.4 123.4
88.8 87.0
f1 (ppm)

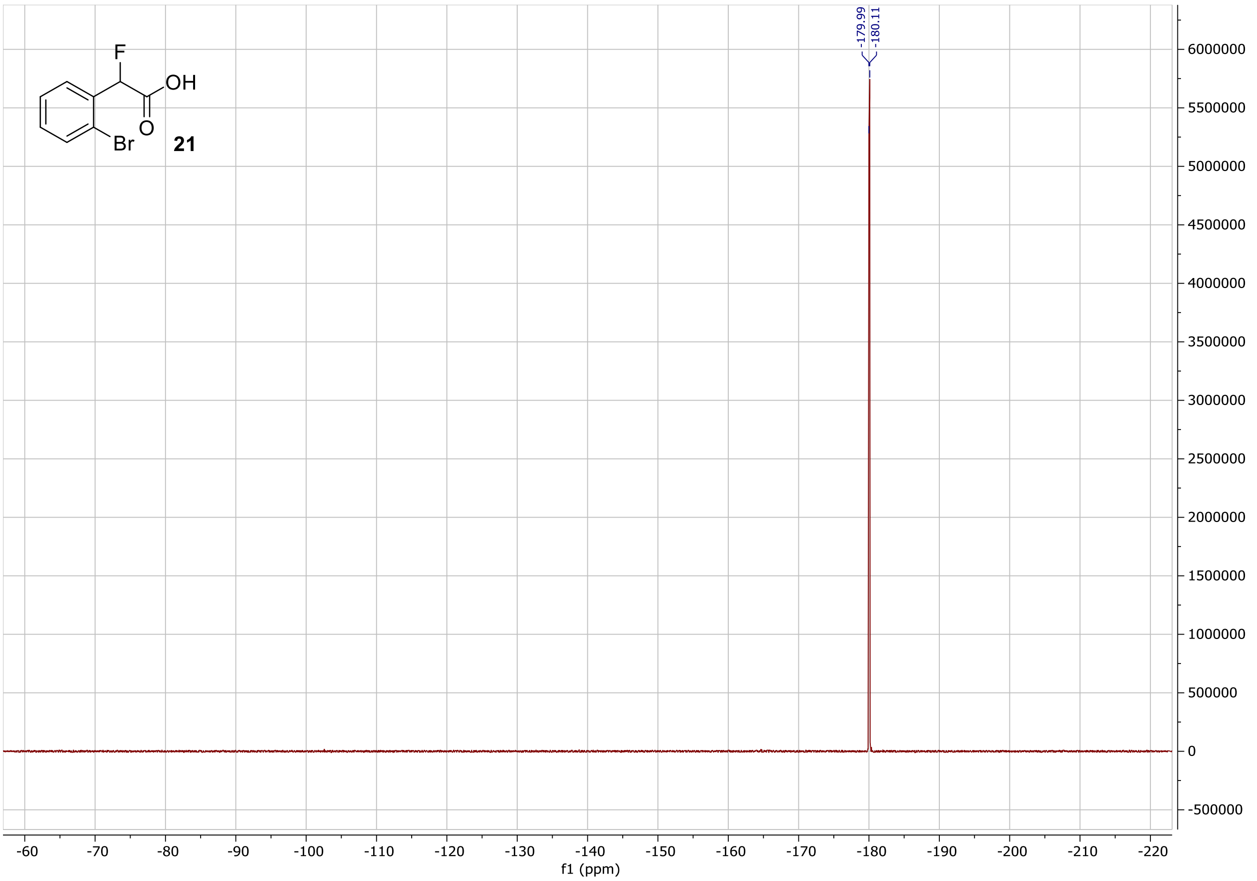

21
-179.99
-180.11
f1 (ppm)


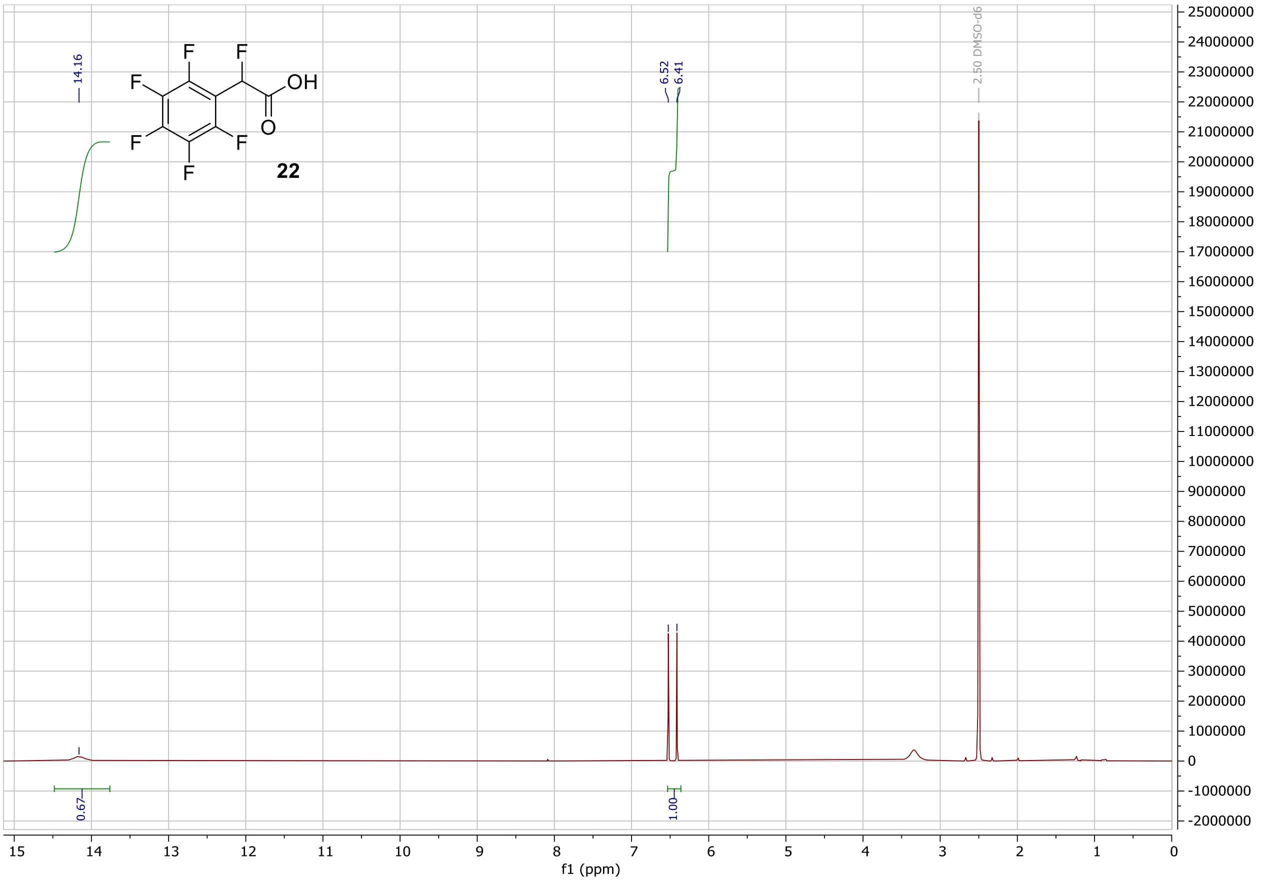

22
14.16
6.52
6.41
2.50 DMSO-d6
0.67
1.00
f1 (ppm)

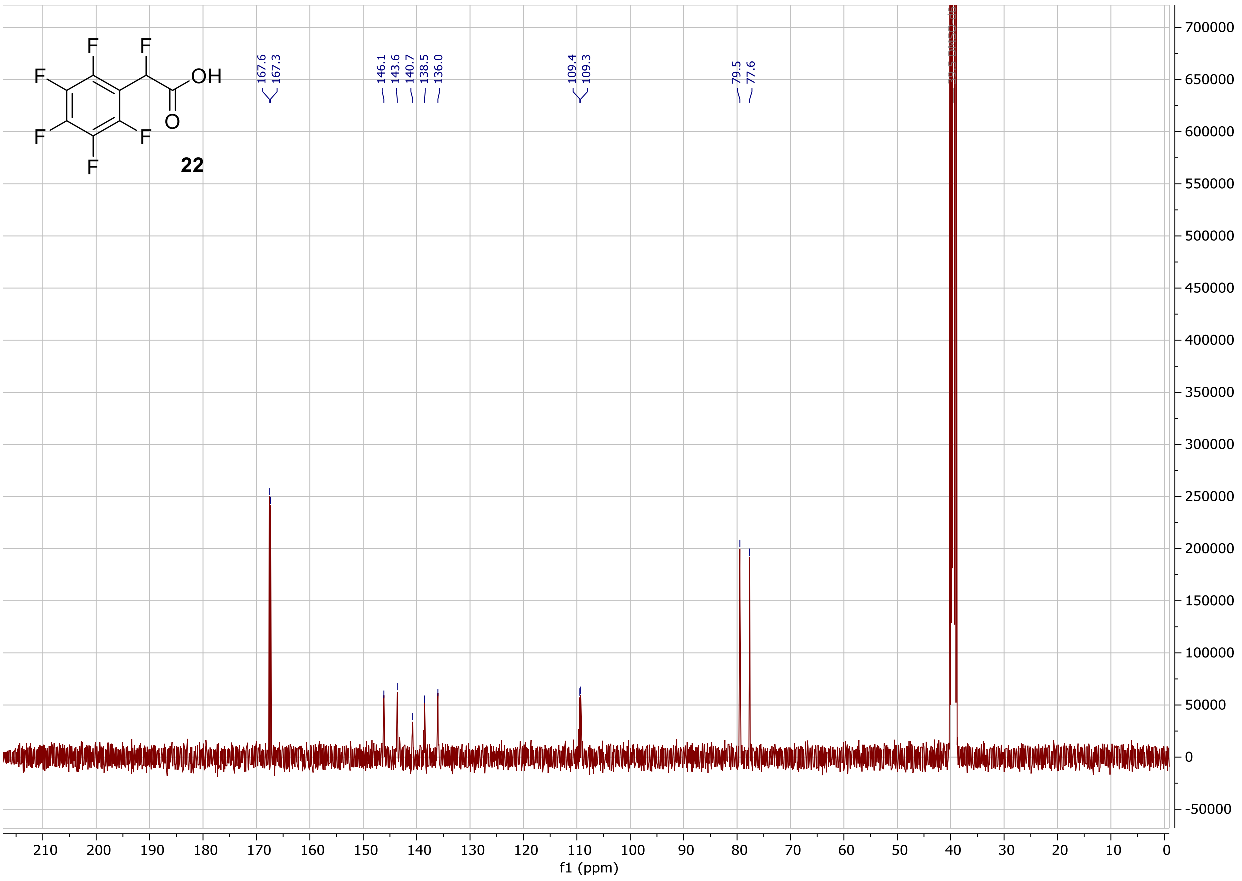

OH
O
F
22
167.6
167.3
146.1
143.6
140.7
138.5
136.0
109.4
109.3
79.5
77.6
f1 (ppm)


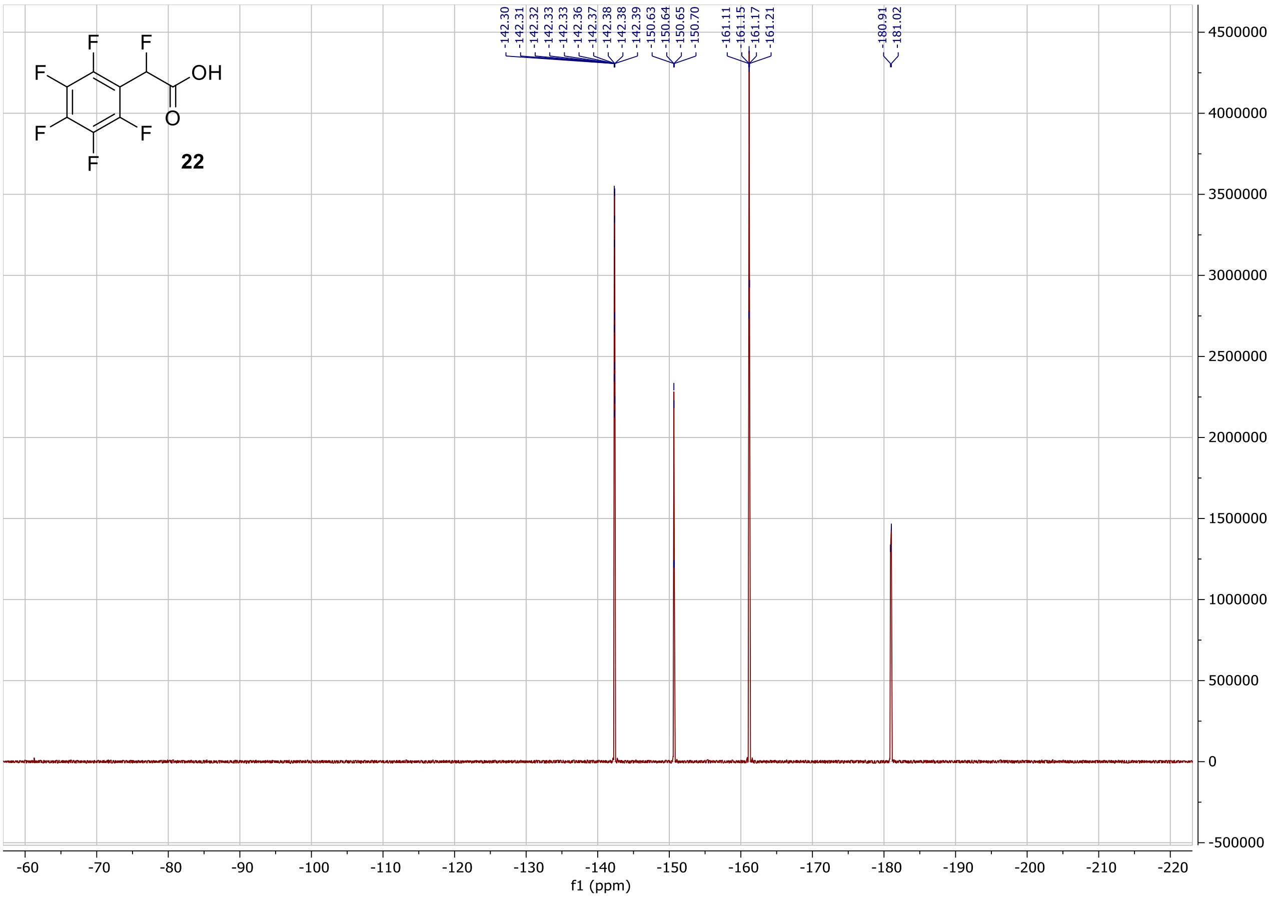

OH
O
F
22
-142.30
-142.31
-142.32
-142.33
-142.33
-142.36
-142.37
-142.38
-142.38
-142.39
-150.63
-150.64
-150.65
-150.70
-161.11
-161.15
-161.17
-161.21
-180.91
-181.02
f1 (ppm)

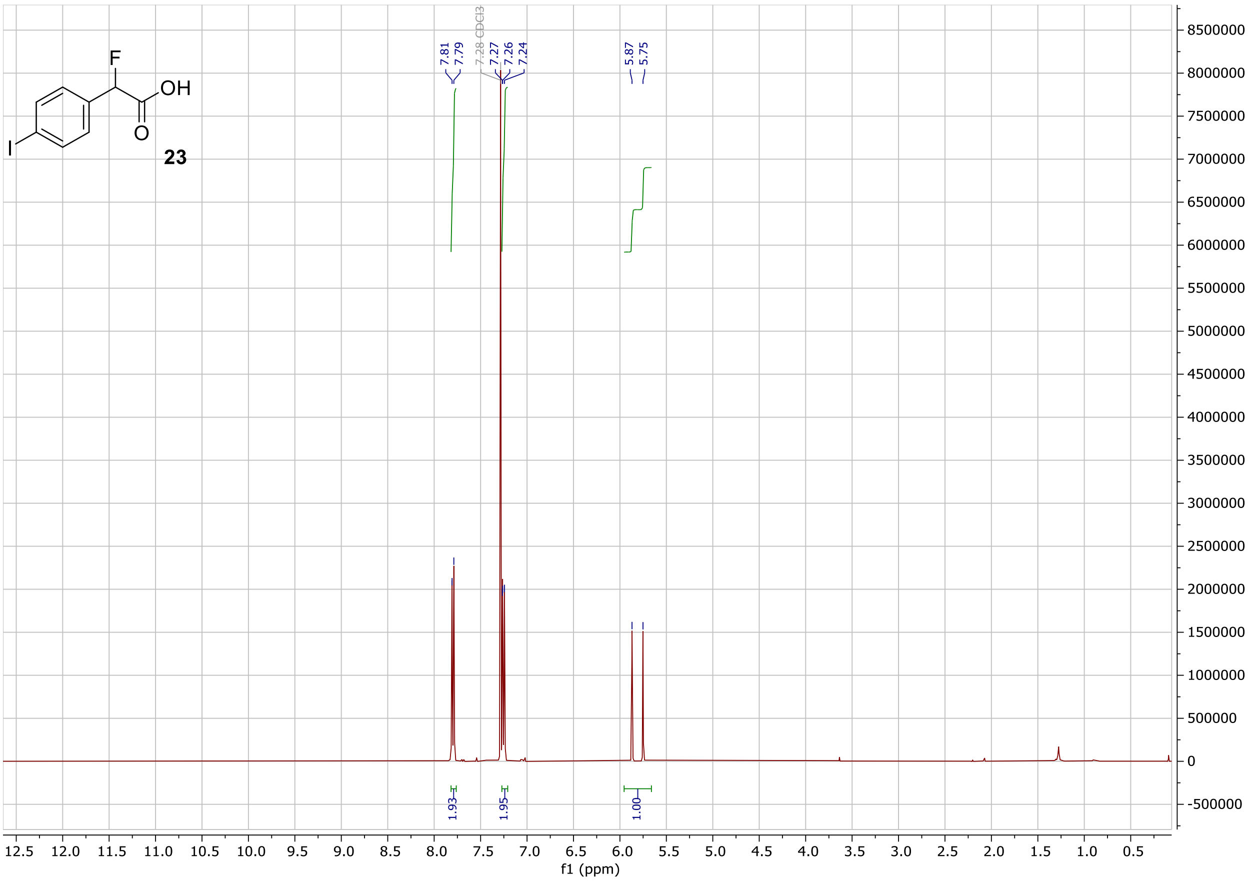
F
OH
O
I
23
7.81
7.79
7.28 CDCl3
7.27
7.26
7.24
5.87
5.75
1.93
1.95
1.00
f1 (ppm)

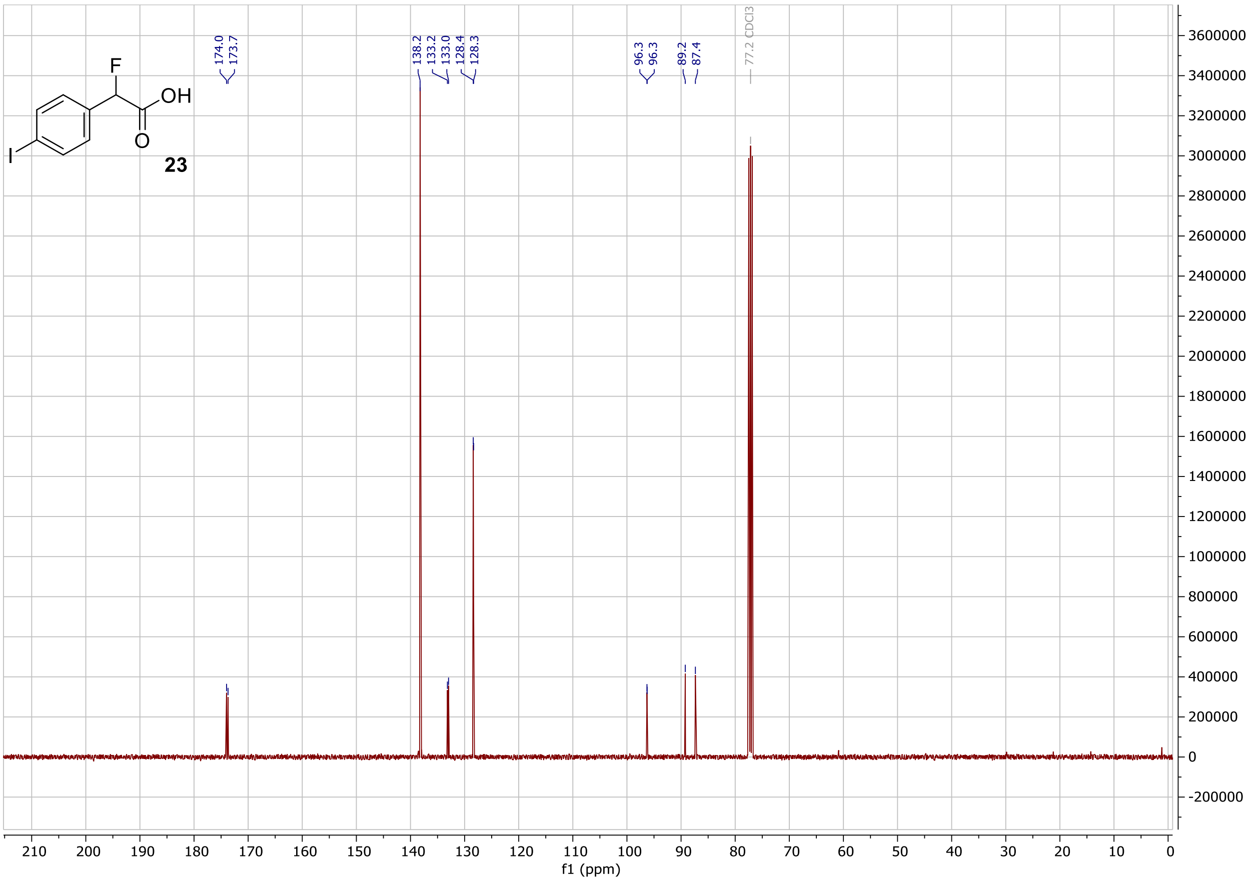
F
OH
O
I
23
174.0
173.7
138.2
133.2
133.0
128.4
128.3
96.3
96.3
89.2
87.4
77.2 CDCl3
f1 (ppm)

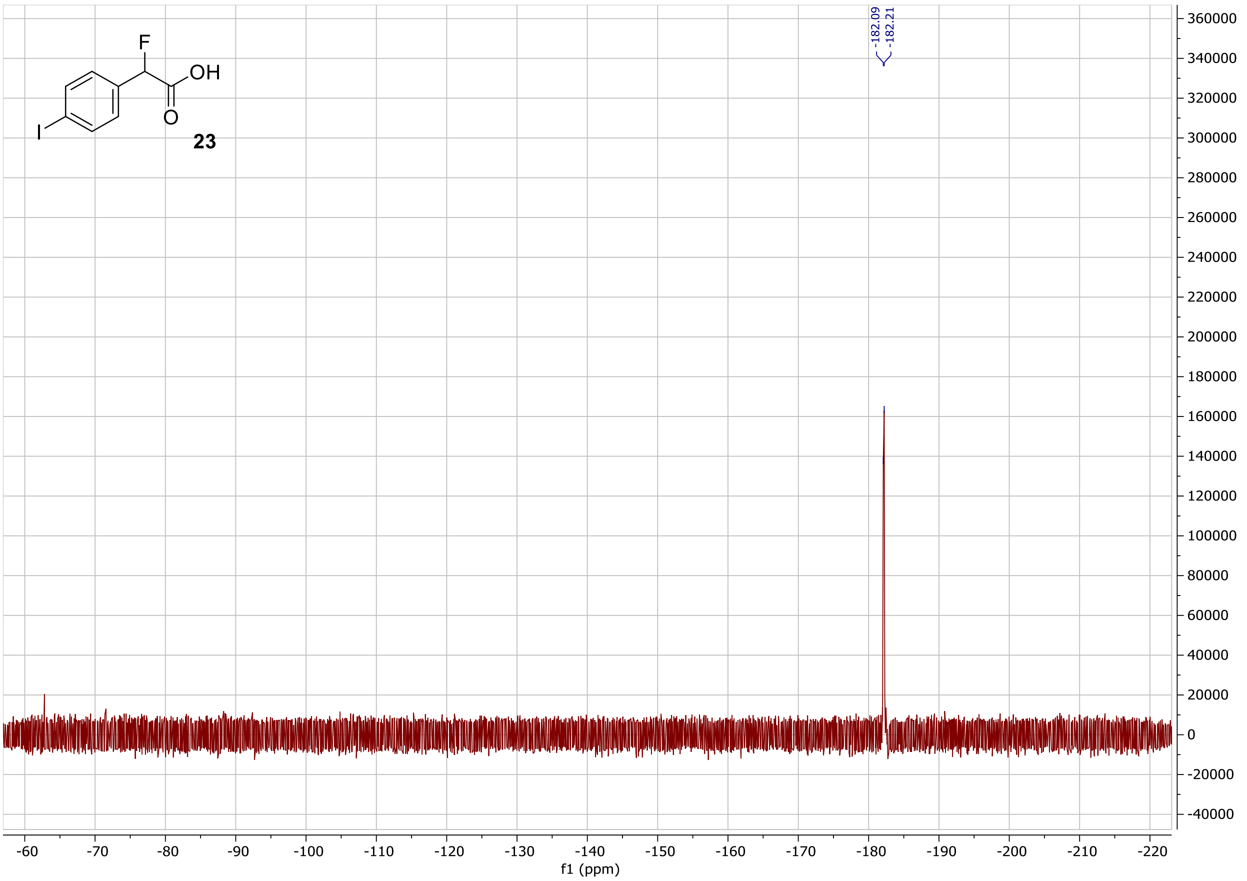
F
OH
O
I
23
-182.09
-182.21
360000
340000
320000
300000
280000
260000
240000
220000
200000
180000
160000
140000
120000
100000
80000
60000
40000
20000
0
-20000
-40000
-60 -70 -80 -90 -100 -110 -120 -130 -140 -150 -160 -170 -180 -190 -200 -210 -220
f1 (ppm)

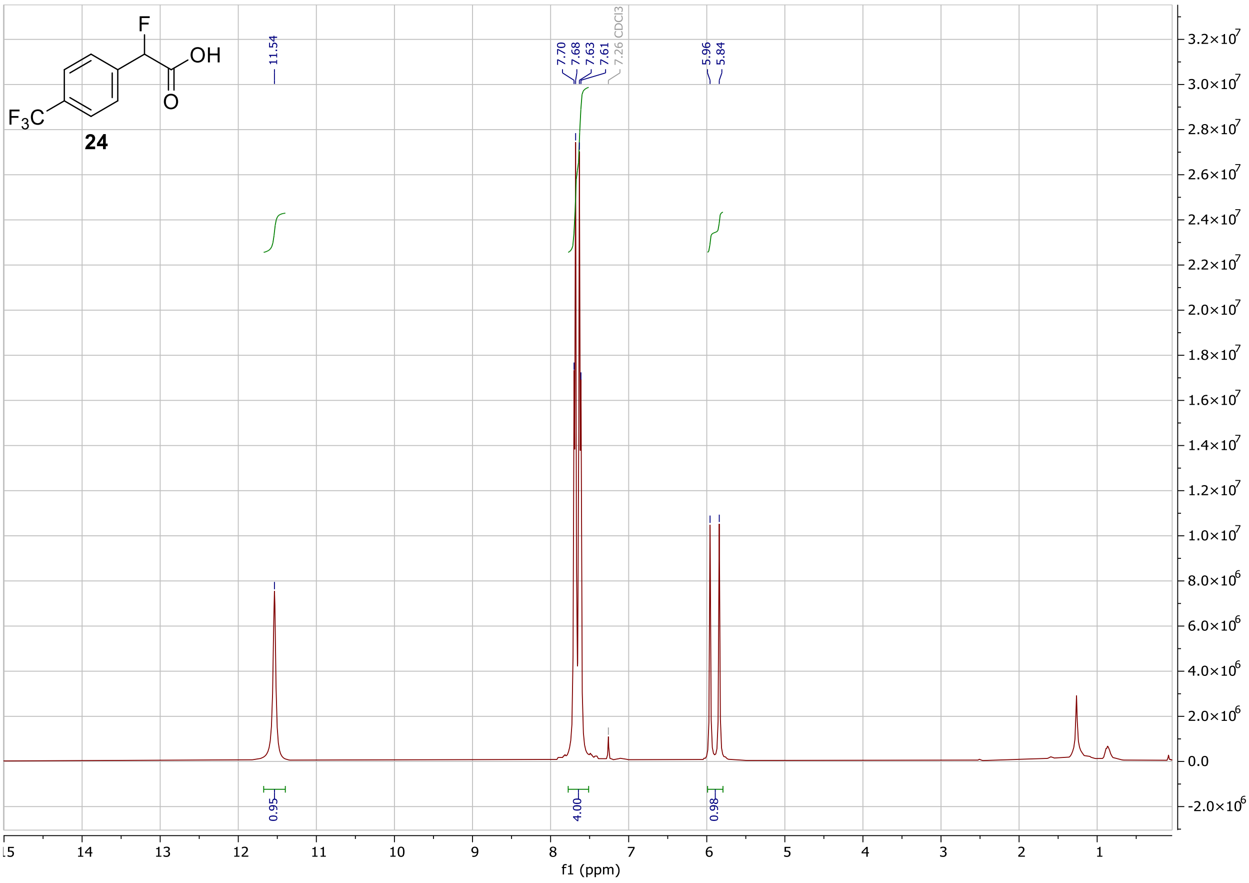
F
OH
O
$F_3C$
24
11.54
7.70
7.68
7.63
7.61
7.26 CDCl3
5.96
5.84
0.95
4.00
0.98
f1 (ppm)

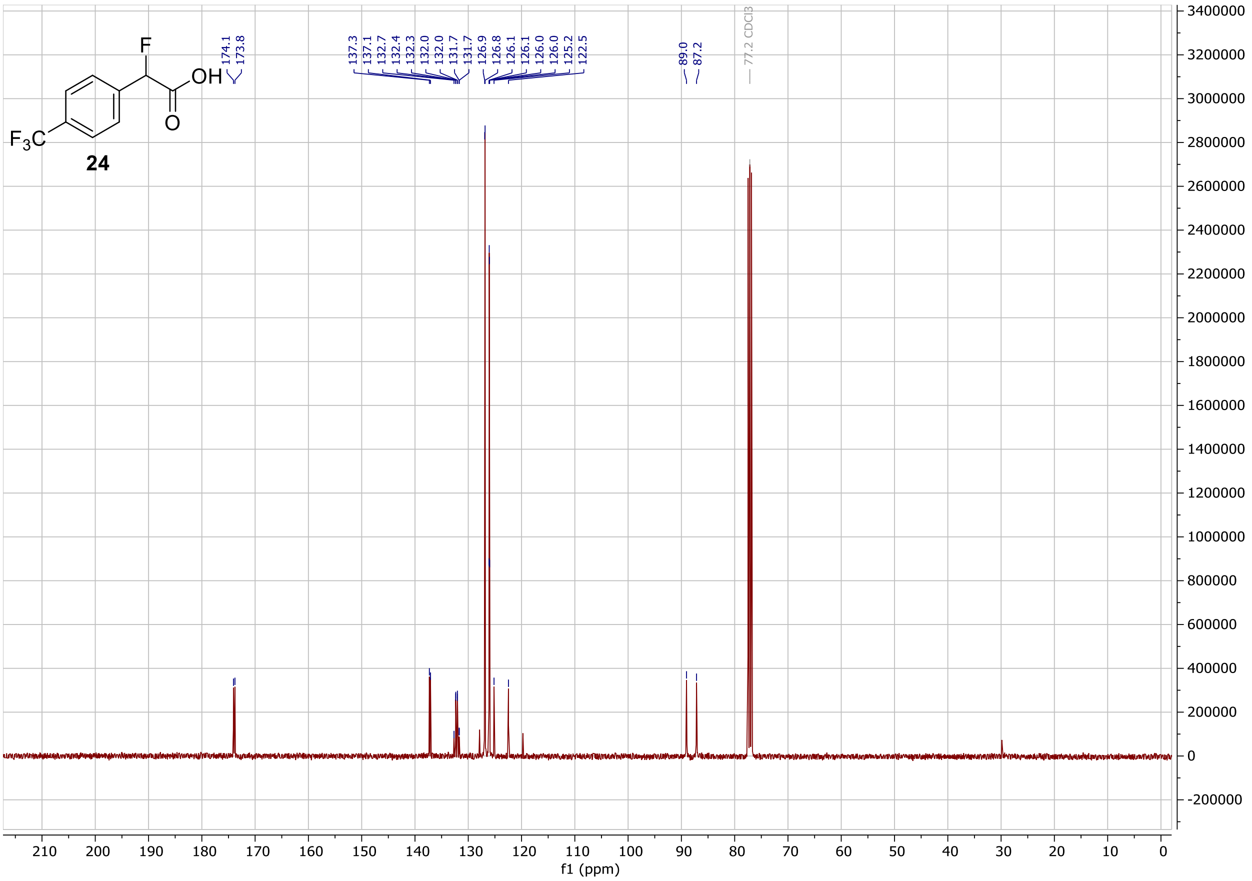
F
OH
O
$F_3C$
24
174.1
173.8
137.3
137.1
132.7
132.4
132.3
132.0
132.0
131.7
131.7
126.9
126.8
126.1
126.1
126.0
126.0
125.2
122.5
89.0
87.2
77.2 CDCl3
f1 (ppm)

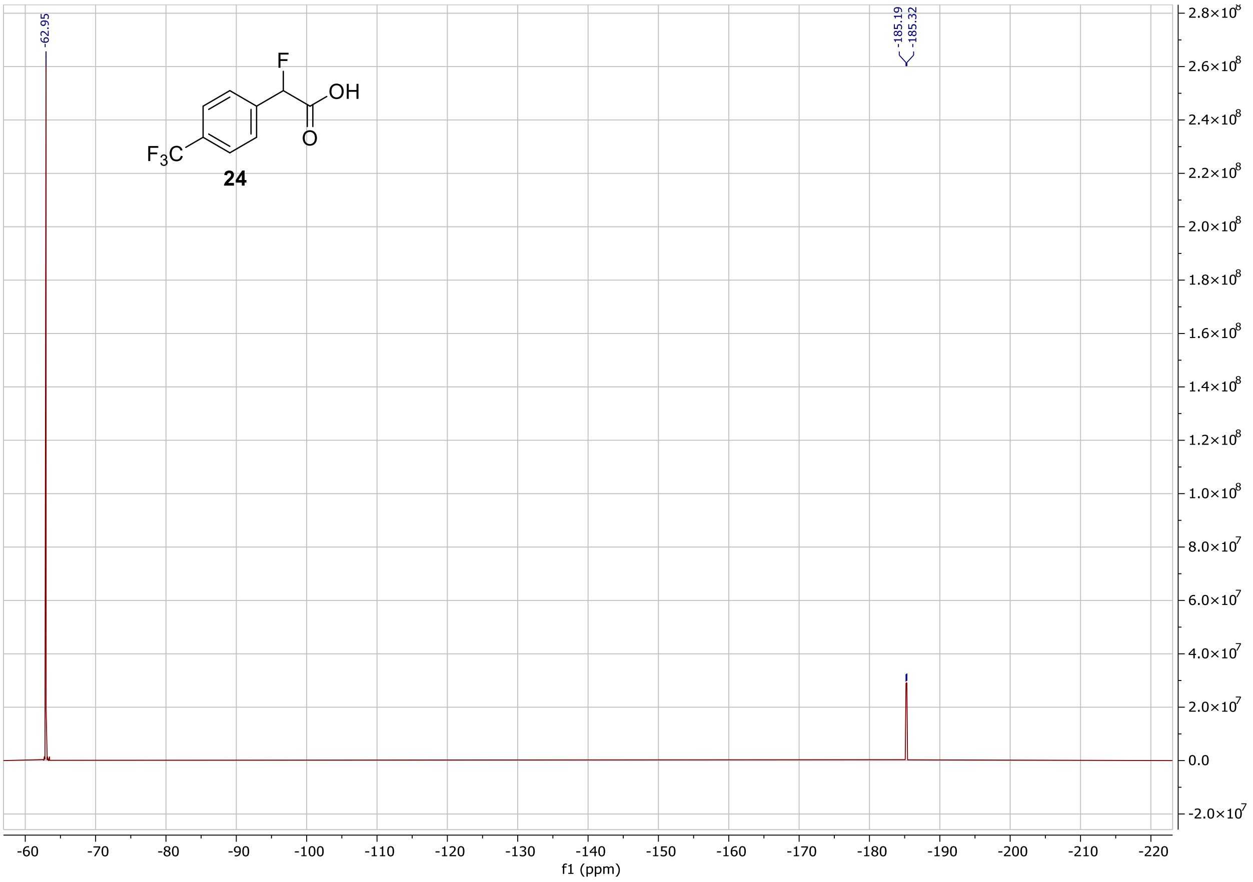
-62.95
-185.19
-185.32
F
OH
O
$F_3C$
24
f1 (ppm)

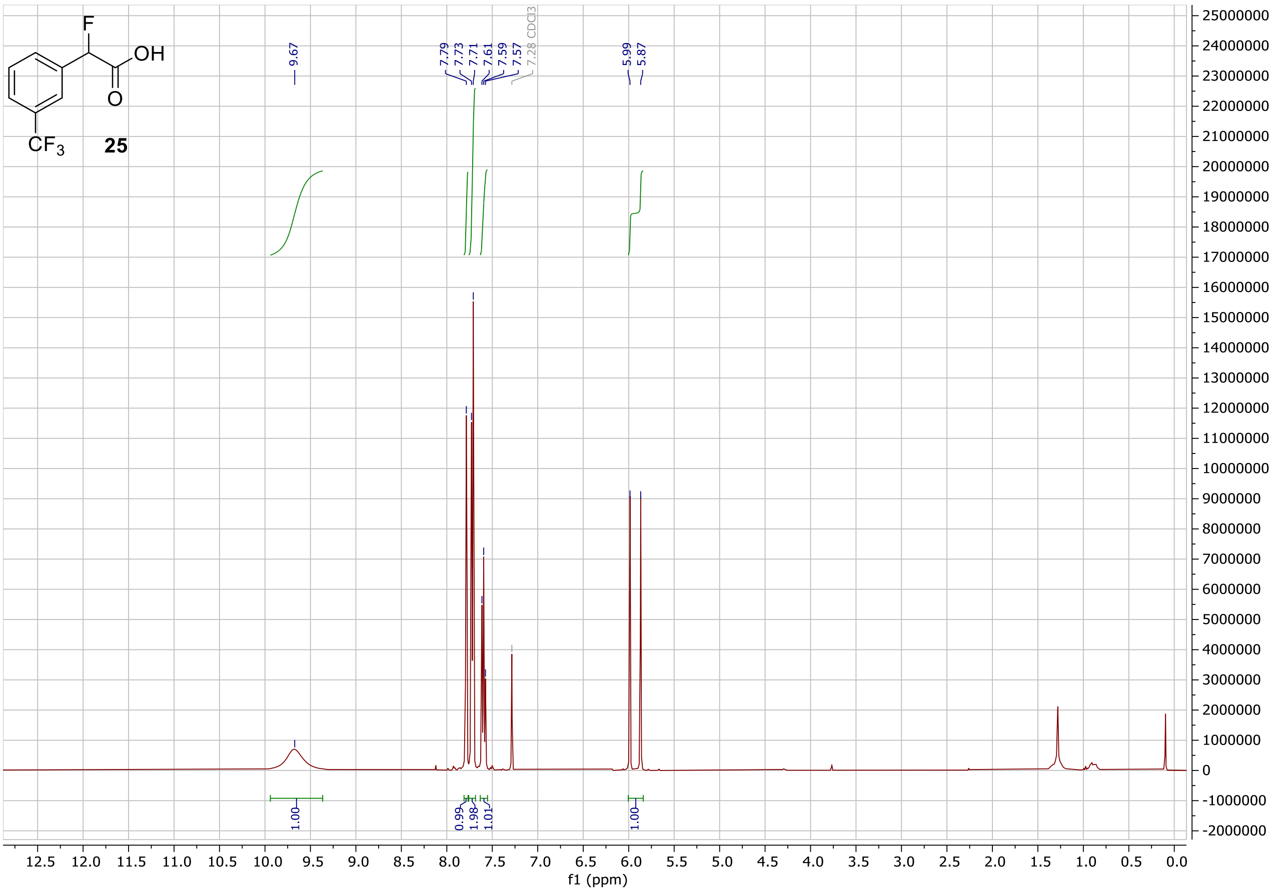
F
OH
O
CF3
25
9.67
7.79
7.73
7.71
7.61
7.59
7.57
7.28 CDCl3
5.99
5.87
1.00
0.99
1.98
1.01
1.00
f1 (ppm)

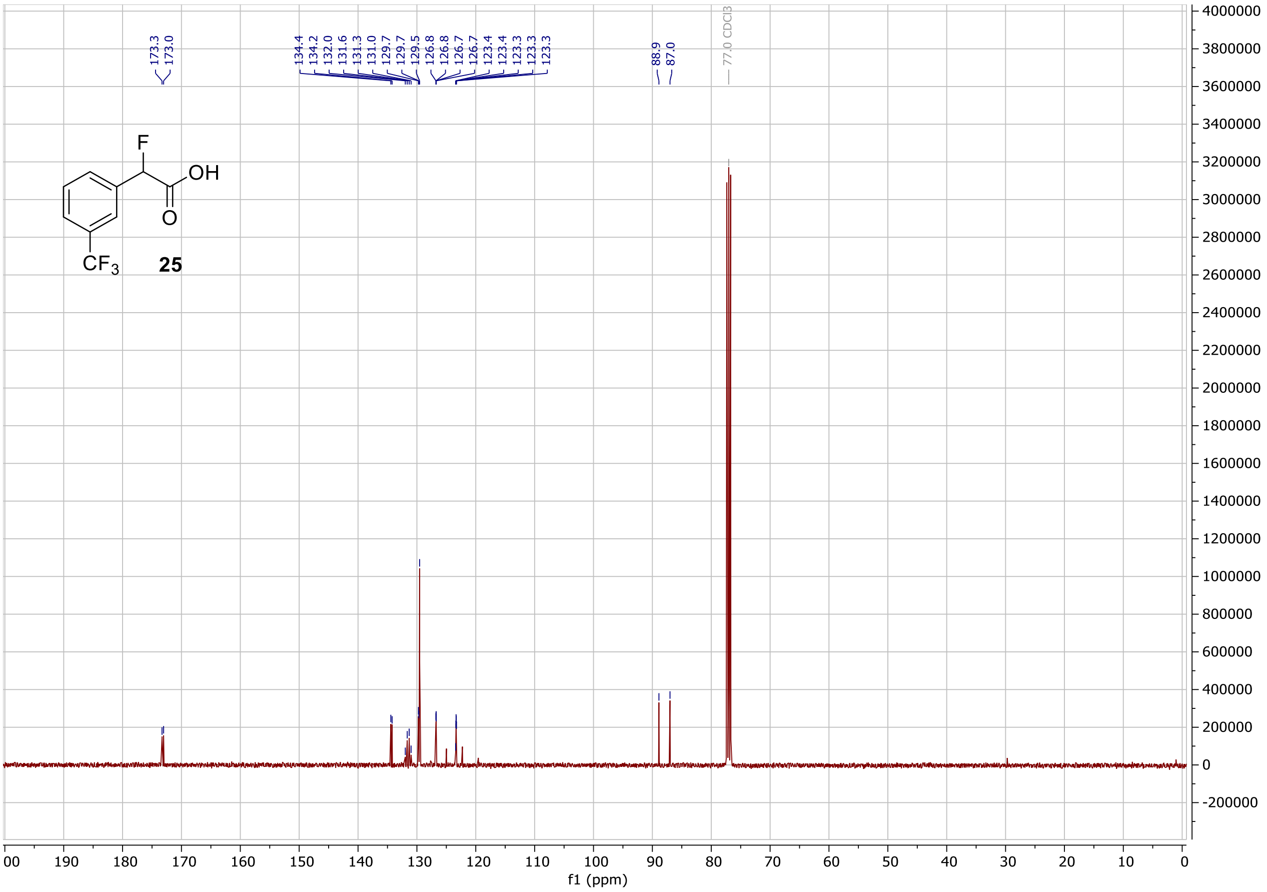
173.3
173.0
134.4
134.2
132.0
131.6
131.3
131.0
129.7
129.7
129.5
126.8
126.8
126.7
126.7
123.4
123.4
123.3
123.3
123.3
88.9
87.0
77.0 CDCl3
F
OH
O
CF3
25
f1 (ppm)

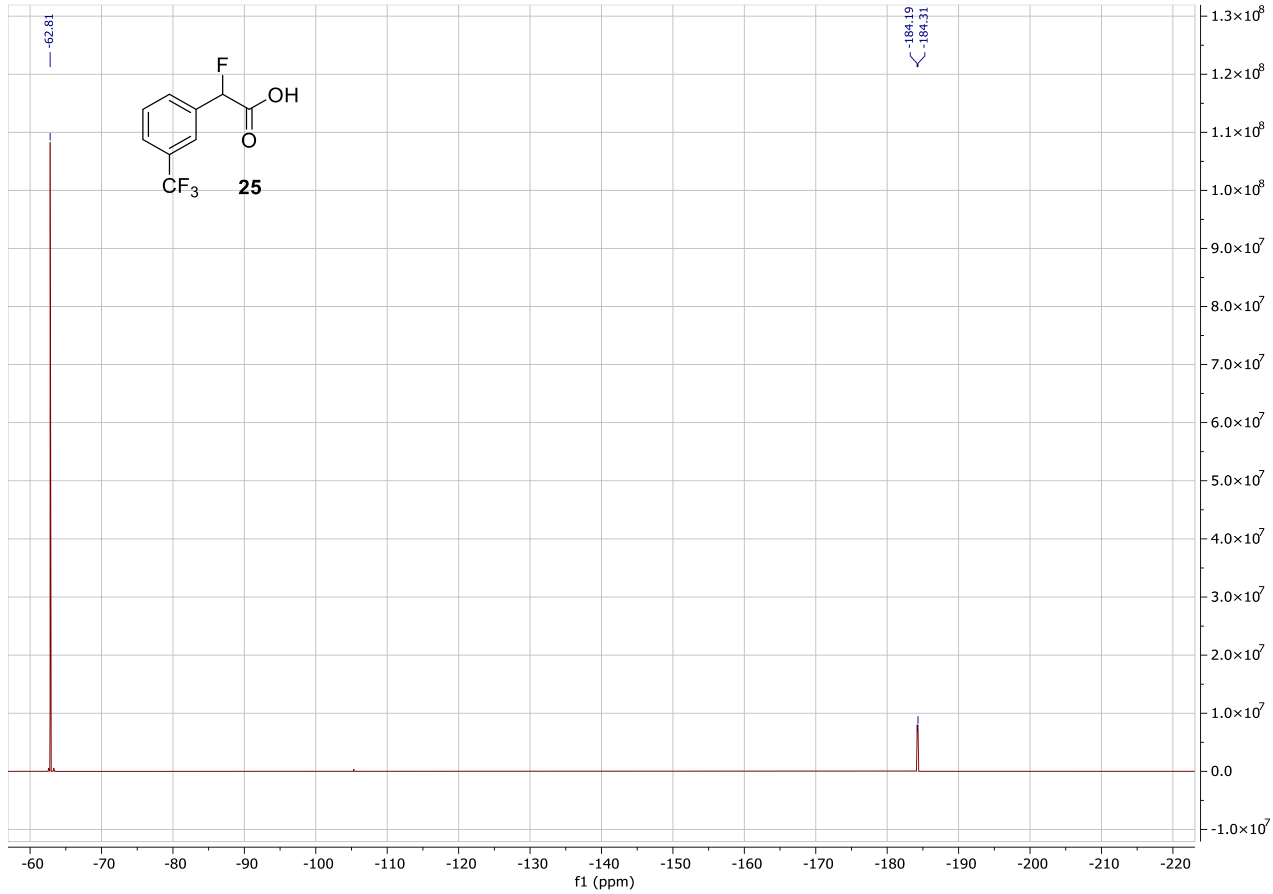
-62.81
-184.19
-184.31
F
OH
O
CF3
25
f1 (ppm)

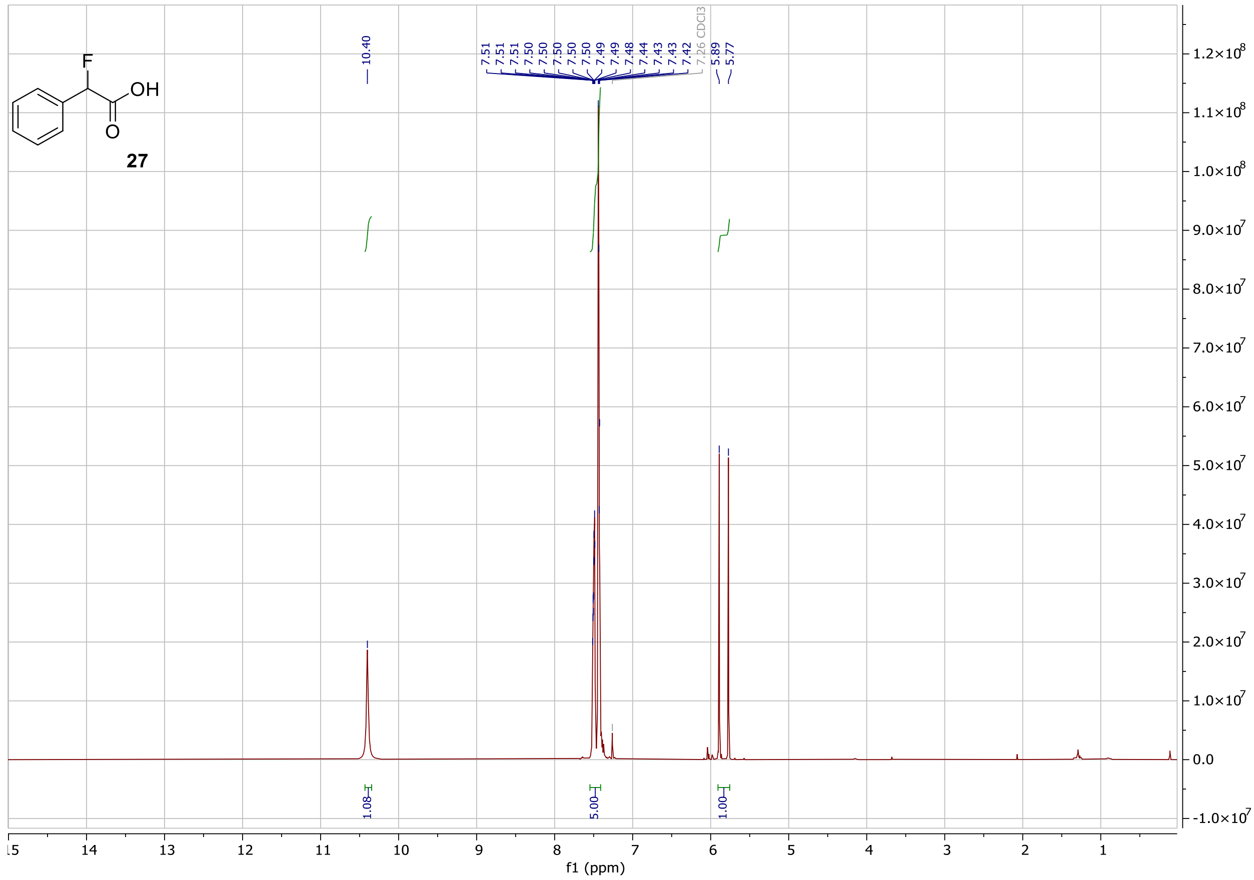
F
OH
O
27
10.40
7.26 CDCl3
5.89
5.77
1.08
5.00
1.00
f1 (ppm)

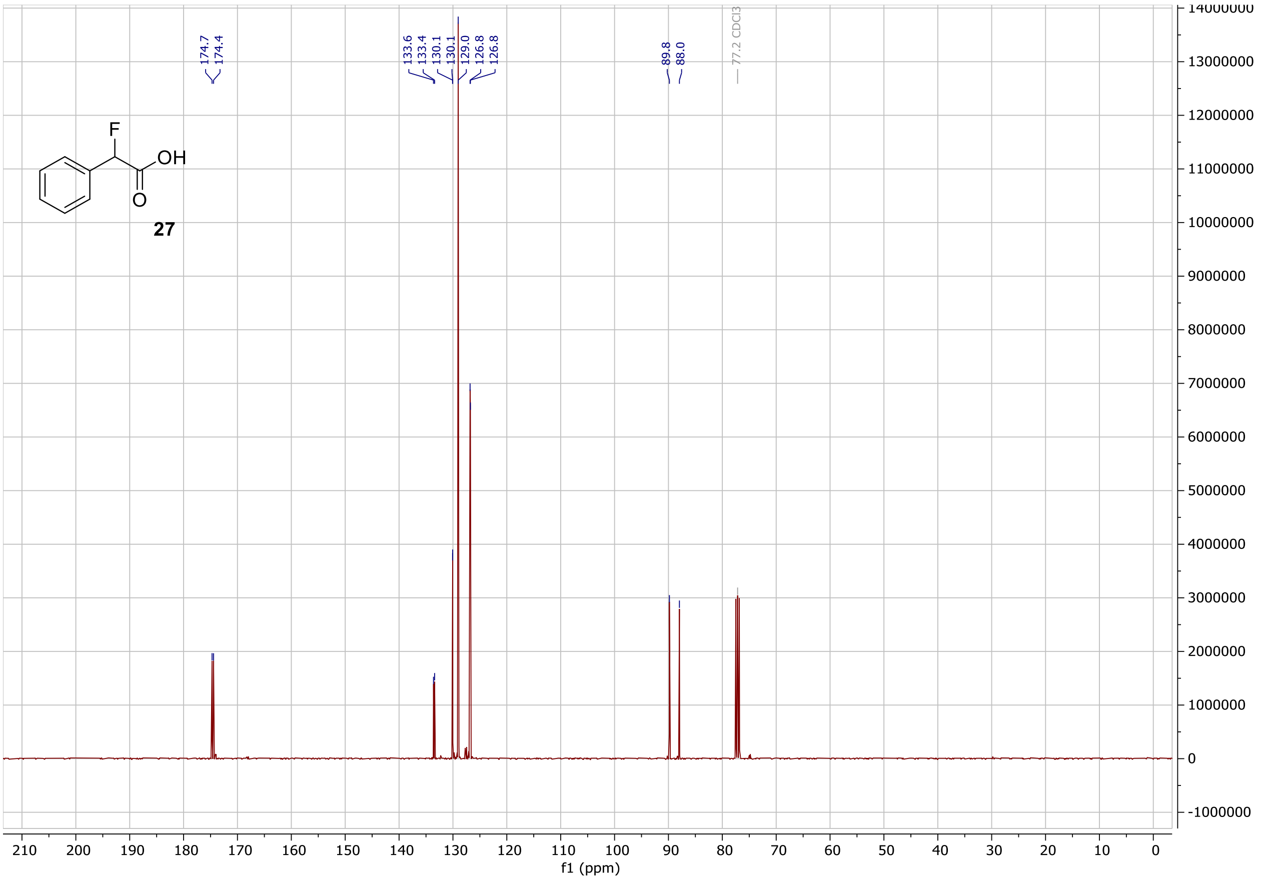
174.7
174.4
133.6
133.4
130.1
130.1
129.0
126.8
126.8
89.8
88.0
77.2 CDCl3
F
OH
O
27
f1 (ppm)

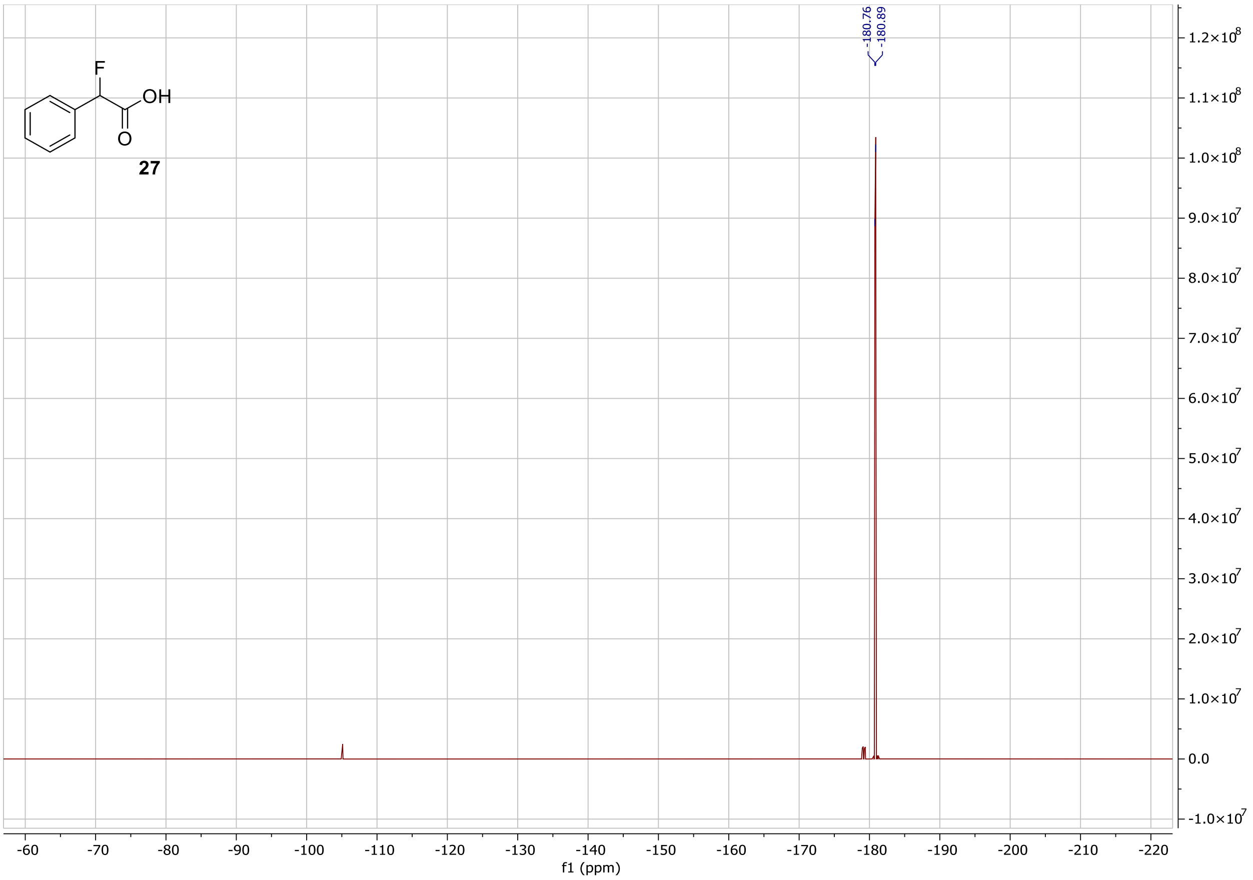
-180.76
-180.89
F
OH
O
27
f1 (ppm)

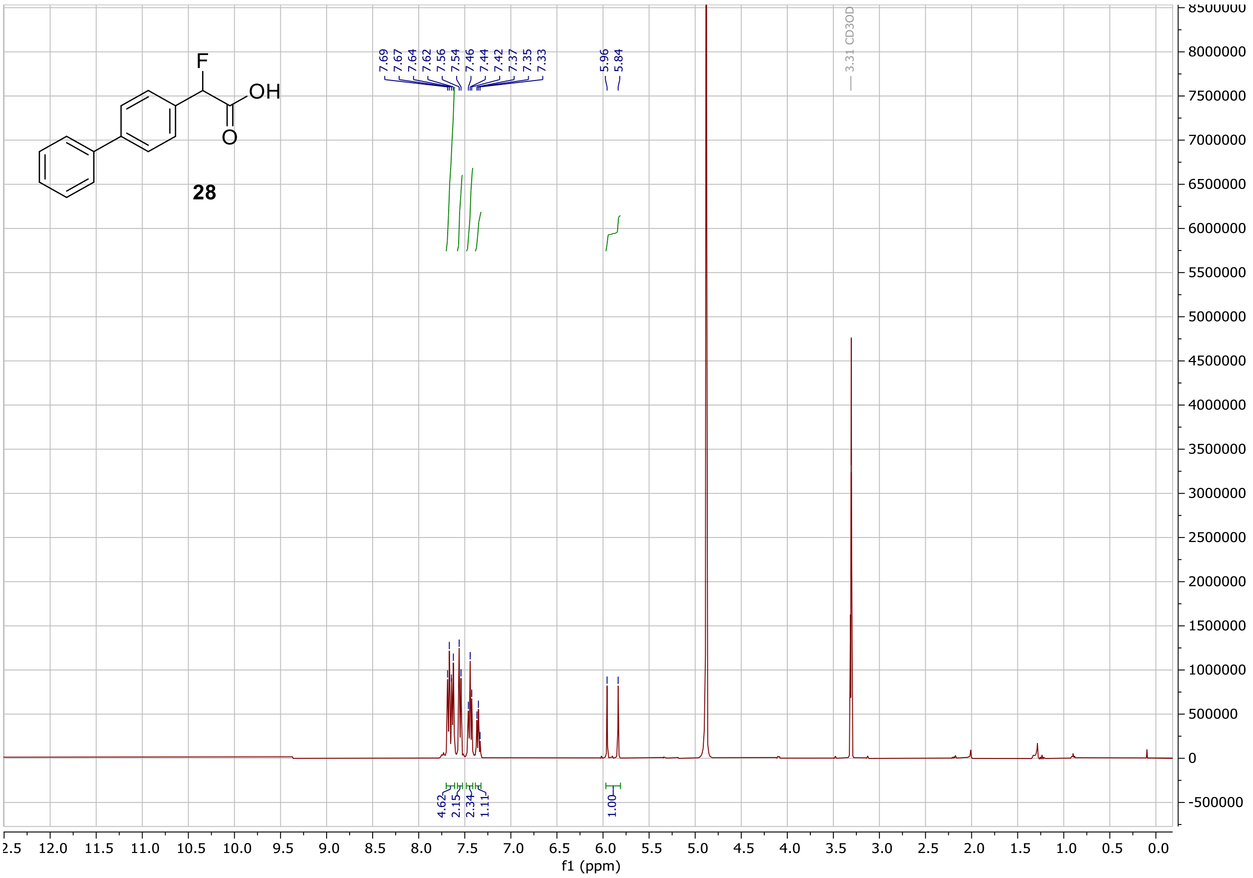
F
OH
O
28
7.69
7.67
7.64
7.62
7.56
7.54
7.46
7.44
7.42
7.37
7.35
7.33
5.96
5.84
3.31 CD3OD
4.62
2.15
2.34
1.11
1.00
f1 (ppm)

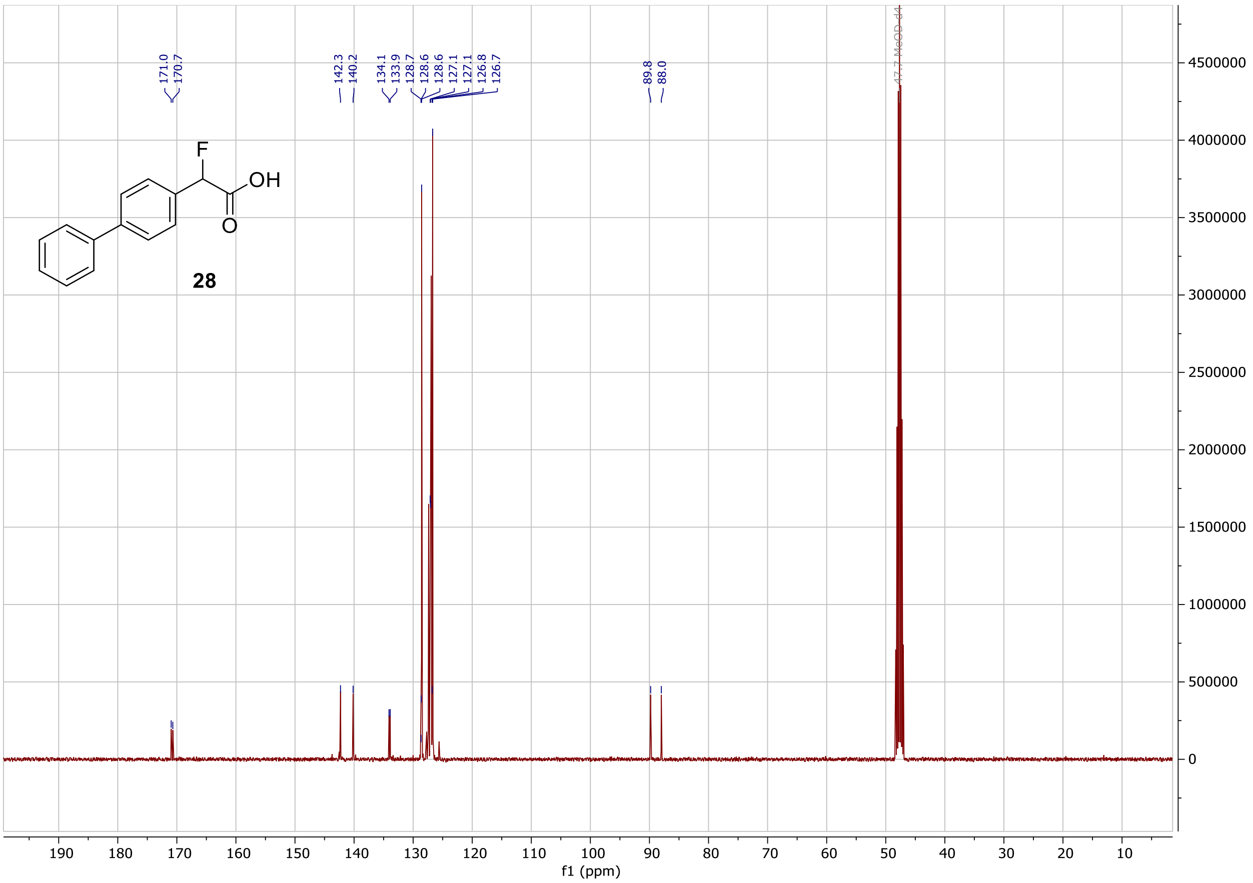
F
OH
O
28
171.0
170.7
142.3
140.2
134.1
133.9
128.7
128.6
128.6
127.1
127.1
126.8
126.7
89.8
88.0
47.7 MeOD-d4
f1 (ppm)

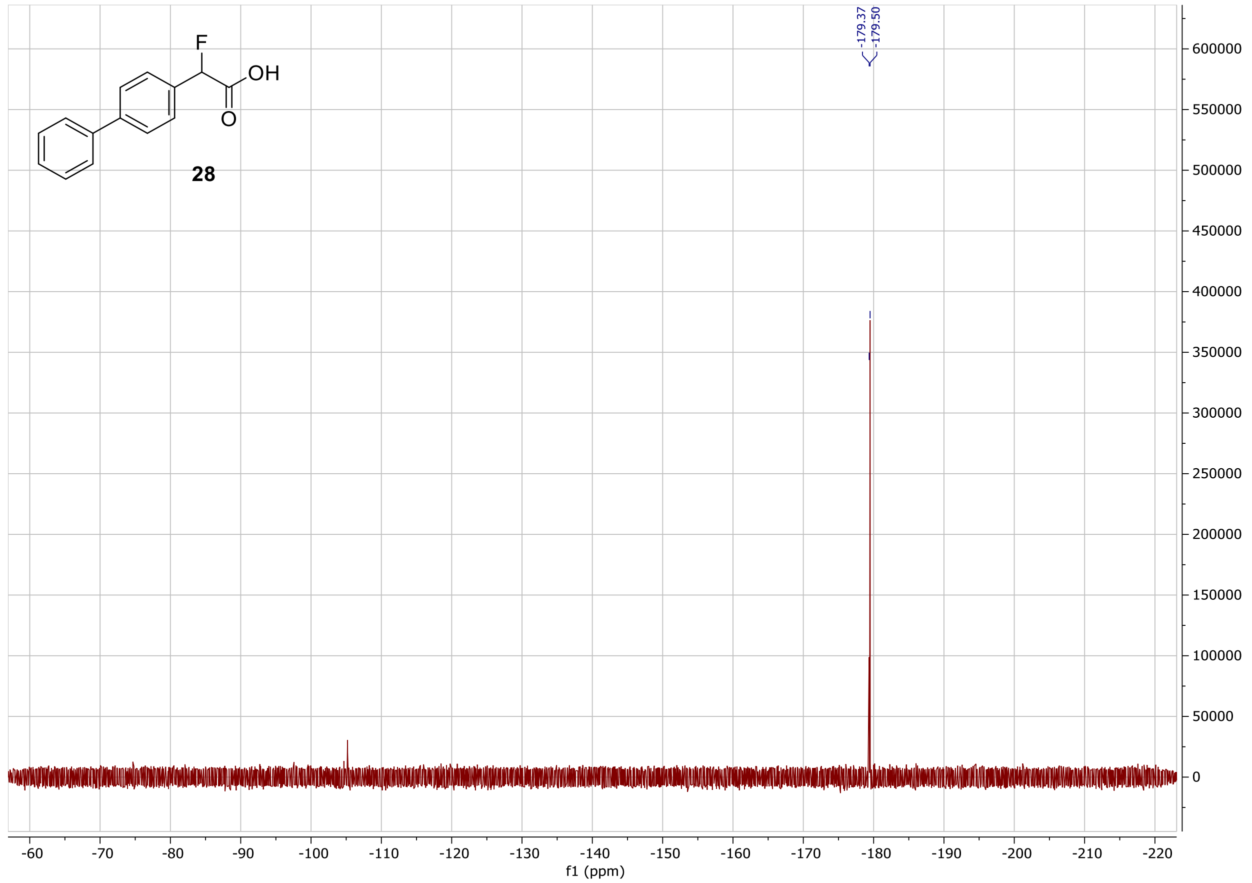

F
OH
O
28
-179.37
-179.50
f1 (ppm)


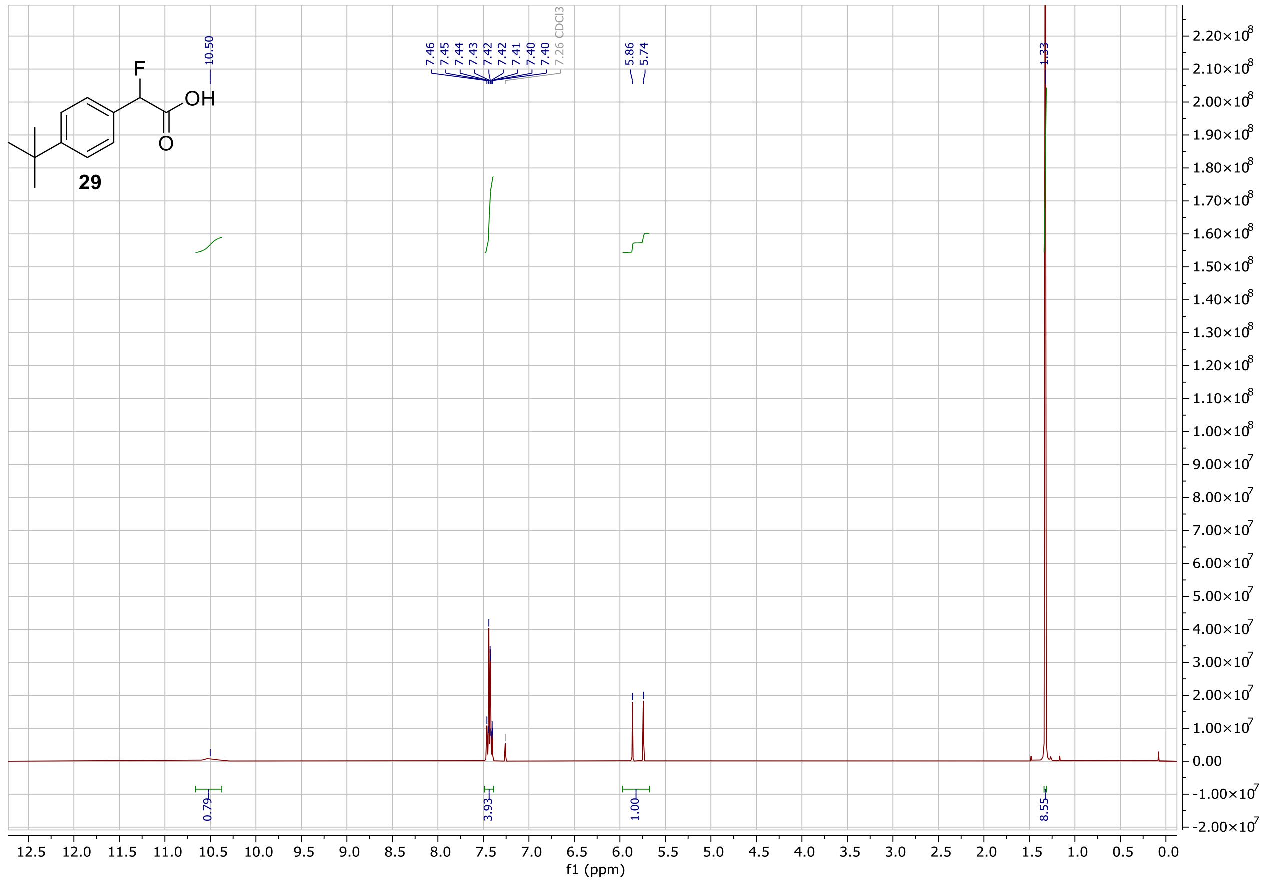

F
OH
O
29
10.50
7.46 7.45 7.44 7.43 7.42 7.42 7.41 7.40 7.40
7.26 CDCl3
5.86 5.74
1.33
0.79
3.93
1.00
8.55
f1 (ppm)

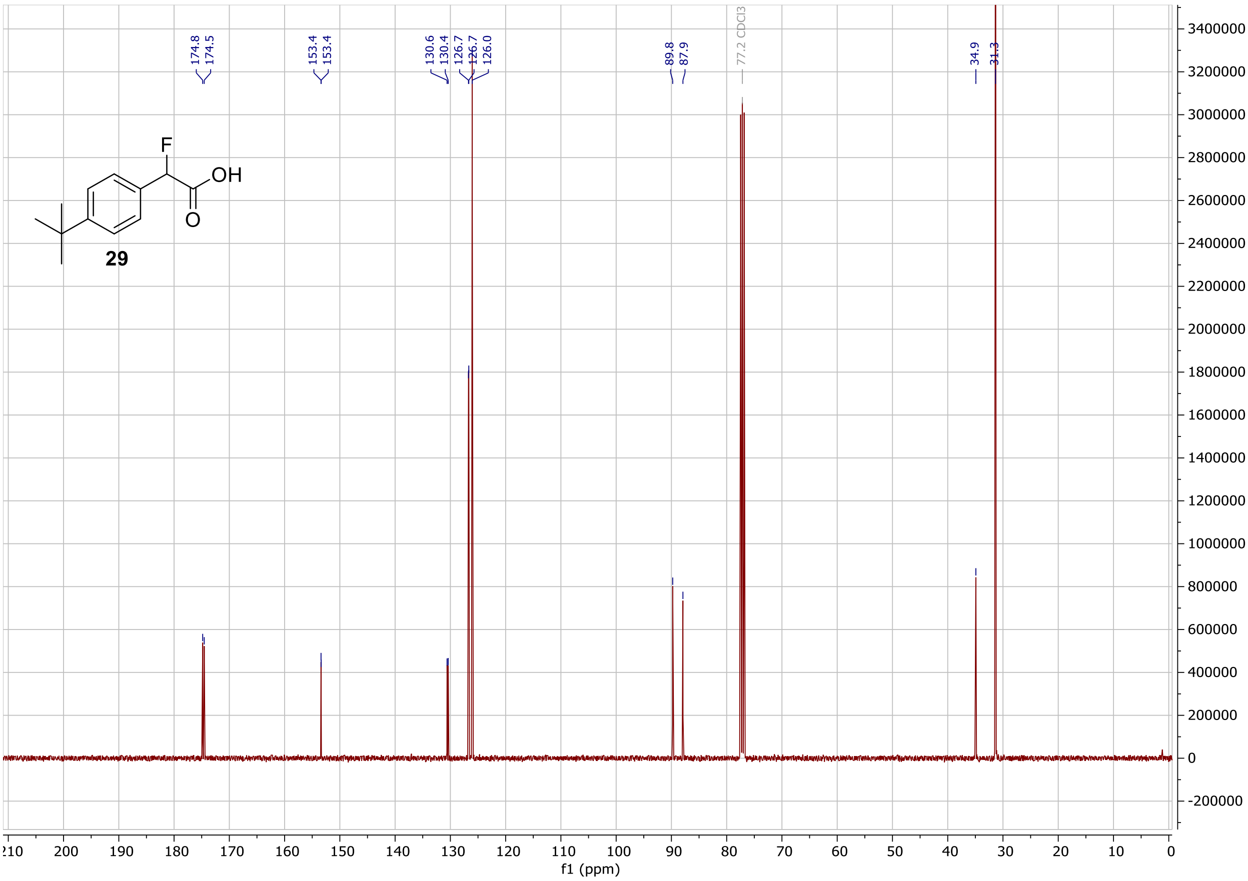

F
OH
O
29
174.8
174.5
153.4
153.4
130.6
130.4
126.7
126.7
126.0
89.8
87.9
77.2 CDCl3
34.9
31.3
f1 (ppm)


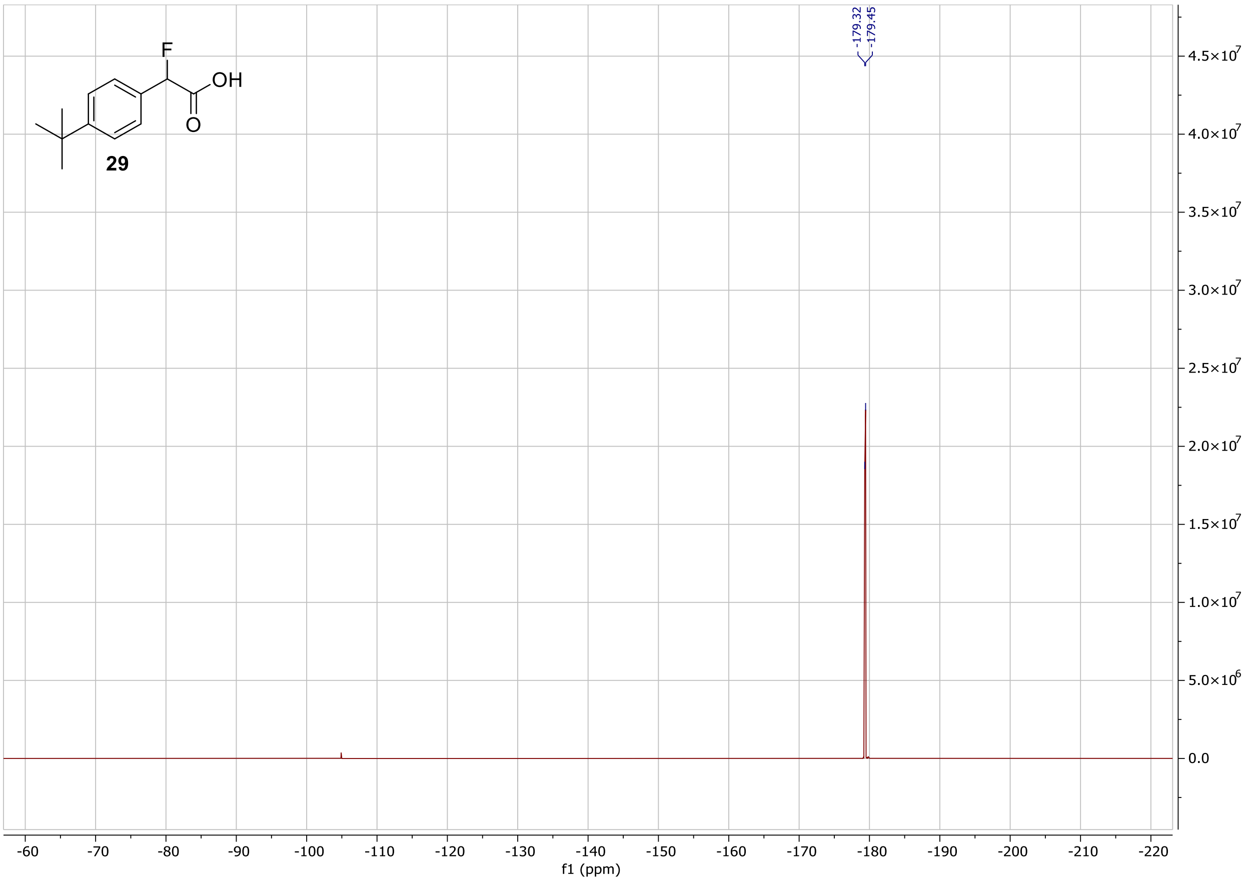

F
OH
O
29
-179.32
-179.45
f1 (ppm)
